# Ubiquitylation of BBSome is required for ciliary assembly and signaling

**DOI:** 10.15252/embr.202255571

**Published:** 2023-02-06

**Authors:** Francesco Chiuso, Rossella delle Donne, Giuliana Giamundo, Laura Rinaldi, Domenica Borzacchiello, Federica Moraca, Daniela Intartaglia, Rosa Iannucci, Emanuela Senatore, Luca Lignitto, Corrado Garbi, Paolo Conflitti, Bruno Catalanotti, Ivan Conte, Antonio Feliciello

**Affiliations:** ^1^ Department of Molecular Medicine and Medical Biotechnology University of Naples “Federico II” Naples Italy; ^2^ Telethon Institute of Genetics and Medicine Pozzuoli Italy; ^3^ Department of Biology University of Naples Federico II Naples Italy; ^4^ Department of Pharmacy University of Naples “Federico II” Naples Italy; ^5^ Net4Science srl University “Magna Graecia” of Catanzaro Catanzaro Italy; ^6^ Cancer Research Center of Marseille (CRCM) CNRS, Aix Marseille Univ, INSERM, Institut Paoli‐Calmettes Marseille France; ^7^ Faculty of Biomedical Sciences, Institute of Computational Science Università della Svizzera Italiana (USI) Lugano Switzerland

**Keywords:** BBSome, cAMP, cilium, praja2, ubiquitin, Cell Adhesion, Polarity & Cytoskeleton, Post-translational Modifications & Proteolysis, Signal Transduction

## Abstract

Bardet‐Biedl syndrome (BBS) is a ciliopathy characterized by retinal degeneration, obesity, renal abnormalities, postaxial polydactyly, and developmental defects. Genes mutated in BBS encode for components and regulators of the BBSome, an octameric complex that controls the trafficking of cargos and receptors within the primary cilium. Although both structure and function of the BBSome have been extensively studied, the impact of ubiquitin signaling on BBSome is largely unknown. We identify the E3 ubiquitin ligase PJA2 as a novel resident of the ciliary compartment and regulator of the BBSome. Upon GPCR‐cAMP stimulation, PJA2 ubiquitylates BBSome subunits. We demonstrate that ubiquitylation of BBS1 at lysine 143 increases the stability of the BBSome and promotes its binding to BBS3, an Arf‐like GTPase protein controlling the targeting of the BBSome to the ciliary membrane. Downregulation of *PJA2* or expression of a ubiquitylation‐defective BBS1 mutant (BBS1^K143R^) affects the trafficking of G‐protein‐coupled receptors (GPCRs) and Shh‐dependent gene transcription. Expression of BBS1^K143R^ *in vivo* impairs cilium formation, embryonic development, and photoreceptors' morphogenesis, thus recapitulating the BBS phenotype in the medaka fish model.

## Graphical abstract

PJA2 is a novel resident of the ciliary compartment. Upon GPCR‐cAMP stimulation, PJA2 ubiquitylates BBSome subunits and controls the assembly and disassembly of BBSome at cilia. Defects in ubiquitylation may induce Bardet‐Biedl syndrome in medaka fish.

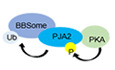

## Introduction

Bardet‐Biedl syndrome (BBS) belongs to a group of genetic syndromes, also known as ciliopathies, characterized by structural and functional abnormalities of the primary cilium (Badano *et al*, 2006). BBS is an autosomal recessive disorder displaying extremely variable clinical features in patients, even among members of the same family group. In particular, BBS patients often show loss of vision due to degeneration of photoreceptors, childhood obesity, type 2 diabetes, loss of smell, postaxial polydactyly, kidney abnormalities, impaired learning and speech, and other developmental problems (Chandra *et al*, 2022). Mutations of at least 22 different BBS genes have been identified and causally linked to the disease (McConnachie *et al*, 2021; Chandra *et al*, 2022). Among these genes, BBS1 and BBS10 mutations represent about 50% of all cases of Bardet‐Biedl syndrome (Khan *et al*, 2016). BBS gene products are components and regulators of the BBSome, an octameric protein complex localized at the basal body, which plays a central role in cargos trafficking to the primary cilium (Nachury, 2018). Mutation of each of BBS genes dramatically affects BBSome assembly and/or activity, with major effects on ciliary trafficking and cilia‐mediated signaling pathways. BBSome‐mediated cargo recognition requires membrane targeting of the complex through direct interaction of BBS1 to BBS3/Arl6, an ADP‐ribosylation factor (ARF)‐like 6 with intrinsic GTPase activity that links ciliary proteins to the intraflagellar transport (IFT) machinery. Once assembled, BBSome acquires an auto‐inhibited closed conformation. Arl6 binding to the BBSome in the cell body is an evolutionary conserved mechanism for the recruitment of the BBSome complex to the basal body and then to the ciliary compartment, both in mammalian cells and lower eukaryotes (Jin *et al*, 2010; Xue *et al*, 2020). In this compartment, Arl6^GTP^‐bound BBSome undergoes polymerization, which triggers the formation of a coat complex that crosses the transition zone and directs membrane proteins to primary cilia (Jin *et al*, 2010; Ye *et al*, 2018).

The ubiquitin‐proteasome system is an important regulatory system that controls essential aspects of cell biology (Ciechanover, 2003, 2015). Ubiquitylation involves the covalent attachment of ubiquitin, a 76 amino acid residue polypeptide, to the ε‐amine of lysine residues of target proteins. This process is coordinated through sequential ATP‐dependent enzymatic steps catalyzed by E1 (ubiquitin‐activating), E2 (ubiquitin‐conjugating), and E3 (ubiquitin‐ligating) enzymes (Ciechanover, 2003). Once ubiquitylated, the target protein may undergo proteolysis through the proteasome machinery. Alternatively, ubiquitylated proteins can follow nondegradative pathways involved in protein–protein interaction, trafficking, or protein activity (Bonifacino & Weissman, 1998). Evidence indicates that the ubiquitin pathway is relevant for cilium physiology. Several E3 ubiquitin ligases have been found to be recruited at the ciliary compartment during receptor activation, including TRIM32/BBS11, a Really Interesting New Gene (RING) E3 ligase mutated in BBS patients, and axotrophin/MARCH7, a membrane‐bound RING E3 ubiquitin ligase regulating cilia loss via ubiquitylation and degradation of nephrocystin 5 (NPHP5; Das *et al*, 2017). Moreover, IFT20‐mediated recruitment of c‐Cbl E3 ubiquitin ligases within the primary cilium is required for ubiquitylation and proteolysis of activated platelet‐derived growth factor receptor α (PDGFRα), subserving as an important negative feedback mechanism of receptor activation (Schmid *et al*, 2018). Recent studies suggest a regulatory role of ciliary UbK63 linkage in membrane receptors' ciliary trafficking and signaling (Shinde *et al*, 2020): Thus, K63‐polyubiquitylation of ciliary G‐protein‐coupled receptors (GPCRs) by β‐arrestin‐dependent ubiquitylation induces BBSome‐dependent exit of the receptors out of the cilia and interfering with the ubiquitylation machinery at the ciliary compartment markedly impairs the exit of activated GPCRs from the cilia.

PJA2 is a RING E3 ubiquitin ligase that acts as an A‐Kinase anchor protein binding and targeting the protein kinase A (PKA) to specific intracellular compartments, thus juxtaposing the kinase in close proximity of its substrates and effectors (Lignitto *et al*, 2011b). During GPCR‐cAMP stimulation, PJA2 efficiently links proteolysis of the inhibitory PKA regulatory subunits to the release of the active catalytic (C) subunits, optimizing phosphorylation and downstream activation of PKA substrates (Lignitto *et al*, 2011a). PJA2 also promotes ubiquitylation and degradation of other signaling enzymes and scaffold proteins regulating tumor suppressor pathways, mitogenic cascades, metabolism, and neuronal differentiation (Lignitto *et al*, 2013; Sakamaki *et al*, 2014; Sepe *et al*, 2014; Zhang *et al*, 2015; Rinaldi *et al*, 2016; Faust *et al*, 2017; Zhong *et al*, 2017; Song *et al*, 2019; Kattan *et al*, 2022). Recently, PJA2/PKA complex has been identified as a component of a scaffold platform assembled at the centrosome/basal body by TBC1D31 (Senatore *et al*, 2021). This complex includes also OFD1, a component of the centrosome/basal body and pericentriolar satellites that is mutated in the orofacial digital type I syndrome (OFDI). This complex finely couples GPCR signaling to ubiquitylation and proteolysis of OFD1 with important implications on cilium dynamics (Senatore *et al*, 2021). Contribution of cAMP signaling to cilium biology and dynamics triggers different effects depending on where and how cAMP signals are generated; local synthesis of cAMP by ciliary adenylate cyclases increases ciliary trafficking and axonemal length, whereas the production of cAMP at the cell body by nonciliary cyclases reduces cilium length (Porpora *et al*, 2018; Hansen *et al*, 2020; Truong *et al*, 2021; Wachten & Mick, 2021).

Here, we report a novel control mechanism of ciliary trafficking based on cAMP‐mediated ubiquitylation of BBSome complex by PJA2. Interfering with this regulatory system impairs cilium elongation, GPCRs trafficking, and signaling, with major effects on medaka fish development and retinal cell differentiation. These findings contribute to improve our understanding of the homeostatic mechanisms underlying cilium biology and highlight a potential pathogenic role of derangement of this control mechanism in human ciliopathies, such as Bardet‐Biedl syndrome.

## Results

### The ubiquitin ligase PJA2 interacts with the cilium BBSome

**Figure 1:**
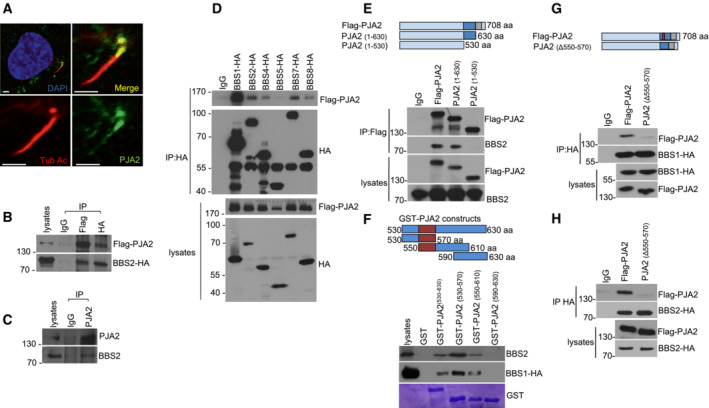
PJA2 interacts with the BBSome complex ADouble immunostaining analysis in retinal cells (ARPE‐19) for PJA2 (green) and acetylated tubulin (red). Nuclei were stained with DAPI (blue). Scale bar, 3 μm.BHEK293 cells were transiently co‐transfected with flag‐PJA2 and BBS2‐HA vectors. Lysates were immunoprecipitated with the indicated antibody. The precipitates and lysates were immunoblotted for HA and flag.CHEK293 cell lysates were subjected to immunoprecipitation with anti‐PJA2 or control IgG. The precipitates and lysates were immunoblotted for PJA2 and BBS2.DHEK293 cells were transiently co‐transfected with flag‐PJA2 and HA‐tagged BBS transgenes. Lysates were immunoprecipitated with anti‐HA antibody. The precipitates and lysates were immunoblotted for HA and flag.EHEK293 cells were transiently co‐transfected with vectors for flag‐PJA2 (either wild‐type or deletion mutants) and HA‐tagged BBS2. Immunoprecipitation was performed with anti‐flag antibody. The precipitates and lysates were immunoblotted for HA and flag. Upper panel shows a schematic representation of PJA2 domain organization including the RING domain (gray) and the BBS‐binding domain (blue).FLysates expressing HA‐tagged BBS1 and endogenous BBS2 were subjected to pulldown assay with GST and GST‐PJA2 polypeptides. Immunoblot analysis was performed with anti‐HA and anti‐BBS2. Lower panel, Comassie blue staining of GST proteins. Upper panel, PJA2 domain organization showing the BBS‐binding segment (purple).G, HLysates expressing flag‐PJA2 or PJA2 mutant (Δ550‐570) and HA‐tagged BBS1 (G) or BBS2 (H) were subjected to immunoprecipitation with anti‐HA antibody. The precipitates and lysates were immunoblotted for HA and flag. Upper panel, PJA2 domain organization showing the domains described above.

To get insight into the mechanism of PJA2 action in ciliated cells, we first analyzed the intracellular distribution of PJA2 in retinal ARPE‐19 cells growth arrested by serum deprivation. This experimental condition induces the formation of primary cilia that can be visualized by immunofluorescence using antibodies directed against acetylated tubulin, a post‐translationally modified form of tubulin that specifically accumulates within the cilium. Serum deprivation for 36 h induced the formation of primary cilia in the majority of ARPE‐19 cells (Fig 1A). Immunostaining analysis revealed that a fraction of PJA2 localized at the primary cilium, preferentially distributed at the basal body.

To investigate the mechanism(s) of PJA2 localization at ciliary compartments and identify the relevant binding partners, we performed a yeast‐two hybrid screening using the C‐terminal segment of PJA2 as bait and a human brain cDNA library. This analysis identified the C‐terminal segment of BBS2 protein, a component of the BBSome complex, mutated in the Bardet‐Biedl syndrome type 2 (Appendix Fig S1A). Co‐immunoprecipitation showed that exogenous and endogenous PJA2 bind BBS2 proteins in HEK293 cells (Fig 1B and C). Next, we evaluated whether PJA2 interacted with other BBSome subunits. Co‐immunoprecipitation assays revealed that PJA2 also interacts with BBS1, the core component of BBSome complex, and with BBS4 and BBS7, both required for BBSome assembly and localization at the primary cilium (Fig 1D). We assessed the molecular determinants that mediate the interaction between PJA2 and BBSome subunits by deletion mutagenesis and co‐immunoprecipitation. We identified the domain of PJA2 essential for the binding to BBS1/2 (Fig 1E). Specifically, by GST pulldown we found that residues 550–570 of PJA2 mediate the binding to BBS1 and BBS2 (Fig 1F). To confirm these data, we generated and immunopurified a PJA2 deletion mutant lacking residues 550–570. In agreement with our previous analysis, PJA2 550–570 deletion mutant did not bind BBS1 and BBS2 (Fig 1G and H).

### PJA2 ubiquitylates BBSome subunits

**Figure 2:**
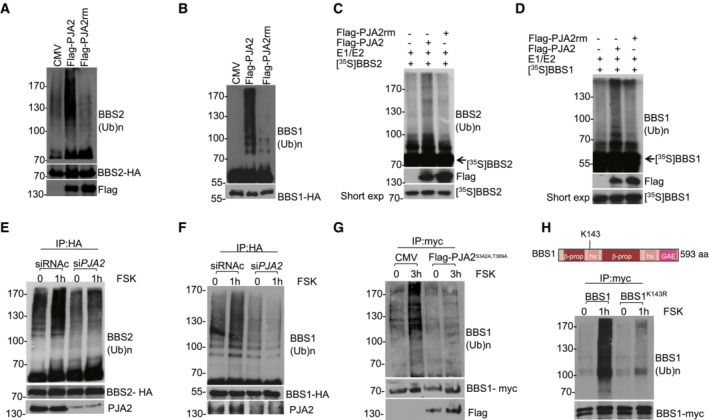
PJA2 directly ubiquitylates BBS1 and BBS2 A, BImmunoprecipitation of BBS2 (A) or BBS1 (B) from HEK293 cell lysates expressing HA‐tagged BBS1 or BBS2, ubiquitin‐myc and flag‐PJA2 (or flag‐PJA2rm). The precipitates were immunoblotted with anti‐myc (ubiquitylated BBS1 or BBS2) and anti‐HA antibodies. Flag‐PJA2 expression was analyzed in total lysates by immunoblot analysis.C, D
*In vitro* ubiquitylation assay of *in vitro* translated, ^35^S‐labeled BBS2 (C), and BBS1 (D). Translated proteins were incubated with anti‐flag precipitates (flag‐PJA2 or flag‐PJA2rm) isolated from growing cells and his_6_‐tagged ubiquitin, in the presence of E1 and UbcH5b (E2). The reaction mixture and an aliquot of ^35^S‐labeled proteins were denatured, size‐fractionated on SDS–PAGE, and analyzed by autoradiography. A fraction of the reaction mixture was immunoblotted with anti‐flag antibody (lower panel).E, FHA‐tagged BBS2 (E) or BBS1 (F) and ubiquitin‐myc vectors were co‐transfected with control siRNA (siRNAc) or siRNA targeting *PJA2* (si*PJA2*). Serum‐deprived cells were left untreated or stimulated with FSK for 1 h. Ubiquitylated BBS1 and BBS2 proteins and the levels of endogenous PJA2 and HA‐tagged expressed proteins were detected as in (A).GSame as in (F), with the exception that transfection was performed using control vector (CMV) or with a vector expressing a phosphorylation‐defective PJA2 mutant (PJA2^S342A, T389A^).HImmunoprecipitation of BBS1 and BBS1^K143R^ mutant from HEK293 cell lysates expressing myc‐tagged BBS1 variants and HA‐tagged ubiquitin. Cells were serum‐deprived overnight and left untreated or stimulated with FSK for 1 h. The precipitates were immunoblotted with anti‐HA (ubiquitylated BBS1) and anti‐myc antibodies. Upper panel shows a schematic representation of BBS1 domain organization: 7‐bladed β‐propeller (b prop), γ‐adaptin ear domain (GAE), heterodimerization helix (hx). The position of residue K143 is indicated.

We then tested whether PJA2 ubiquitylated both BBS1 and BBS2. To this end, we overexpressed wild‐type PJA2 or its inactive RING mutant (PJA2rm) in HEK293 cells and found that expression of wild‐type PJA2, not the mutant PJA2rm, significantly increased BBS1 and BBS2 ubiquitylation (Fig 2A and B). In addition, ubiquitylation experiments using *in vitro* translated, ^35^S‐labeled recombinant BBS2 and BBS1 demonstrated that both proteins (Fig 2C and D) were directly ubiquitylated by immunopurified PJA2. Since PJA2 is activated by GPCR‐cAMP (Lignitto *et al*, 2011a), we assessed whether BBS1 and BBS2 ubiquitylation was stimulated in cells exposed to forskolin, a diterpene that activates adenylyl cyclase and increases cAMP levels. As shown in Fig 2E, forskolin induced a time‐dependent ubiquitylation of BBS1 and BBS2 (Appendix Fig S1B and C), which was abrogated by *PJA2* silencing (Fig 2E and F). As PKA phosphorylation of PJA2 at residues S342 and T389 is required for its ligase activity (Lignitto *et al*, 2011a), we tested whether a PJA2 mutant in the PKA phosphorylation sites would impair BBSs ubiquitylation. Indeed, a PJA2 phosphorylation‐mutant (PJA2^S342A, T389A^) hampered FSK‐induced BBS1 ubiquitylation (Fig 2G) demonstrating that cAMP–PKA–PJA2 axis regulates the formation of the BBSome. Moreover, since ubiquitylation of BBS1 and BBS2 by cAMP–PJA2 axis was not coupled to proteolysis, it is possible that the ubiquitylation (e.g., K63) of BBSs triggered by PJA2 controls BBSome stability or/and activity (Appendix Fig S1D), as already described for other PJA2 substrates (Faust *et al*, 2017; Zhong *et al*, 2017).

Given the essential role of BBS1 in BBSome assembly and trafficking, we sought to identify the lysine residue(s) of BBS1 acting as acceptor site(s) of ubiquitin moieties and test its biological relevance in BBSome‐dependent ciliary activities. To this end, we took advantage of available databases reporting ubiquitylated lysine residues on BBS1 identified by mass spectrometry (Akimov *et al*, 2018). Accordingly, we performed a guided site‐directed mutagenesis to generate BBS1 mutants in the lysine acceptor site/s. We then tested in serum‐starved or cAMP‐stimulated cells the ubiquitylation of wild‐type or lysine‐mutants BBS1. We identified K143 as the main ubiquitin acceptor site on BBS1, since its substitution with arginine abrogated BBS1 ubiquitylation induced by cAMP (Fig 2H).

### Ubiquitylation of BBS1 is required for Arl6‐binding and BBSome complex assembly

**Figure 3:**
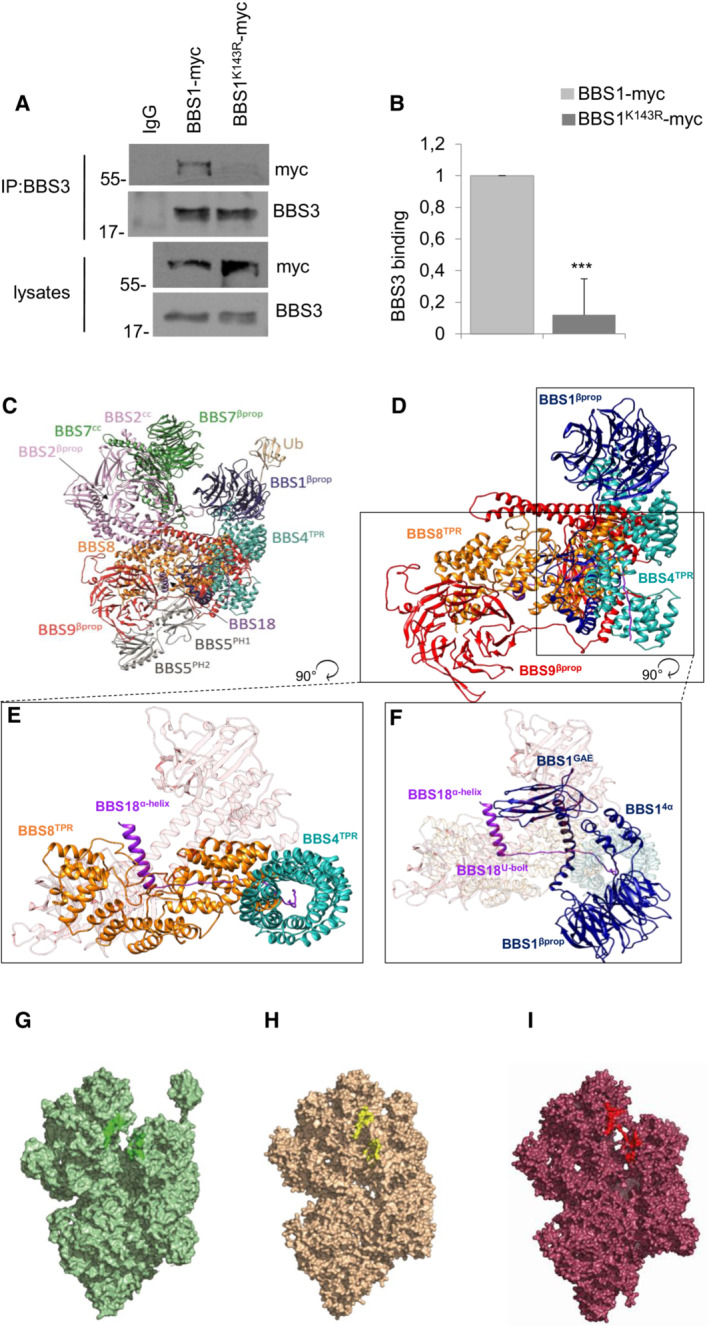
Dynamic modeling of hBBSome and Ub‐hBBSome complex ACo‐immunoprecipitation assay of BBS1 or BBS1^K143R^ and BBS3 from lysates of HEK293 transiently expressing the indicated transgenes.BCumulative data of the experiments shown in (A). The data are expressed as mean ± SD of three independent experiments. Student's *t* test, ***P < 0.001.CFull atomistic description of the homology model of the octameric complex of the BBSome human sequence (*h*BBSome) in the Arl6^GTP^‐bound (open) conformation, with the monoubiquitylation at K143 of *h*BBS1^4α^ (Ub‐*h*BBSome).DFocus on the full atomistic model of *h*BBSome core complex components (BBS1, BBS4, BBS8, BBS9, and BBS18).ETop view of the interaction details between BBS18 α‐helix and the BBS18 U‐bolt domains with the BBS8^TPR^ and the superhelix of BBS4^TPR^, respectively.FTop view of the interaction details between BBS18 α‐helix with the BBS1^GAE^ domain and the superhelix of BBS4^TPR^.GSurface representation of the CG‐MD model of the Ub‐*h*BBSome, representing the starting point of the CG‐MD simulation. Residues involved in the Arl6^GTP^‐bound binding mode are highlighted in dark green.H, ISurface representation of the last CG‐MD frame of the wt‐*h*BBSome and the Ub‐*h*BBSome, respectively showing the progressive closure of Arl6‐binding site located between BBS1^βprop^ and BBS7^βprop^. Residues involved in the Arl6^GTP^‐bound binding mode are highlighted in red and yellow, respectively.

BBS1 is the core component of the BBSome complex and the principal mediator of membrane targeting and cargo recognition (Mykytyn *et al*, 2002; Mourao *et al*, 2014). Structural studies revealed that the interaction between the N‐terminal beta‐propeller domain of BBS1 and the small GTPase Arl6 is essential for membrane targeting (Klink *et al*, 2020; Singh *et al*, 2020; Yang *et al*, 2020). K143 is located in the α‐helical insertion (4α) β‐propeller domain of BBS1, in the proximity of the Arl6‐binding interface. Accordingly, we investigated the contribution of K143 to the Arl6 binding by co‐immunoprecipitation of Arl6/BBS3 and wild‐type BBS1 or its ubiquitin‐defective mutant. The data show that the K143R mutated BBS1 did not bind efficiently Arl6/BBS3 (Fig 3A and B).

**Figure EV1:**
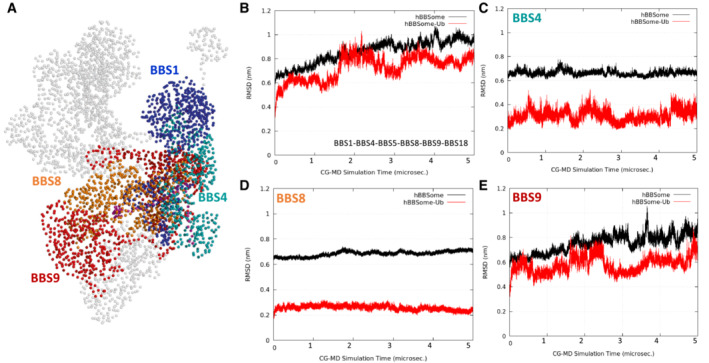
Modeling of the Ub‐*h*BBSome core complex ACG model highlighting the Ub‐*h*BBSome core complex BBS1, BBS4, BBS8, and BBS9 (CG beads are represented as spheres).BPlot of the RMSD trend of the *h*BBSome core complex (black lines) and the Ub*‐h*BBSome core complex (red lines).C–EPlot of the RMSD trend of the single *h*BBSome core complex subunits, BBS4, BBS5, BBS8, and BBS9, respectively. Each plot shows the higher stabilization of each subunit of the core complex in the monoubiquitinated CG model (red lines), with respect to the *h*BBSome wild‐type (black lines). RMSD calculations were performed on the CG‐MD production run considering only the BBSome beads (represented as spheres in panel A).

**Figure EV2:**
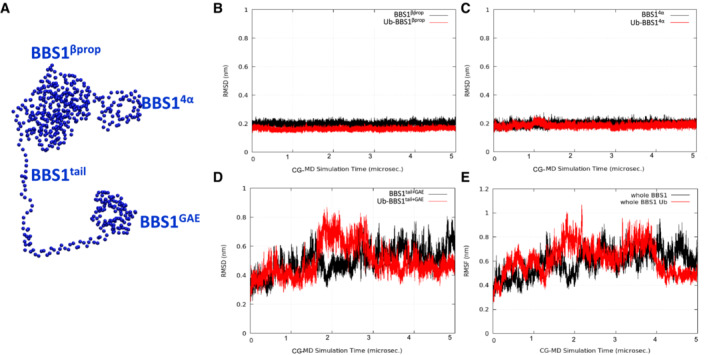
Dynamic modeling of BBS1 domains ACG beads of the BBS1 domains β‐propeller, 4α, tail, and GAE.B–DPlot of the RMSD trend of the BBS1 β‐propeller, 4α and tail+GAE domains, respectively. It can be observed that the main movements in the BBS1 subunit concern the domain formed by tail and GAE (d), while both β‐propeller and 4α domains are very stable along 5 μs of CG‐MD (b and c) in both the K143 ubiquitinated and wild‐type systems.EPlot of the RMSD trend of the entire BBS1. RMSD calculations were performed on the CG‐MD production run considering only the BBS1 beads (represented as spheres in panel A).

**Figure EV3:**
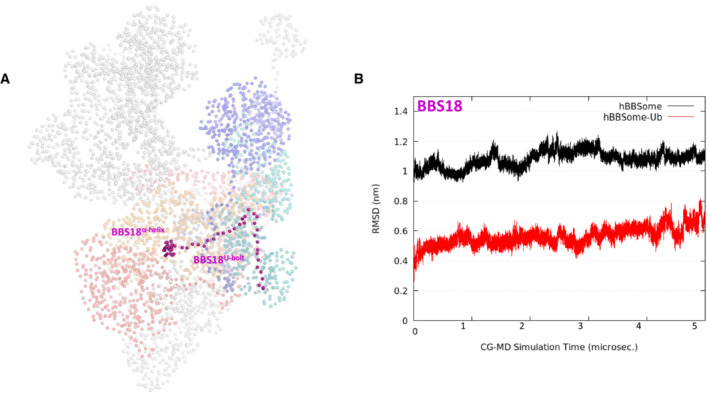
Dynamic modeling of Ub‐*h*BBSome complex CG beads of the Ub‐*h*BBSome model, highlighting the BBS18 subunit (magenta spheres).Root Mean Square Deviation (RMSD) of BBS18 subunit, highlighting the reduced mobility of *h*BBS18 residues in the K143 monoubiquitinated *h*BBSome (Ub‐*h*BBSome; red lines). RMSD calculations were performed on the CG‐MD production run considering only the BBSome beads (represented as spheres in panel A).

The effects of ubiquitylation on human BBSome (*h*BBSome) were investigated at the molecular level by microseconds‐long molecular dynamics (MD) simulations. The 3D structures of wild‐type *h*BBSome hetero‐octameric complex (wt‐*h*BBSome) and the K143 monoubiquitylation form (Ub‐*h*BBSome) were generated in an open conformation state by homology modeling (Fig 3C). Given the size of the system, to reduce its dimensionality complexity and improve the sampling of long‐timescale events, such as global conformational rearrangements, the all‐atoms structures were converted to their corresponding coarse‐grained (CG) model (Appendix Fig S2). Specifically, 5 μs of coarse‐grained molecular dynamics (CG‐MD) were simulated for each hetero‐octameric system (wt‐*h*BBSome and Ub‐*h*BBSome). The analysis of the CG‐MD simulations of both systems showed that the monoubiquitylation reduced the mobility of the protein core complex (Fig 3D–F), compared with the wt‐*h*BBSome, as shown by the analysis of the root mean square deviation (RMSD) trends over the CG‐MD simulation time (Fig EV1A–E), inducing a progressive closure of the Arl6‐binding site located between BBS1^βprop^ and BBS7^βprop^ (Fig 3G–I). Interestingly, the presence of the monoubiquitylated K143 did not significantly affect the mobility of BBS1 (Fig EV2A–E). On the contrary, a more pronounced effect on the mobility was observed for subunits BBS4, BBS8, BBS9 (Fig EV1A–E, respectively), and BBS18 (Fig EV3A and B) constituting the core of the octameric complex (Fig EV3A and B).

The atomic position Pearson correlation coefficient (aPCC) analysis provided information on an extended network of interactions found selectively in Ub‐*h*BBSome, from BBS1 to BBS4 and BBS8 through the BBS18, suggesting that these subunits constitute an axis spanning from one side of the complex to the other, for the functional communication of the protomers (Appendix Fig S3A and B). Therefore, we hypothesized that the ubiquitin‐defective BBS1 mutant could impact on the BBSome architecture, inducing alteration on the ciliary assembly and disassembly of BBSome components, which not necessarily leads to a defective BBSome protein stability or degradation. To test this hypothesis, a principal component analysis (PCA) analysis was carried out to elucidate the macroscopic effects of ubiquitination on the protein complex dynamics. The principal component (eigenvector; Fig EV1) of the wt‐*h*BBSome simulation was represented by a motion involving BBS1, BBS9, BBS18, BBS4, and BBS7, in which the BBS1 rotated along the axis defined by the heterodimerization of the helix domain, resulting into the approach of BBS1 toward BBS7 (Movie EV1). In the CG simulation of Ub*‐h*BBSome, the main movement was, instead, an anti‐clockwise rotation of BBS1^βprop^ and BBS1^4α^ domains with respect to BBS7^βprop^, with the BBS1^4α^ residues going toward BBS9 (Movie EV2). As a consequence, the CG‐MD final structures of wt‐*h*BBSome and Ub*‐h*BBSome adopted a closed conformational state (Fig 3H and I), similar to the 3D structure of the bovine closed apo form BBSome (PDB ID 6vbu; Appendix Figs S4A and B, and S5A–H). This suggests that the interaction of BBS1 with Arl6 or other cargo proteins stabilizes the open structure (Klink *et al*, 2020; Singh *et al*, 2020). Taken together, CG‐MD data show that monoubiquitylation confers more rigidity to the hetero‐octameric Ub‐*h*BBSome complex, mainly evident in the BBS18 subunit, which is a key element for BBSome assembly and disassembly. Nevertheless, our data do not show an appreciable direct effect on the Arl6‐binding site.

### Ubiquitylation of BBS1 controls ciliary trafficking of GPCRs and signaling

**Figure 4:**
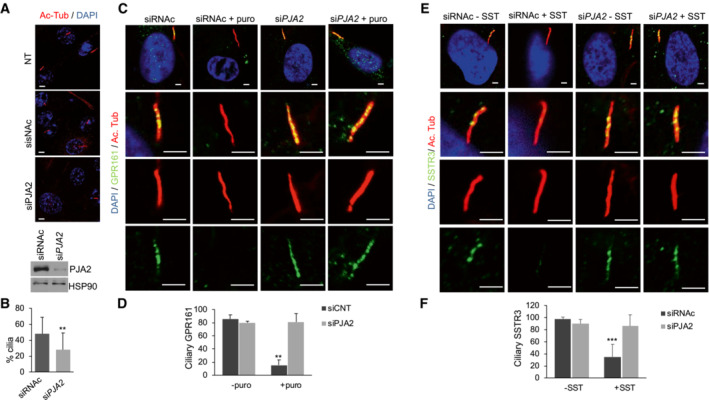
Requirement of PJA2 for ciliary trafficking ARPE‐19 cells were transiently transfected with control siRNA or siRNA targeting *PJA2* (si*PJA2*), serum‐deprived for 48 h, and immunostained for acetylated tubulin (red). Nuclei were stained with DAPI (blue). Scale bar, 3 μm. Lower panel shows western blot for PJA2.Cumulative data of the experiments shown in (A). A mean value ±SD of at least 4 independent experiments is shown. In total 25 cells for each experiment were scored. Student's *t* test, **P < 0.01.ARPE‐19 cells were transiently transfected with control siRNA or siRNA targeting *PJA2* (si*PJA2*). Cells were serum‐deprived for 48 h and left untreated or stimulated with purmorphamine (puro) for 4 h. Cells were fixed and doubly immunostained for GPR161 (green) and acetylated tubulin (red). Nuclei were stained with DAPI (blue). Scale bar, 3 μm.Cumulative data of the experiments shown in (C). A mean value ±SD of at least 4 independent experiments is shown. In total, 25 cells for each experiment were scored. Student's *t* test, **P < 0.01.ARPE‐19 cells were transiently transfected with control siRNA or siRNA targeting *PJA2* (si*PJA2*). Cells were serum‐deprived for 48 h and left untreated or stimulated with somatostatin (SST) for 3 h. Cells were fixed and doubly immunostained for SSTR3 (green) and acetylated tubulin (red). Nuclei were stained with DAPI (blue). Scale bar, 3 μm.Cumulative data of the experiments shown in (E). A mean value ±SD of at least 4 independent experiments is shown. In total 25 cells for each experiment were scored. Student's *t* test, ***P < 0.001.

**Figure 5:**
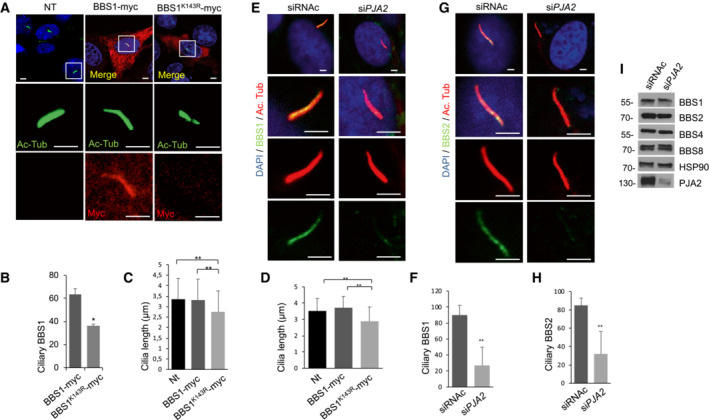
PJA2 controls ciliary localization of BBS1 and BBS2 and cilia length NIH3T3 Cells were transiently transfected with the indicated myc‐tagged vectors, serum‐deprived for 48 h, and doubly immunostained for Myc (red) and acetylated tubulin (green). Nuclei were stained with DAPI (blue). The boxes represent the magnification of the primary cilium in not transfected or transfected cells. Scale bar, 3 μm.Cumulative data of the experiments shown in (A). A mean value ± SD of four independent experiments is shown. In total 25 cells for each experiment were scored. Student's *t* test, *P < 0.05.NIH3T3 cells were transiently transfected with empty vector, Myc‐tagged BBS1 variants or Myc‐tagged BBS1‐K143R, and serum‐deprived for 48 h. Cilia length was measured with ZEN microscope tool. A mean value ± SD of three independent experiments is shown. In total 30 cells for each experiment were scored. Student's *t* test, **P < 0.01.ARPE‐19 cells were transiently transfected with empty vector, Myc‐tagged BBS1 variants or Myc‐tagged BBS1‐K143R, serum‐deprived for 48 h. Cilia length was measured with ZEN microscope tool. A mean value ± S.D. of three independent experiments is shown. In total 30 cells for each experiment were scored. Student's *t* test, **P < 0.01.ARPE‐19 cells were transiently transfected with control siRNA or siRNA targeting *PJA2* (si*PJA2*), serum‐deprived for 48 h, and immunostained for acetylated tubulin (red) and BBS1 (green). Nuclei were stained with DAPI (blue). Scale bar, 3 μm.Cumulative data of the experiments shown in (B). A mean value ± S.D. of three independent experiments is shown. In total 25 cells for each experiment were scored. Student's *t* test, **P < 0.01.ARPE‐19 cells were transiently transfected with control siRNA or siRNA targeting *PJA2* (si*PJA2*), serum‐deprived for 48 h, and immunostained for acetylated tubulin (red) and BBS2 (green). Nuclei were stained with DAPI (blue). Scale bar, 3 μm.Cumulative data of the experiments shown in (G). A mean value ± S.D. of three independent experiments is shown. In total 30 cells for each experiment were scored. Student's *t* test, **P < 0.01.ARPE‐19 cells were transiently transfected with control siRNA or siRNA targeting *PJA2* (si*PJA2*), serum‐deprived for 48 h. Cells were harvested, lysated, and immunoblotted for BBS1, BBS2, BBS4, BBS8, HSP90, and PJA2.

The ubiquitin system acts as a general control mechanism for ciliary pathways (Shiromizu *et al*, 2020). Following Shh stimulation, G‐protein‐coupled receptors (GPCRs), such as the orphan receptor GPR161, are rapidly exported out of cilium through a mechanism that requires the intact BBSome complex (Shinde *et al*, 2020). BBSome‐mediated removal of GPCRs requires ubiquitylation of receptors by a β‐arrestin‐mediated mechanism (Shinde *et al*, 2020). The data above indicate that ubiquitylation by PJA2 regulates the interaction of the BBS1 core subunit with Arl6, suggesting a possible implication of the ligase in BBSome‐mediated ciliary trafficking. To this end, we first monitored the relevance of PJA2‐dependent ubiquitylation in the ciliary localization of GPR161. ARPE‐19 cells were transiently transfected with either control or *PJA2*‐targeting siRNAs, serum‐deprived, and then immunostained for acetylated tubulin and GPR161. As shown in Fig 4A and B, while *PJA2* silencing reduced the number of ciliated cells by ~ 30%, in agreement with previous studies (Senatore *et al*, 2021), the localization of endogenous GPR161 within the cilium was not affected by PJA2 depletion. Moreover, stimulation with purmorphamine, a small agonist of the ciliary receptor Smoothened that activates the downstream hedgehog (HH) cascade, dramatically reduced the number of GPR161‐positive cilia in control (siRNAc) cells but not in *PJA2*‐silenced cells (Fig 4C and D), indicating that PJA2 plays a critical role in the ligand‐induced exit of GPR161 from cilium. These findings were also confirmed by monitoring the localization of another neuronal GPCR, somatostatin receptor 3 (SSTR3), which is typically localized within the cilium. Activation of SSTR3 by somatostatin (SST) induces a rapid exit of the receptor out of the cilium, regulating important biological functions such as neurotransmission, cell proliferation, and endocrine signaling (Nager *et al*, 2017; Shinde *et al*, 2020). To analyze the effect of PJA2 on SSTR3 trafficking, we transfected ARPE‐19 cells with control or *PJA2*‐targeting siRNAs. The cells were serum‐deprived and then stimulated for 3 h with somatostatin. As expected, hormone stimulation in controls cells induced a rapid exit of SSTR3 out of cilium, whereas downregulation of *PJA2* significantly impaired the exit of SSTR3 from cilium in somatostatin‐treated cells (Fig 4E and F). Next, we analyzed the effects of K143R mutation on the ciliary distribution of BBS1 and ciliary trafficking of GPR161. Cells were transiently transfected with either wild‐type or K143R mutant myc‐tagged BBS1 and immuno‐stained for myc and acetylated tubulin. As shown in Fig 5A, wild‐type BBS1 was mainly localized within the cilium, specifically at the basal body, transition zone, and ciliary tip. By contrast, the staining of K143R mutant was mostly excluded from the ciliary compartment and dispersed throughout the cytoplasm (Fig 5A and B). Expression of K143R mutant significantly reduced cilia length in NIH3T3 (Fig 5C) and ARPE‐19 (Fig 5D) cells. Next, we evaluated the ciliary distribution of BBS1 and BBS2 subunits in PJA2‐depleted cells. Fig 5E–H shows that the accumulation of both subunits at the base of the primary cilium was significantly reduced in cells lacking PJA2, compared with control (siRNAc) cells. Consistent with our PCA analysis, under these conditions, the bulk levels of BBSome subunits were not affected by *PJA2* deletion (Fig 5I), supporting a role of PJA2 in the recruitment and assembly of BBSome to the basal body and ciliary compartment, without altering the levels of BBS subunits.

**Figure 6:**
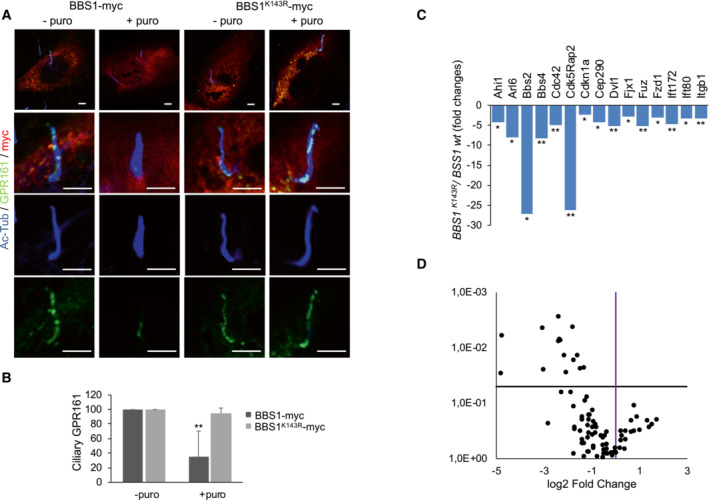
Expression of *BBS1* ^*K143R*^ affects ciliary trafficking of GPR161 and Shh signaling ARPE‐19 cells were transiently transfected with a vector for Myc‐tagged BBS1 variants, serum‐deprived for 48 h, and left untreated or stimulated with purmorphamine for 4 h. Cells were fixed and subjected to triple immunostaining analysis for GPR161 (green), acetylated tubulin (blue) and Myc (red). Scale bar, 3 μm.Cumulative data of the experiments shown in (A). A mean value ± S.D. of four independent experiments is shown. In total 25 cells for each experiment were scored. Student's *t* test, **P < 0.01.Expression of related primary cilium genes generated from Qiagen Rt^2^ profiler PCR array. Histogram shows the fold decrease of genes expressed in *PJA2* silenced ARPE‐19 cells, compared to controls. It was generated using data from 3 independent experiments, and only the triplicates considered statistically significative (*P < 0.05; **P < 0.01) were included.Volcano plot of ciliary genes obtained from Qiagen RT^2^ profiler PCR array showing the distribution of genes expression compared with the threshold.

Next, we evaluated whether the expression of BBS1 mutant interferes with the ciliary localization of GPR161. ARPE‐19 cells were transiently transfected with either wild‐type or mutant BBS1, serum‐deprived, and subjected to immunostaining analysis. Under basal conditions, expression of either wild‐type or mutant BBS1 protein had no major effects on the ciliary staining of GPR161 (Fig 6A and B). Following purmorphamine stimulation, most of the GPR161 staining was removed from cilium in BBS1wt‐expressing cells (Fig 6A and B). By contrast, in cells expressing K143R mutant, ciliary GPR161 staining was unaffected by ligand stimulation (Fig 6A and B).

Given the role of PJA2–BBS1 axis in ciliary trafficking, we addressed the functional consequences of genetic manipulation of this signaling pathway in Shh‐dependent gene transcription. To evaluate the expression of genes critical to the regulation of ciliary function, morphogenesis, and maintenance, we used a human primary cilia RT Profiler PCR Array. ARPE‐19 cells were transiently transfected with wild‐type BBS1 or with BBS1 K143R mutant. Total RNA was then extracted and analyzed by real‐time PCR using the array described above. Fig 6C and D shows that expression of BBS1 K143R markedly reduced the accumulation of mRNAs encoding for components of the SHH pathway, BBSome complex, Wnt signaling, centrosomal/pericentriolar matrix proteins, and intraciliary trafficking compared with wild‐type BBS1 expressing cells. No significant effects on cell viability were observed in cells expressing BBS1 K143R mutant, compared with BBS1wt‐expressing cells. (Appendix Fig S6).

Altogether, these findings indicate that PJA2‐mediated ubiquitylation of BBS1 is necessary for ciliary trafficking and SHH signaling.

### BBS1^K143R^ expression recapitulates the BBS phenotype in medaka fish

**Figure 7:**
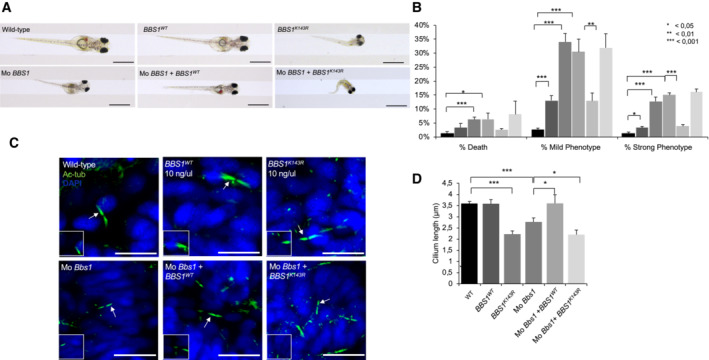
Expression of *BBS1* ^*K143R*^ affects medaka fish ciliogenesis and development Stereo‐microscopic representative images of wild‐type, wild‐type *BBS1*, *BBS* ^*K143R*^, Mo‐BBS1, Mo‐BBS1 + *BBS1* ^*WT*^, and Mo‐BBS1 + *BBS* ^*K143R*^‐injected medaka larvae, at stage 40. At least *n* = 600 embryos were injected for each condition. Scale bar, 1 mm.The graph shows the death, strong and mild phenotype percent in wild‐type and injected medaka larvae. At least *n* = 600 embryos were analyzed for each condition. The data are expressed as mean value ± SE of three independent biological replicates. Student's *t* test, *P ≤ 0.05, **P < 0.01 ***P ≤ 0.001.Confocal representative images of cilia of the neural tube cells in the wild‐type, wild‐type *BBS1*, *BBS* ^*K143R*^ (10 ng), Mo‐BBS1, Mo‐BBS1 + *BBS1* ^*WT*^, and Mo‐BBS1 + *BBS* ^*K143R*^ stained with anti‐acetylated α‐tubulin antibody (green) and DAPI (blue). Arrows indicate the cilia showed in higher magnification boxes. Scale bar, 10 μm.In the graph is reported the cilia length in wild‐type, wild‐type *BBS1*, *BBS* ^*K143R*^, Mo‐BBS1, Mo‐BBS1 + *BBS1* ^*WT*^ and Mo‐BBS1 + *BBS* ^*K143R*^. The data are expressed as mean value ± SE of eight independent experiments. Student's *t* test, *P < 0.05 ***P ≤ 0.001.

**Figure 8:**
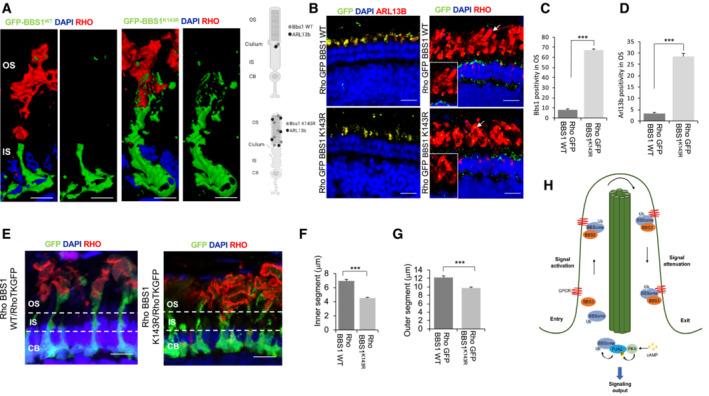
Expression of *BBS1* ^*K143R*^ alters photoreceptor IS and OS development AConfocal representative images from Airyscan superResolution show that expression of *BBS1* ^*K143R*^, but not *BBS1* ^*WT*^, translocates from IS to OS of rods from RHO:GFP:BBS1^*K143R*^ medaka transgenic line. Rods OS were immunostained for Rhodopsin. A merge composite 3D of the signals is shown. Model shows BBS1 mislocalization in rods. Scale bar, 1 μm.BConfocal representative images of co‐immunofluorescence staining of GFP and Arl13b in medaka fish photoreceptors from retinal cryosections of RHO:GFP:*BBS1* ^*WT*^ and RHO:GFP:*BBS1* ^*K143R*^ transgenic lines. *BBS1* ^*K143R*^, but not *BBS1* ^*WT*^, compromises the ARL13b localization in the photoreceptor cells. Co‐immunostaining of GFP and Rhodopsin shows an alteration of rods OS in RHO:GFP:*BBS1* ^*K143R*^ medaka transgenic line. Scale bar, 10 μm.C, DThe graphs show the quantification of BBS1‐positive and Arl13b‐positive dots in OS from RHO:GFP:*BBS1* ^*WT*^ and RHO:GFP:*BBS1* ^*K143R*^ transgenic lines. The data are expressed as mean value ± SE of five independent experiments. Student's *t* test, ***P ≤ 0.001.EConfocal representative images of co‐immunofluorescence staining of GFP and Rhodopsin in medaka fish photoreceptors. Expression of *BBS1* ^*K143R*^, but not *BBS1* ^*WT*^, alters the length of rods' IS on Rho‐TK:GFP medaka transgenic line. Scale bar, 3 μm.FThe graph shows the inner segment (μm) quantification in RHO:GFP: BBS1^WT^ and RHO:GFP:BBS1^*K143R*^ transgenic lines. The data are expressed as mean value ± SE of five independent experiments. Student's *t* test, ***P ≤ 0.001. Cell body (CB), inner segment (IS), outer segment (OS).GThe graph shows the outer segment (μm) quantification in RHO:GFP:BBS1^WT^ and RHO:GFP:BBS1^*K143R*^ transgenic lines. The data are expressed as mean value ± SE of five independent experiments. Student's *t* test, ***P ≤ 0.001.HModel of PJA2–BBSome axis. cAMP‐induced ubiquitylation of BBS1 by the E3 ligase PJA2 supports BBSome‐mediated GPCRs ciliary trafficking and signaling.

**Figure EV4:**
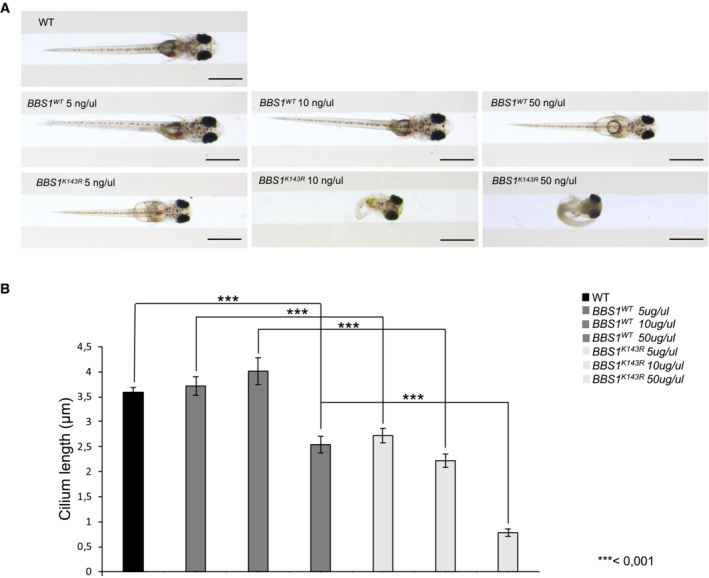
BBS1 regulates medaka fish phenotype at different concentrations Stereo‐microscopic representative images of wild‐type, wild‐type *BBS1*, and *BBS*_*K143R*_ medaka larvae, at stage 40, injected with increasing concentrations of DNA vectors (5, 10, and 50 ng/μl). At least *n* = 600 embryos were injected for each condition. Scale bar, 1 mm.In the graph is reported the cilia length in wild‐type, *BBS1* ^*WT*^ and *BBS1* ^*K143R*^ injected at 5, 10, and 50 ng/μl. The data are expressed as mean value ± SE of 10 independent experiments. Student's *t* test, ***P ≤ 0.001.

The potential pathogenicity of the K143R mutant on cilia formation and function was evaluated *in vivo* using the medaka (*Oryzias latipes*, ol) model system. To this end, we overexpressed *BBS1* ^*K143R*^ in medaka fish. Following mRNA injections, embryos injected with *BBS1* ^*K143R*^ [10 ng/μl] at the one‐cell stage were morphologically distinguishable from both *BBS1* ^*WT*^‐injected [10 ng/μl] and wild‐type control embryos from St24, corresponding to optic cup formation, onward. In particular, a large proportion (65% ± 5%) of *BBS1* ^*K143R*^‐injected exhibited short size with a slightly curved trunk, associated with a striking defect in the eye and microcephaly compared with *BBS1* ^*WT*^‐injected and untreated control embryos (Fig 7A and B). They eventually died before hatching (7% ± 2%). Importantly, our data show that these morphological alterations during embryo development significantly increased with higher *BBS1* ^*K143R*^ concentrations [from 5 to 50 ng/μl]. Specifically, the mortality of *BBS1* ^*K143R*^‐injected embryos significantly increased and the hatching rates declined, indicating a specific dose‐dependent effect compared with *BBS1* ^*WT*^‐injected and untreated control embryos (Fig EV4A and B). Thus, to assess whether the BBS1^K143R^ protein had an effect on the formation of the cilium, we analyzed cilia formation at the apical surface of cells of the neural tube at St.24–26 (2‐day postfertilization) as previously described (Senatore *et al*, 2021). Immunostaining with anti‐acetylated tubulin revealed a significant reduction in cilia length in all *BBS1* ^*K143R*^‐injected embryos compared with *BBS1* ^*WT*^‐injected and untreated control embryos (Fig 7C and D), suggesting that both embryo morphogenesis and cilia alterations are mainly a result of the *BBS1* ^*K143R*^ overexpression. These phenotypic alterations are very similar to those observed in other BBS animal models and in human BBS syndrome (Veleri *et al*, 2012; Khan *et al*, 2016; Castro‐Sanchez *et al*, 2019). To better define whether these defects were a consequence of the pathogenicity of the K143R mutant on cilia, we performed a series of rescue experiments. First, we developed an *in vivo* BBS medaka model by knocking down *olBbs1* gene, with a specific morpholino (Mo) directed against the ATG initiation codon within the 5′ untranslated region (Mo‐Bbs1). Concordantly, we observed an aberrant cilia formation associated with defects in embryo development, which culminated in evident curvature of the body axis and reduced eye size at St40, similar to those described in zebrafish (Kim *et al*, 2013). Then, the above‐described BBS1 phenotypes were significantly rescued when the Mo‐Bbs1 was co‐injected with 5 ng/μl of *BBS1* ^*WT*^ (93 ± 2% of 600 injected embryos; Fig 7A and B) but not with 5 ng/μl of the *BBS1* ^*K143R*^ (600 injected embryos; Fig 7A and B). Importantly, ciliogenesis defects and embryo phenotypes were more pronounced by the co‐injections of Mo‐Bbs1/*BBS1* ^*K143R*^, suggesting that the lack of endogenous olBbs1 exacerbated the high pathogenicity of *BBS1* ^*K143R*^ *in vivo*. We next asked whether the *BBS1* ^*K143R*^ overexpression may induce possible changes in photoreceptor structures, which result affected in the Bardet‐Biedl Syndrome. To this end, we first generated an expression vector (pSKII‐ISceI‐*hRHO:eGFP*:*BBS1* ^*K143R*^) in which the mutated form of *BBS1* was under the control of the human *RHO* (*hRHO*) promoter that drives transgene expression exclusively in rod photoreceptors (Karali *et al*, 2020). A vector (pSKII‐ISceI‐*hRHO:eGFP:BBS1* ^*WT*^) expressing wild‐type *BBS1* under the same promoter was used as a control. Consistent with *in vitro* data, the ectopic expression of *BBS1* ^*K143R*^ in differentiating rods was sufficient to mislocalize both BBS1 and olArl13b from cilia to outer segment (OS; Fig 8A–D). Importantly, these changes were associated with a reduction in OS length, suggesting an OS degeneration (Fig 8E–G). Then, by using a modified version of the expression vectors lacking the GFP (i.e., pSKII‐ISceI‐*hRHO:BBS1* ^*K143R*^ and pSKII‐ISceI‐*hRHO*:*BBS1* ^*WT*^), we overexpressed both *BBS1* ^*WT*^ and *BBS1* ^*K143R*^ in a Rho‐TK:GFP medaka transgenic line, in which GFP marks cell body, inner segments and OS of rods (Fig 8E).

Notably, ectopic expression of *BBS1* ^*K143R*^, but not *BBS1* ^*WT*^, was sufficient to induce a reduction in rod inner segment (IS) length compared with native rods, suggesting a strong reduction in cilia length (Fig 8E and F). Collectively, these observations strongly support the aberrant function of ubiquitylation‐defective BBS1^K143R^ mutant in impairing cilium formation, embryonic development, and morphogenesis of photoreceptors, thus recapitulating the BBS phenotype in the medaka fish model.

## Discussion

Here, we identify a novel regulatory mechanism essential for cilium formation and trafficking mediated by a cAMP‐induced nonproteolytic ubiquitylation of the BBSome complex by the E3 ligase PJA2. The core subunits of the BBSome, BBS1, and BBS2 are direct targets of PJA2. We report that ubiquitylation of BBS1 at the K143 regulates its binding to BBS3 and the targeting to the ciliary compartment. Loss or gain of function experiments demonstrated an essential role of PJA2–BBS1 axis in cilium formation, dynamics, and trafficking, which impacts embryo development and differentiation of the retinal photoreceptor outer segments. Inhibition of the PJA2‐BBS1 signaling pathway recapitulates most of BBS phenotypic alterations, resulting in defects of embryonic development and degeneration of retinal cells.

Ubiquitylation of components of the ciliary compartment is an essential step of cilium dynamics, and its dysregulation may contribute to the pathogenesis of human ciliopathies (Hossain & Tsang, 2019). Components of the ubiquitin system, including activating ubiquitin enzymes, E3 ubiquitin ligases, and deubiquitylases are residents at the primary cilium (Das *et al*, 2017; Massa *et al*, 2019; Toulis *et al*, 2020). Local ubiquitylation of ciliary proteins in response to extracellular stimuli modulates highly specialized ciliary activities, such as trafficking and signaling, cilium dynamics, and nuclear gene transcription (Kasahara *et al*, 2018; Desai *et al*, 2020). Recently, it was reported that ubiquitylation of ciliary GPCRs in response to SHH induction drives BBSome‐mediated exit of the receptors out of the ciliary compartment (Shinde *et al*, 2020). However, the impact of the ubiquitin signaling on the formation and regulation of the BBSome complex and its role in ciliary trafficking were unknown, so far. Our findings fill the gap and add a novel twist to the biological processes regulating ciliary trafficking by demonstrating that BBSome is a direct target of the ubiquitin signaling. We found that the RING E3 ligase PJA2 localizes at the base of the primary cilium and, in response to cAMP stimulation, ubiquitylates BBS1 and BBS2. Importantly, ubiquitylation of BBS subunits does not lead to proteolysis but most likely regulates the BBSome assembly and/or its targeting to the ciliary compartment, with important implications for cilium formation and activity. Notably, cumulating evidence is defining a scenario where the dynamic ubiquitylation represents a flexible, three‐dimensional code, essential to modulate the assembly and disassembly of the ubiquitin‐dependent protein complex. At least in this ciliary context, we show that the main role of PJA2 is to confer this ubiquitin three‐dimensional code within the BBSome. This is supported by the selective PJA2‐mediated ubiquitination of the K143 on a BBS1 domain, which is involved in the complex formation with membrane‐bound Arl6/BBS3.

In fact, ubiquitylation‐defective BBS1 mutant did not localize at the ciliary compartment, and this affected the exit of GPR161 from the cilium. These findings highlight a novel role of the ubiquitin pathway in the regulation of ciliary trafficking mediated by the BBSome. The mechanism underlying the regulation of BBS1 recruitment to membrane‐bound BBS3 is still unknown. Ubiquitin chains of the complex might favor the binding of BBS1 and/or BBSome to BBS3, thus favoring the trafficking and the sorting out of the bound complex. This is an established mechanism that cells adopt to sort ubiquitylated proteins from one intracellular compartment to another (Bonifacino & Weissman, 1998; Yu *et al*, 2019; Liu *et al*, 2020; Martinez‐Ferriz *et al*, 2021; Zajicek & Yao, 2021; Liao *et al*, 2022). Alternatively, the ubiquitin moieties may induce conformational changes and/or rearrangement in the 3D structure of the BBSome, thus promoting more stable interactions between the complex and ubiquitylated receptors. CG‐MD simulations on the hetero‐octameric *h*BBSome complex, and on its K143‐monoubiquitinated form (Ub‐*h*BBSome), do not show significant changes in the dynamic behavior of the Arl6 interacting subunits BBS1^βprop^ and BBS7^βprop^ dependent on the ubiquitin monomer. However, ubiquitination at the BBS1^4α^ K143 affects profoundly the subunits of the *h*BBSome core complex. Specifically, movements of the BBS4, BBS8, and BBS18 subunits strictly decrease when K143 is ubiquitinated in the K143‐monoubiquitin system, contributing to a higher stability of the core complex. Furthermore, most of the macroscopic movements involve BBS7^βprop^/BBS1^4α^ and BBS7. Nevertheless, these differences in protein motion did not influence the final structure of the BBSome, which, in both cases, adopts a closed conformation, compared with the Arl6‐bound open state. It is of interest to know whether the BBSome complex changes conformation when one of the subunits is polyubiquitylated. Further structural and modeling studies of ubiquitylated BBSome complexes will address this important issue.

The biological role of the BBSome ubiquitylation was investigated *in vivo* using the established medaka fish model of human ciliopathy (Veleri *et al*, 2012; Khan *et al*, 2016; Castro‐Sanchez *et al*, 2019). In this model system, introducing genetic deletion of single BBSome subunits or expressing BBS subunits carrying disease‐related germline mutations recapitulates most of the pathological phenotype of Bardet‐Biedl syndrome, such as developmental defects and photoreceptors degeneration (Khan *et al*, 2016). It is worth noting that the data shown here reproduced a BBS phenotype by expressing a specific ubiquitylation‐defective BBS1 mutant. Specifically, *BBS1* ^*K143R*^ overexpression induced major morphological alterations during fish embryo development and significant ciliary defects that closely resemble those observed in other BBS animal models (Khan *et al*, 2016; Castro‐Sanchez *et al*, 2019). Furthermore, the ectopic expression of *BBS1* ^*K143R*^ mutant in differentiating rod photoreceptors was sufficient to induce degeneration of the outer segment associated with the reduction in the length of the cilia in rod cells. In agreement with the *in vitro* data in cells, changes in ciliogenesis induced by *BBS1* ^*K143R*^ overexpression *in vivo* were associated with mislocalization of Arl13b within the ciliary compartment, further supporting the role of BBSome ubiquitylation in the control of ciliary trafficking (Fig 8H). Interestingly, similar results were obtained in zebrafish in which eye‐targeted inactivation of BBS1 gene changes OS protein and lipid composition and induces morphological OS abnormalities and retinal cell degeneration (Masek *et al*, 2022).

In summary, our data demonstrate that the nonproteolytic ubiquitylation by a ligase cAMP‐dependent (PJA2) is essential for BBSome‐mediated ciliary activities. By regulating the ubiquitylation of the core components of the BBSome, PJA2 regulates the cilium formation and local trafficking of receptors and cargoes, with major phenotypic effects on downstream signaling cascades. Derangement of this control mechanism(s) recapitulates most of the BBS phenotype(s) and cilium disorders in different species.

## Materials and Methods

### Cell lines

Human embryonic kidney cell line (HEK293) and mouse fibroblasts (NIH3T3) were cultured in Dulbecco modified Eagle's medium containing 10% fetal bovine serum (FBS) in an atmosphere of 5% CO2. Arising retinal pigment epithelial cells (ARPE‐19) were cultured with Dulbecco modified Eagle's medium/nutrient mixture F‐12 1:1 containing 10% (FBS) in an atmosphere of 5% CO_2_.

### Plasmids and transfection

Vectors encoding for HA‐BBSs were kindly provided by Dr. Val C. Sheffield, BBS1‐myc, BBS2‐myc, and BBS1 K143R‐myc were purchased from GenScript. HA‐ubiquitin, Flag‐PJA2, and PJA2 inactive mutant (Flag‐PJA2rm) carrying two‐point mutations in cys634 and cys637 to alanine was previously described (Senatore *et al*, 2021). siRNA targeting *PJA2* was purchased from Dharmacon. siRNA sequences are: 1: 5′‐GAGAUGAGUUUGAAGAGUU‐3′; sequence 2: 5′‐GGGAGAAAUUCCUUGGUUA‐3′; sequence 3: 5′‐UGACAAAGAUGAAGAUAGU‐3′; sequence 4: 5′‐UCAGAUGACCUCUUAAUAA‐3′. Control siRNA was purchased from Ambion (am4637). siRNAs were transfected using Lipofectamine 2000 (Invitrogen) at a final concentration of 100 pmol/ml of culture medium.

### Antibodies and chemicals

Primary antibodies against the following epitopes were used: flag (1:5,000 immunoblot, 1:200 immunoprecipitation; #F3165, Sigma‐Aldrich), GST (1:5,000; #sc‐138 Santa Cruz Biotechnology), PJA2 (1:1,000 immunoblot, 1:200 immunoprecipitation, 1:200 immunofluorescence; #A302‐991A, Bethyl Laboratories), GPR161 (1:100 immunofluorescence #13398‐1‐AP, Proteintech), SSTR3 (1:200 immunofluorescence #20696‐1‐AP, Proteintech), HA.11 (1:1,000; #16B12, BioLegend), mouse acetylated tubulin (1:600 immunofluorescence; #T7451, Sigma‐Aldrich), rabbit acetylated tubulin (1:600 immunofluorescence; #ab125356, Abcam), myc (1:1,000; #M4439, Sigma‐Aldrich; 1:800 immunofluorescence, Novus Biologicals #NB600‐335), ARL13B (1:500; #17711‐I‐AP, Proteintech). Antibody–antigen complexes were detected by HRP‐conjugated antibodies (Bio‐Rad Laboratories) and ECL (EuroClone). The following chemicals were used: Forskolin (40 μM; #F3917, Sigma‐Aldrich); Purmorphamine (10 μM; # ab120933 Abcam); somatostatin‐14 (10 μM; #38916–34‐6 Sigma‐Aldrich).

### Immunoprecipitation, pulldown assays, and immunoblot

Cells were lysed with buffer containing 0.5% NP40 (150 mM NaCl, 50 mM Tris–HCl pH 7.5, EDTA 1 mM, and 0.5% NP40) and supplemented with protease inhibitors, phenylmethylsulfonyl fluoride (PMSF), and phosphatase inhibitors. For ubiquitylation assays, cells were lysed with triple detergent buffer (150 mM NaCl, 1% NP40, 0.1% SDS, 50 mM Tris–HCl pH 8, 0.5% NaDOC). The lysates were incubated overnight with the indicated antibodies for immunoprecipitation. For pulldown assay, lysates were incubated 3 h with GST‐fused proteins immobilized on glutathione beads. In both cases, pellets were washed three times with lysis buffer. Filter was blocked with 5% milk in TBS Tween 0.1%, incubated with primary and secondary antibodies, and finally, proteins were detected with ECL (EuroClone). As additional experimental groups, we included serum‐deprived control cells and cells stimulated with FSK for 1 to 3 h. Then, the cells were washed with PBS and lysed with lysis buffer (10 mM sodium phosphate [pH 7.2], 150 mM NaCl, 0.5% Triton X‐100 supplemented with standard protease inhibitors and phosphatase inhibitors). After lysate clarification (13,000 rpm, 20 min) and performed IPs using protein A/G mixtures and 2 μg of control (HA antibody, Cell Signaling; myc antibody, Sigma‐Aldrich; Flag antibody, Sigma‐Aldrich) or anti‐PJA2 (Bethyl Laboratories) for 16 h at 4°C. Resin‐associated proteins were washed six times with standard lysis buffer, eluted with Laemmli sample buffer, and separated by SDS–PAGE. Immunoprecipitation and pulldown assays were previously described (Senatore *et al*, 2021).

### *In vitro* ubiquitylation assay

[35S]‐labeled BBS1 was synthesized *in vitro* using TnT quick coupled transcription/translation system (Promega, Fitchburg, WI, USA) in the presence of 45 μCi of [35S]‐labeled methionine. Ubiquitylation assay was previously described (Rinaldi *et al*, 2016).

### Fluorescence‐activated cell‐sorted (FACS) analysis

ARPE‐19 cells were transiently transfected with Myc‐tagged BBS1 variants or Myc‐tagged BBS1‐ K143R. After 24 h from transfection, cells were harvested and analyzed by FACS analysis (BD Facs CANTO II) using a fluorescein isothiocyanate (FITC) Annexin V Apoptosis Detection Kit (Annexin V‐FITC Kit; Miltenyi Bioyec Miltenyi Biotec B.V. & CO. KG Italy) and 7‐AAD staining solution (Miltenyi Bioyec Miltenyi Biotec B.V. & CO. KG Italy), according to the manufacturer's protocol.

### Immunofluorescence and confocal analysis

Cells were plated on coated glass. Cells were fixed with paraformaldehyde 4%, permeabilized with PBS 0.3% Triton, blocked with PBS 5% bovine serum albumin (SERVA), and immunostained with the indicated primary antibody. Signals were revealed using fluorescent‐ or rhodamine‐conjugated secondary antibodies (1:200; Invitrogen). Nuclei were stained with DAPI (4′,6‐Diamidino‐2‐Phenylindole, Dihydrochloride, Thermo Fisher #D1306). Immunostaining was visualized using a Zeiss LSM 700 laser scanning confocal microscope, 63×/1.4 oil immersion objective. Ciliary localization studies of GPR161, SSTR3, BBS1, and BBS2 proteins shown in Figs 4C and E, and 5A and D were scored with ZEN program of the Zeiss LSM 700 laser scanning confocal microscope. The High‐resolution images were acquired with a Zeiss LSM 880 confocal microscope equipped with an Airyscan super‐resolution imaging module, using an Å ~ 63/1.40 NA Plan‐Apochromat Oil DIC M27 objective lens (Zeiss MicroImaging, Jena, Germany). Evaluation of the Outer segment (OS) thickness of rods has been carried out by measuring the length of OS of the cells positive for anti‐Rhodopsin staining in standard areas at a comparable distance from the optic nerve. Evaluation of the Inner segment (IS) thickness of rods has been carried out by measuring the length of GFP‐positive IS up from nuclei stained with DAPI to OS stained for Rhodopsin in standard areas at a comparable distance from the optic nerve.

### Human primary cilium RT^2^ profiler PCR array

The 96‐well plate human primary cilium RT^2^ PCR assay (Cod. PAHS‐127ZR) against 84 genes belonging to primary cilia pathways, was used according to the manufacturer's instructions. Arrays were run on the Applied Biosystem Step One Plus Real‐Time PCR, using an SYBR Green qPCR Master Mix (QIAGEN). cDNA synthesis was performed using RT^2^ First Strand Kit (QIAGEN) on RNAs extracted from human ARPE transfected with BBS1‐WT and BBS1‐K143R. Quantitative RT‐PCR was performed normalizing gene expression cycle threshold (Ct), replicated three times, on the average Ct of four different control genes (B2M, GAPDH, ACTB, and GUSB). Fold change values are then presented as average fold change > 2 (average ∆Ct). Data from three independent arrays were analyzed using Qiagen RT^2^ profiler PCR array data analysis version 3.5 (http://pcrdataanalysis.sabiosciences.com/pcr/arrayanalysis.php).

### Medaka stocks

The Cab strain of wild‐type and Rho‐TK:GFP transgenic medaka (*Oryzias latipes*) lines was maintained following standard conditions (i.e., 12 h/12 h dark/light conditions at 27°C). Embryos were staged according to Beccari *et al* (2015). All studies on fish were conducted in strict accordance with the Institutional Guidelines for animal research. Ethical approval is not requested for this study that involves analyses only up the hatching of embryos because at this stage of development, they are not capable of independent feeding in accordance with the law on animal experimentation by the Italian Ministry of Health; Department of Public Health, Animal Health, Nutrition, and Food Safety (D.Lgs. 26/2014). Furthermore, all animal experiments were reviewed and approved in advance by the Ethics Committee at the TIGEM Institute, (Pozzuoli, NA), Italy.

### Morpholino and mRNA injection in medaka embryos

A morpholino (MO; Gene Tools) was designed against the ATG initiation codon within the 5′ untranslated region of the *olBBS1* gene (5′‐ CCAGCTAGCTGCAGTCTTTCACATT‐3′). The 0.09 mM diluted morpholino was injected into wild‐type medaka embryos at the one‐/two‐cell stage. The off‐target effects of the morpholino injections were excluded, and the specificity of morpholino was determined as previously described (Conte *et al*, 2010a). *In vitro* synthesis of human wild‐type *BBS1* and *BBS1* ^*K143R*^ mRNAs were performed following the manufacturer's instruction (Senatore *et al*, 2021). mRNAs were injected at 5–200 ng/μl to observe dose‐dependent phenotypes; selected working concentrations were 10 ng/μl for injections.

### Whole‐mount immunostaining

Whole‐mount immunostaining was performed and photographed, as described (Conte *et al*, 2010a). Embryos at Stage 24 were fixed in 4% paraformaldehyde, 2× phosphate‐buffered saline (PBS), and 0.1% Tween‐20. The fixed embryos were detached from the chorion and washed with PTW 1×. Embryos were digested for 7 min with 10 g/ml proteinase K and washed two‐fold with 2 mg/ml glycine/PTW 1×. The samples were fixed for 20 min in 4% paraformaldehyde, 2× phosphate‐buffered saline (PBS), and 0.1% Tween‐20, washed with PTW 1×, and then incubated for 2 h in FBS 1%/PTW 1×, at room temperature. The embryos were incubated with mouse anti‐acetylated α‐tubulin antibody 1:400 (6‐ 11B‐1; Sigma‐Aldrich, St Louis, MO, USA), overnight at 4°C. The samples were washed with PTW 1×, incubated with the secondary antibody, Alexa‐488 goat anti‐mouse IgG (ThermoFisher), then with DAPI. Finally, the embryos were placed in glycerol 100%.

### Generation of rho:GFP:BBS1 Transgenic lines

The vector bearing both BBS1 and *BBS1* ^*K143R*^ under the control of a photoreceptor‐specific promoter (pSKII‐ISceI‐Rho‐BBS1‐eGFP) was generated by inserting the Rho enhancer from pAAV.RHO.miR204 (Karali *et al*, 2020) into pSKII‐ISceI‐TK‐eGFP sequence (Beccari *et al*, 2015). Briefly, the sequence corresponding to the human RHO enhancer was released from the pAAV.RHO.miR204 plasmid by restriction with NheI and NotI and was cloned in the pSKII‐ISceI‐TK‐eGFP backbone, previously digested with the same enzymes. After this, both human wild‐type *BBS1* and *BBS1* ^*K143R*^ sequences were cloned into the pSKII‐ISceI‐Rho‐eGFP vector to create the pSKII‐ISceI‐hRHO:eGFP:*BBS1* and pSKII‐ISceI‐hRHO:eGFP:*BBS1* ^*K143R*^ constructs to drive wild‐type *BBS1* and *BBS1* ^*K143R*^ eGFP‐tagged expression in rods. Vectors were purchased from GenScript (GenScript Biotech, Netherlands). Transient transgenic fish were generated as described (Conte & Bovolenta, 2007). Transgenic expression in embryos was analyzed in living embryos under UV fluorescent stereomicroscopy (Leica Microsystems, Wetzlar, Germany).

### Immunohistochemistry on retinal cryosections

Eyes from medaka embryos were processed for immunocytochemistry as described (Conte *et al*, 2010b). Embryos were subjected to anesthesia before fixation at stage 40 by 2 h of incubation in 4% paraformaldehyde, 2× phosphate‐buffered saline (PBS), and 0.1% Tween‐20 at room temperature (RT). Samples were rinsed three times with PTW 1× (PBS 1×, 0.1% Tween‐20, pH 7.3) and then incubated overnight (ON) in 15% sucrose/PTW 1× at 4°C, and then again incubated overnight in 30% sucrose/PTW 1× at 4°C. Cryosections of the larvae were processed for immunostaining. Sections were rehydrated in PTW 1×. Blocking solution containing 10% FBS/PTW 1× was applied for 1 h at RT. Primary antibodies (GFP Invitrogen A‐6455, 1:500, Rhodopsin Thermo Fisher MA5‐11741, 1:100) were diluted in 5% FBS/PTW 1×, and sections were incubated ON at 4°C. ARL13B (1:500; #17711‐I‐AP, Proteintech). Thus, samples were washed three times with PTW 1× and incubated with secondary antibody Alexa 488 anti‐rabbit IgG (1:1,000, Invitrogen A‐11037) and Alexa 594 anti‐mouse IgG (1:1,000, Invitrogen A‐11032). Nuclei were stained with DAPI 1:500 in PBS for 10 min at RT. Finally, sections were washed three times with PTW 1× and mounted with PBS/glycerol solution.

### Homology modeling of the human hetero‐octamer BBSome ( *h*BBSome)

A wide range of practical applications of CG models has been addressed with MD simulations (Pak & Voth, 2018). Compared with the full atomistic level, in the CG description, groups of atoms are enclosed in beads, thus allowing for building a simplified representation of systems, while keeping the main chemical/physical properties. This results in the possibility to increase the orders of magnitude in the simulated time and length scales. Prior to the generation of the coarse‐grained (CG) models, the all‐atoms homology model of the human sequence BBSome (*h*BBSome) octameric complex was built. Indeed, the full octameric complex has been solved by Cryo‐EM only for the bovine sequence (*b*BBSome) in both the Arl6/GTP‐bound active (open) state (PDB ID: 6vbv; 3.5 Å) and apo (closed) inactive state (PDB ID: 6vbu; 3.1 Å; Singh *et al*, 2020). While the human sequence has been solved by Cryo‐EM only for the BBS1, BBS4, BBS8, BBS9, and BBS18 complex (PDB ID: 6xt9; 3.8 Å) and for BBS5 (PDB ID: 6xtb; 4.3 Å; Klink *et al*, 2020). The homology model of each BBS subunit was built as follows: the *h*BBS1, was built with the SwissModel webserver (Waterhouse *et al*, 2018) using as reference the *h*BBS1 of the PDB ID 6xt9; *h*BBS9 and *h*BBS18 subunits, were built with the Prime module of Maestro (Jacobson *et al*, 2004) using the PDB ID 6xt9 as reference structure; the *h*BBS5 was built with the Prime module of Maestro using the PDB ID 6xtb as reference; *h*BBS2, *h*BBS4, *h*BBS7 subunits were built with Prime using the human sequence from Uniprot webserver (Q9BXC9, Q96RK4, Q8IWZ6, respectively) and the bovine PDB ID: 6vbv as reference; *h*BBS8 was built with Prime using the PDB ID: 6xt9 as template. Finally, each *h*BBS subunit was assembled and aligned on the coordinate template of the complete hetero‐octamer bovine sequence PDB ID: 6vbv. The protonation state of each amino acid residue was calculated at physiological pH of 7.4 with the proPKA tool of the Protein Preparation Wizard module (Sastry *et al*, 2013). The monoubiquitylation at K143 located on the 4α domain of *h*BBS1 was built using the X‐ray structure of di‐ubiquitin (PDB ID: 3ns8) as template for ubiquitin (Ub) linkage and creating the isopeptide bond between C‐terminal G76 in the chain A of 3ns8 and NZ atom of K143 of *h*BBS1 with the GUI interface of Maestro. Finally, in order to avoid steric clashes, the final homology models wt‐*h*BBSome and Ub‐*h*BBSome were further refined through 5,000 steps of *in vacuo* minimization of all the protein hydrogens and heavy atoms, of which 2,500 steps with the steepest descent and 2,500 with the conjugate gradient algorithms with the AMBER18 software (Case *et al*, 2018) and the ff14SB force field (Maier *et al*, 2015). It is worth to underline that the residues numbering in both the homology models wt‐*h*BBSome and Ub‐*h*BBSome differs from that of the Cryo‐EM PDB ID: 6vbv. A corresponding comparison of the residues numbering is reported in Appendix Fig S5A–H.

### Coarse‐grained molecular dynamics

The minimized full atomistic homology models wt‐*h*BBSome and Ub‐*h*BBSome were used to build the coarse‐grained (CG) systems. CG models were created using the martinize2 tool and the latest version of the Martini forcefield (MARTINI 3) with the Elastic Network in Dynamics (ElNeDyn) approach (Periole *et al*, 2009; Telles de Souza *et al*, 2021). Standard parameters were employed for the generation of the ElNeDyn restraints (force constant 500 kJ/mol/nm^2^, maximum distance cutoff 0.9 nm). Ubiquitylated lysine was modeled as a special residue capable of establishing peptide bonds using its SC2 bead. The additional peptide bond parameters were adapted from the standard CG peptide bonds. The resulting CG structures were solvated in a cubic box with size of 25 × 25 × 25 nm using the insane tool. About 121,000 water beads were added. A 0.15 M concentration of NaCl was added to ensure electrical neutrality. Input parameters were defined as described previously (Jong *et al*, 2015), with an integration time step of 20 fs, 1.1 nm nonbonded cutoff, 1 ps time constant for temperature coupling, and 24 ps for pressure coupling. Reaction‐field and cutoff methods were employed for the treatment of electrostatic and Van der Waals interactions, respectively. V‐rescale thermostat with reference temperature 310 K and Parrinello‐Rahman isotropic barostat with reference pressure 1 bar were used (Parrinello & Rahman, 1982; Bussi *et al*, 2007). Each system was equilibrated with the backbone beads of the complex restrained for 100 ns and then simulated without restrains for 5 μs. MD simulations and analyses were performed using the GROMACS 2020 Software package (Abraham *et al*, 2015). Computation of the root mean square deviation (RMSD) and root mean square fluctuation (RMSF) was done using the gmx rmsd and gmx rmsf tools, respectively. PCA and covariance analyses were performed using the gmx covar and gmx anaeig tools. The atomic position Pearson Correlation matrix was computed using developed in‐house scripts.

## Author contributions

**Francesco Chiuso:** Investigation. **Rossella Delle Donne:** Investigation. **Giuliana Giamundo:** Investigation. **Laura Rinaldi:** Investigation. **Domenica Borzacchiello:** Investigation. **Federica Moraca:** Investigation. **Daniela Intartaglia:** Investigation. **Rosa Iannucci:** Investigation. **Emanuela Senatore:** Investigation. **Luca Lignitto:** Investigation. **Corrado Garbi:** Investigation. **Paolo Conflitti:** Investigation. **Bruno Catalanotti:** Validation; investigation; writing—original draft. **Ivan Conte:** Supervision; funding acquisition; investigation; writing—original draft; writing—review and editing. **Antonio Feliciello:** Supervision; funding acquisition; investigation; writing—original draft; writing—review and editing.

## Disclosure and competing interest statement

The authors declare that they have no conflict of interest.

## Supporting information



Appendix

Movie EV1

Movie EV2

Expanded view Figures PDF

PDF+

## Data Availability

This study includes no data deposited in external repositories.
