# Supplementary material for: Ubiquitylation of BBSome is required for ciliary assembly and signaling
Source: EMBO Rep. 2023 Feb 6;24(4):e55571. doi: 10.15252/embr.202255571 (PMC10074118; doi:10.15252/embr.202255571)
Supplement: Supplementary file 1 — Appendix [file EMBR-24-e55571-s001.pdf]

Ubiquitylation of the Bardet-Biedl syndrome protein complex is essential for cilium assembly and signaling

Francesco Chiuso et al.

Appendix Figures

Table of Contents

Appendix Figure S1 Page 2

Appendix Figure S2 Page 3

Appendix Figure S3 Page 4

Appendix Figure S4 Page 5

Appendix Figure S5 Pages 6-10

Appendix Figure S6 Page 11

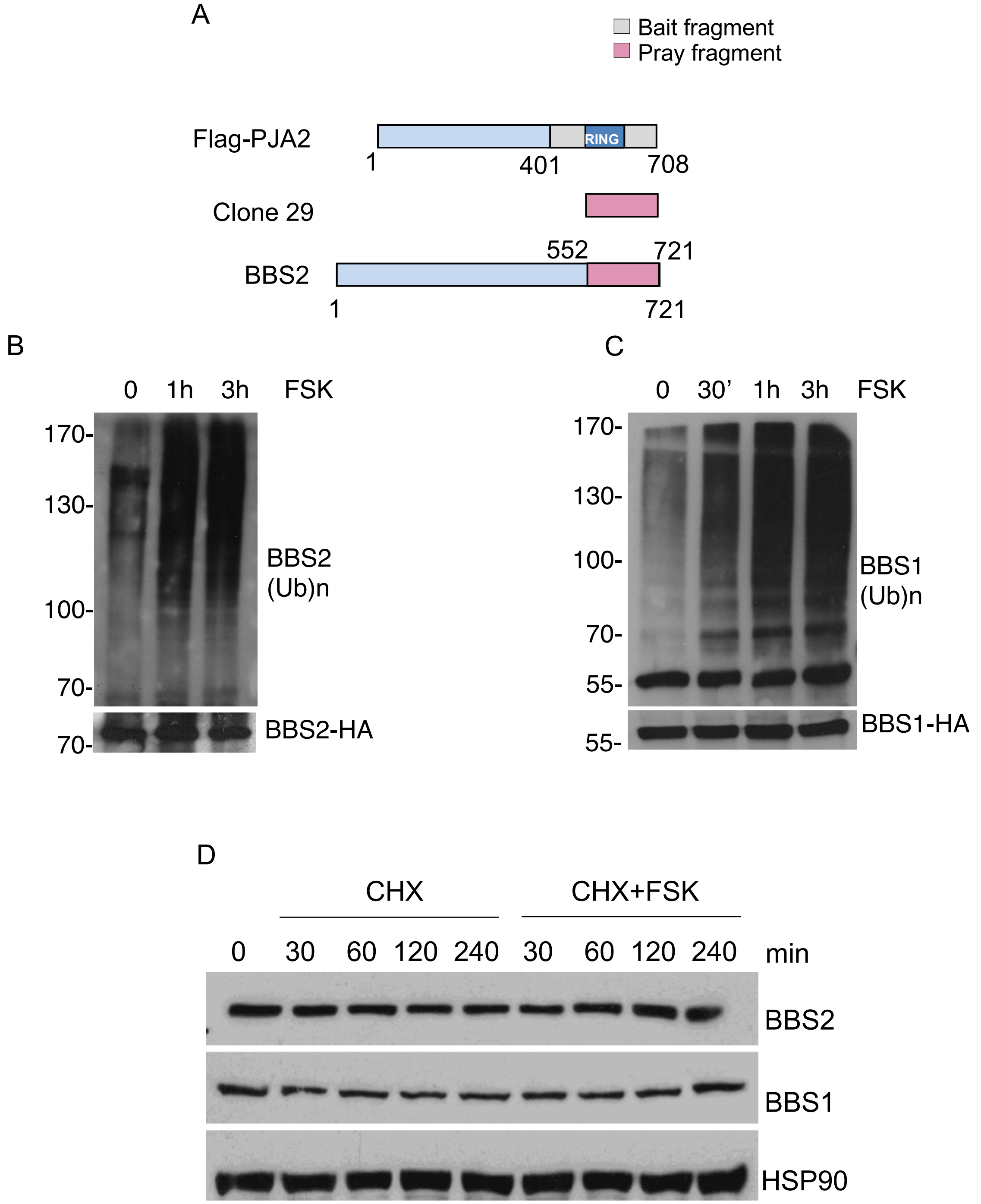

Appendix Figure S1. **A.** Schematic representation of human PJA2.The cysteine-rich region (RING) and the C-terminal rat clone 33 isolated by the yeast two-hybrid system are shown. Bait and pray fragment were indicated. **B,C** Immunoprecipitation of BBS2 (**B**) or BBS1 (**C**) from HEK293 cell lysates expressing HA-tagged BBS1 or BBS2 and ubiquitin-myc. Serum-deprived cells were left untreated or stimulated with FSK for 1 and 3 hours. Ubiquitinated BBS1 and BBS2 proteins and HA-tagged were detected. The precipitates were immunoblotted with anti-myc (ubiquitinated BBS1 or BBS2) and anti-HA antibodies. **D.** HEK293 cells were serum deprived for 24h, pretreated with cycloheximide (10μm) and stimulated with foskolin (40 μm) at indicated time. BBS1 and BBS2 protein levels were detected with endogenous antibody as indicated.

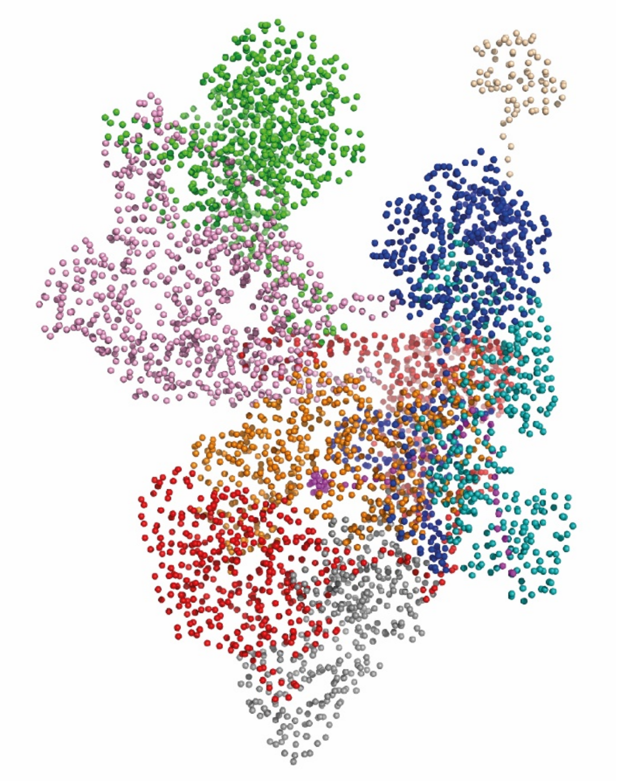

Appendix Figure S2: Coarse –Grained model of the Ub-hBBSome in an open conformation. Beads (BB) are shown as spheres.

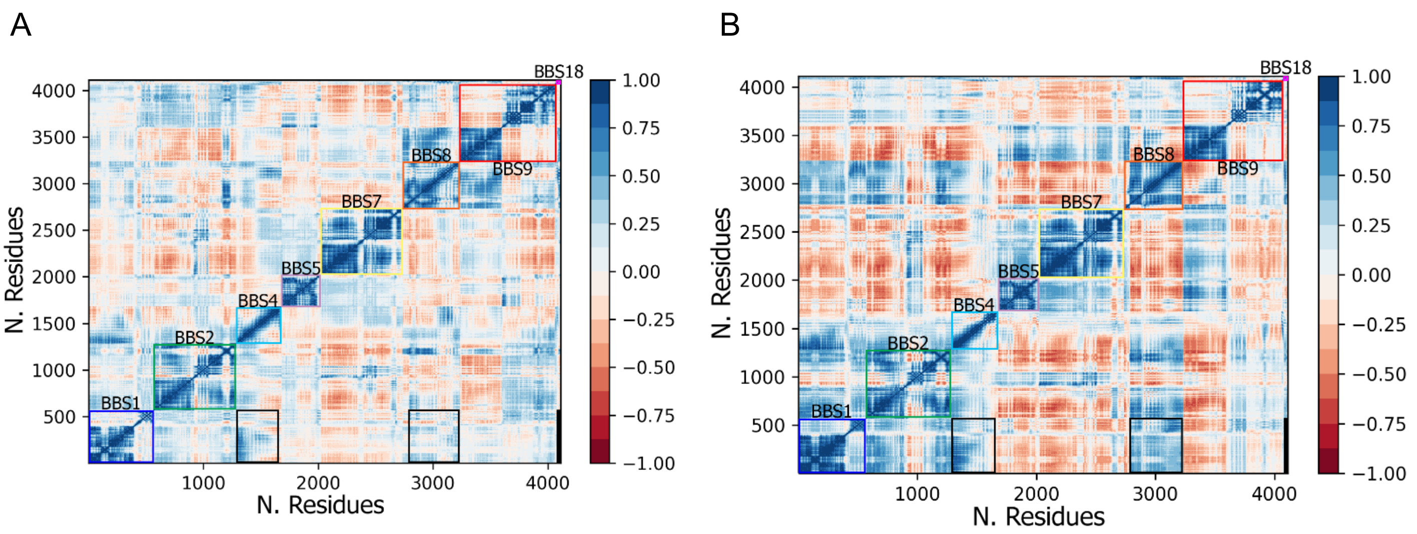

Appendix Figure S3: Atomic position Pearson Correlation Coefficient (aPCC) matrices calculated on **A** wt-hBBSome and **B** Ub-hBBSome after 5μs of CG-MD. Residues of each subunit are numbered as follows: BBS1 (S2-S568, blue rectangle),BBS2 (M570-V1285, green rectangle), BBS4 (F1288-L1683, cyan rectangle), BBS5 (M1685-S2025, light pink rectangle), BBS7 (M2026-A2740, yellow rectangle), BBS8 (M2742-F3239, orange rectangle), BBS9 (S3242-S4068, red rectangle), BBS18 (M4071-Q4122, purple rectangle). In particular, BBS18 presents a relatively high correlation (> 0.5) with BBS4 and BBS8, which is observed only in the Ub-hBBSome model. BBS18 also has high aPCC values with BBS1 and BBS2, which correlate with each other, too. BBS1 and BBS2 anticorrelate with BBS7 (aPCC < -0.5). The black rectangles highlight the zones showing high correlation between BB1, BBS4, BBS8 and BBS18 in Ub-hBBSome. The same areas are also highlighted in wt-hBBSome for the sake of comparison.

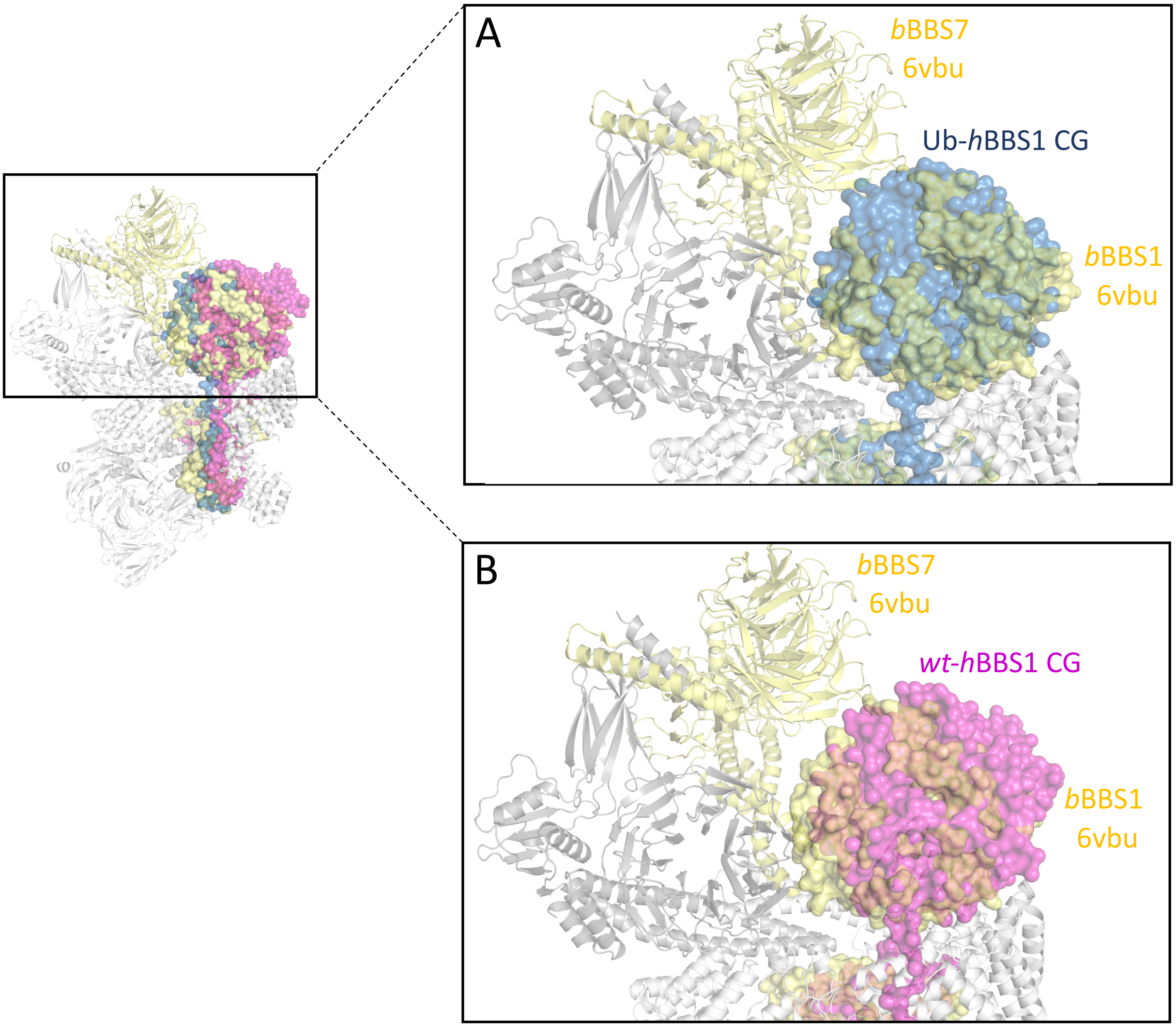

Appendix Figure S4: Superimposition between the bovine *b*BBS1 subunit of the apo (closed) form of the Cryo-EM *b*BBSome structure (PDB ID: 6vbu) (light-yellow surface) and the last CG-MD frame of: **A** CG model of the K143 Ubiquitinated *h*BBS1 (Ub-*h*BBS1 CG) (light-blue surface) and **B** CG model of the wild-type *h*BBS1 (wt-*h*BBS1 CG) (light-magenta surface). After 5 μs of CG-MD, a more closed conformation of the Ub-*h*BBS1 CG can be observed, compared to the wt-*h*BBS1 CG.

A

```
                     2                                  41
hBBS1       -------------------SKWLDAHYDPMANIHTFSACLALADLHGDGEYKLVVGDLGP
6vbu_BBS1   MAATSSSDSDGGKGESEANSKWLDSLSDSMANIHTFSACLALADFHGDGEYKLAMGDLGP
                     20                                 60

                     61                82               103
hBBS1       GGQQPRLKVLKGPLVMTESPLPALPAAAATFLMEQHEPRTPALALASGPCVYVYKNLRPY
6vbu_BBS1   DGRQPRLKVLKGHTLVSQKPLPDLPAAAVTFLMASHEPRTPALAIASGPCVYVYKNLKPY
                     80                100              121

                       124               142              162
hBBS1       FKFSLPQLPPNPLEQDLWNQAKEDRIDPLTLKEMLESIRETAEEPLSIQSLRFLQLELSE
6vbu_BBS1   FKFSLPSLPTNPLEQDLWNQAKEDQIDPLTLKEMLEGIREKAEVPLSVQSLRFLPLELSE
                       143               160              180

                     182               202              222
hBBS1       MEAFVNQHKSNSIKRQTVITTMTTLKKNLADEDAVSCLVLGTENKELLVLDPEAFTILAK
6vbu_BBS1   MEAFVNQHKSKSIRRQTVITTMTTLKKNLADEDAVSCLVLGTENKELLVLDPEAFTILAK
                     200               220              240

                     242               262              282
hBBS1       MSLPSVPVFLEVSGQFDVEFRLAAACRNGNIYILRRDSKHPKYCIELSAQPVGLIRVHKV
6vbu_BBS1   MSLPSVPAFLEASGQFDVEFRLAAACRNGSIYILRRDSKRPKYCIELGAQPVGLVGVHKV
                     260               280              300

                     302               322              342
hBBS1       LVVGSTQDSLHGFTHKGKKLWTVQMPAAILTMNLLEQHSRGLQAVMAGLANGEVRIYRDK
6vbu_BBS1   LVVGSNQDSLHGFTYKGKRLWTVQMPAAILAMNLLEQHSRGLQAVMAALANEEVRIYHDK
                     320               340              360

                     362               382              402
hBBS1       ALLNVIHTPDAVTSLCFGRYGREDNTLIMTTRGGGLIIKILKRTAVFVEGGSEVGPPPAQ
6vbu_BBS1   VLLNVIRTPEAVTSLCFGRYGREDNTLIMTTLGGGLIIKILKRTAVFAEGGGEAGPPPSQ
                     380               400              420

                     422               442              462
hBBS1       AMKLNVPRKTRLYVDQTLREREAGTAMHRAFQTDLYLLRLRAARAYLQALESSLSPLSTT
6vbu_BBS1   AIKLNVPRKTRLYVDQTLREREAGTAMHRTFQADLYLLRLRAARAYVQALESSLSPVSLT
                     440               460              480

                     482               502              522
hBBS1       AREPLKLHAVVQGLGPTFKLTLHLQNTSTTRPVLGLLVCFLYNEALYSLPRAFFKVPLLV
6vbu_BBS1   AREPLKLHAVVQGLGPTFKLTLHLQNTSTARPILGLVVCFLYNEVLYALPRAFFKVPLLV
                     500               520              540

                     542               562     568
hBBS1       PGLNYPLETFVESLSNKGISDIIKVLVLREGQSAPLLSAHVNMPGS------
6vbu_BBS1   PGLNYPLETFVKSLSDKGISDIIKVLVLREGQSTPLLSAHINMPMSEGLAAD
                     560               580           592
```

B

```
            570      589               609              629
hBBS2       MLLPVFTLKLRHKISPRMVAIGRYDGTHPCLAAATQTGKVFIHNPHTRNQHVSASRVFQS
6vbu_BBS2   MLQPVFTLKLRHKISPRMVAVGRYDGTHPCLAAATQAGKVFIHNPHSRSQHLGAPRVLQS
            1        20                40               60

                     649               669              689
hBBS2       PLESDVSLLNINQAVSCLTAGVLNPELGYDALLVGTQTNLLAYDVYNNSDLFYREVADGA
6vbu_BBS2   PLESDVSLLNINQTVSCLTAGVLNPELGYDALLVGTQTNLLAYDVYNNSDLFYREVADGA
                     80                100              120

                     709               729              749
hBBS2       NAIVLGTLGDISSPLAIIGGNCALQGFNHEGSDLFWTVTGDNVNSLALCDFDGDGKKELL
6vbu_BBS2   SAIVLGTLGDITSPLAIIGGNCALQGFNHEGNDLFWTVTGDNVHSLALCDFDGDGKKELL
                     140               160              180

                     769               789              809
hBBS2       VGSEDFDIRVFKEDEIVAEMTETEIVTSLCPMYGSRFGYALSNGTVGVYDKTSRYWRIKS
6vbu_BBS2   VGSEDFDIRVFKEDEIVAEMSETEIITSLCPMYGSRFGYALSNGTVGVYDKTARYWRIKS
                     200               220              240

                     829               849              869
hBBS2       KNHAMSIHAFDLNSDGVNELITGWSNGKVDARSDRTGEVIFKDNFSSAIAGVVEGDYRMD
6vbu_BBS2   KNQAMSIHAFDLNSDGVCELITGWSNGKVDARSDRTGEVIFKDNFSSAIAGVVEGDYRME
                     260               280              300

                     889               909              929
hBBS2       GHIQLICCSVDGEIRGYLPGTAEMRGNLMDTSAEQDLIRELSQKKQNLLLELRNYEENAK...
6vbu_BBS2   GCQQLICCSVDGEIRGYLPGTAEMRGNLMDISVEQDLIRELSQKKQNLLLELRNYEENAK...
                     320               340              360
```


Appendix Figure S5

```
                 930                 949                 969                 969
hBBS2         ...AELASPLNEADGHRGIIPANTRLHTTLSVSLGNETQTAHTELRISTSNDTIIRAVLIFAE
6vbu_BBS2     ...AELSSPLNEADGHRGVIPANTKHHTALSVSLGSEAQAAHAELCISTSNDTIIRAVLIFAE
                 361                 380                 400                 420

                                     989                 1029                1049
hBBS2            GIFTGESHVVHPSIHNLSSSICIPIVPPKDVPVDLHLKAFVGYRSSTQFHVFESTRQLPR
6vbu_BBS2        GVFAGESHVVHPSVHHLSSSVRIPITPPKDIPVDLHLKTFVGYRSSTQFHVFELTRQLPR
                                     440                 460                 480

                                     1069                1089                1109
hBBS2            FSMYALTSLDPASEPISYVNFTIAERAQRVVVWLGQNFLLPEDTHIQNAPFQVCFTSLRN
6vbu_BBS2        FSMYALTSPDPASEPLSYVNFIIAERAQRVVMWLNQNFLLPEDTNIQNAPFQVCFTSLRN
                                     500                 520                 540

                                     1129                1149                1169
hBBS2            GGHLHIKIKLSGEITINTDDIDLAGDIIQSMASFFAIEDLQVEADFPVYFEELRKVLVKV
6vbu_BBS2        GGQLYIKIKLSGEITVNTDDIDLAGDIIQSMASFFAIEDLQVEADFPVYFEELRKVLVKV
                                     560                 580                 600

                                     1189                1209                1229
hBBS2            DEYHSVHQKLSADMADHSNLIRSLLVGAEDARLMRDMKTMKSRYMELYDLNRDLLNGYKI
6vbu_BBS2        DEYHSVHQKLSADMADNSNLIRSLLVQAEDARLMRDMKTMKNRYKELYDLNKDLLNGYKI
                                     620                 640                 660

                                     1249                1269             1286
hBBS2            RCNNHTELLGNLKAVNQAIQRAGRLRVGKPKNQVITACRDAIRSNNINTLFKIMRVG----
6vbu_BBS2        RCNNHTELLGSLKAVNQAIQRAGHLRVGKPKNQVITACRDAIRSNNINMLFRIMRVGTASS
                                     680                 700              717  721
```

C

```
                                             1288                           1319
hBBS4            ----------------------------FPILEKQNWLIHLHYIRKDYEACKAVIKEQLQ
6vbu_BBS4        MAEEKLSARTQLPVSAESQKPVLKKAPEFPILEKQNWLIHLYYIQKDYEACKAVIKEQLQ
                                              29                            60

                                     1339                1359                1379
hBBS4            ETQGLCEYAIYVQALIFRLEGNIQESLELFQTCAVLSPQSADNLKQVARSLFLLGKHKAA
6vbu_BBS4        ETHGLCEYAIYVQALIFRLEGNIQESLRLFQMCAFLSPQCADNLKQVARSLFLLGKHKAA
                                      80                 100                 120

                                     1399                1419                1439
hBBS4            IEVYNEAAKLNQKDWEISHNLGVCYIYLKQFNKAQDQLHNALNLNRHDLTYIMLGKIHLL
6vbu_BBS4        IEVYNEAAKLNQKDWEICHNLGVCYIYLKQFDKAQDQLHNALHLNRHDLTYIMLGKIFLL
                                     140                 160                 180

                                     1459                1479                1499
hBBS4            EGDLDKAIEVYKKAVEFSPENTELLTTLGLLYLQLGIYQKAFEHLGNALTYDPTNYKAIL
6vbu_BBS4        KGDLDKAIEIYKKAVEFSPENTELLTTLGLLYLQLGIYQKAFEHLGNTLTYDPTNYKAIL
                                     200                 220                 240

                                     1519                1539                1559
hBBS4            AAGSMMQTHGDFDVALTKYRVVACAVPESPPLWNNIGMCFFGKKKYVAAISCLKRANYLA
6vbu_BBS4        AAGSMMQTHGDFDVALTKYKVVACAVIESPPLWNNIGMCFFGKKKYVAAISCLKRANYLA
                                     260                 280                 300

                                     1579                1599                1619
hBBS4            PFDWKILYNLGLVHLTMQQYASAFHFLSAAINFQPKMGELYMLLAVALTNLEDIENAKRA
6vbu_BBS4        PLDWKILYNLGLVHLTMQQYASAFHFLSAAINFQPKMGELYMLLAVALTNLEDSENAKRA
                                     320                 340                 360

                                     1639                1659                1679
hBBS4            YAEAVHLDKCNPLVNLNYAVLLYNQGEKKNALAQYQEMEKKVSLLKDNSSLEFDSEMVEM
6vbu_BBS4        YEEAVRLDKCNPLVNLNYAVLLYNQGEKRDALAQYQEMEKKVNLLKYSSSLEFDPEMVEV
                                     380                 400                 420

                    1683
hBBS4            AQKL--------------------------------------------------------
6vbu_BBS4        AQKLGAALQVGEALVWTKPVKDPKSKHQTASTSKAAGFQQPLGSNQALGQAMSSAATCRK
                    424

hBBS4            ---------------------------------------
6vbu_BBS4        LSSGAGGTSQLTKPPSLPLEPEPTVEAQPTEASAQTREK
```


Appendix Figure S5

D

```
                                    20                  40                  60
6VBU_5   MSVLDALWEDRDVRFDVSSQQMKTRPGEVLIDCLDSVEDTKGNNGDRGRLLVTNLRIVWH
hBBS5    MSVLDALWEDRDVRFDLSAQQMKTRPGEVLIDCLDSIEDTKGNNGDRGRLLVTNLRILWH
                                    1704                1724                1744

                                    80                  100                 120
6VBU_5   SLALPRVNLSIGYNCILNITTRTANSKLRGQTEALYVLTKCNSTRFEFIFTNLVPGSPRL
hBBS5    SLALSRVNVSVGYNCILNITTRTANSKLRGQTEALYILTKCNSTRFEFIFTNLVPGSPRL
                                    1764                1784                1804

                                    140                 160                 180
6VBU_5   YTSLIAVHRAYETSKMYRDFKLRSALIQNKQLRLLPQENVYNKINGVWNLSSDQGNLGTF
hBBS5    FTSVMAVHRAYETSKMYRDFKLRSALIQNKQLRLLPQEHVYDKINGVWNLSSDQGNLGTF
                                    1824                1844                1864

                                    200                 220                 240
6VBU_5   FITNVRIVWHANMNDSFNVSIPYLQIRSVKIRDSKFGLALVIESSQQSGGYVLGFKIDPV
hBBS5    FITNVRIVWHANMNDSFNVSIPYLQIRSIKIRDSKFGLALVIESSQQSGGYVLGFKIDPV
                                    1884                1904                1924

                                    260                 280                 300
6VBU_5   EKLQESVKEINSLHKVYSANPIFGVDYEMEEKPQPLEALTVKQIQDDVEIDSDDHTDAFV
hBBS5    EKLQESVKEINSLHKVYSASPIFGVD----------------------------YTDAFV
                                    1944                                    1984

                                    320                 341
6VBU_5   AYFADGNKQQDREPVFSEELGLAIEKLKDGFTLQGLWEVMN
hBBS5    AYFADGNKQQDREPVFSEELGLAIEKLKDGFTLQGLWEVS-
                                    2004                2025
```

E

```
                                    20                  40                  60
6VBU_7   MDLNLNRADYLQVGVTSQKTMKLLPASKHRATQKVVVGDHDGIVMCFGMKKGEAVTVFKT
hBBS7    MDLILNRMDYLQVGVTSQKTMKLIPASRHRATQKVVIGDHDGVVMCFGMKKGEAAAVFKT
                                    2045                2065                2085

                                    80                  100                 120
6VBU_7   LPGQKIARLELGGALNTPQEKIFIAAGSEIRGFTKRGKQFLSFETNLTESIKAMHISGSD
hBBS7    LPGPKIARLELGGVINTPQEKIFIAAASEIRGFTKRGKQFLSFETNLTESIKAMHISGSD
                                    2105                2125                2145

                                    140                 160                 180
6VBU_7   LFLSASYIYNHYCDCKDQHYYLSGDKINDVICLPVERLLREVPVLACQDRVLRVLQGSDV
hBBS7    LFLSASYIYNHYCDCKDQHYYLSGDKINDVICLPVERLSRITPVLACQDRVLRVLQGSDV
                                    2165                2185                2205

                                    200                 220                 240
6VBU_7   TYEIEVPGPPTVLALHNGNGGDSGEDLLFGTSDGKLGLIQITTSKPIHKWEIRNEKKRGG
hBBS7    MYAVEVPGPPTVLALHNGNGGDSGEDLLFGTSDGKLALIQITTSKPVRKWEIQNEKKRGG
                                    2225                2245                2265

                                    260                 280                 300
6VBU_7   ILCVDSFDIVGDGVKDLLVGRDDGMVEVYGFDNANEPVLRFDHTLSESVTSIQGGCVGKD
hBBS7    ILCIDSFDIVGDGVKDLLVGRDDGMVEVYSFDNANEPVLRFDQMLSESVTSIQGGCVGKD
                                    2285                2305                2325

                                    320                 340                 360
6VBU_7   GYDEIVVSTYSGWITGLTTEPVHKESGPGEELKFNQEMQNKISSLRSELEQLQYKVLQER
hBBS7    SYDEIVVSTYSGWVTGLTTEPIHKESGPGEELKINQEMQNKISSLRNELEHLQYKVLQER
                                    2345                2365                2385

                                    380                 400                 420
6VBU_7   EKYQQSSQSSKAKSAVPSFSVNDKFTLNKDDASYSLILEVQTAIDNVLIQSDVPIDLLDV
hBBS7    ENYQQSSQSSKAKSAVPSFGINDKFTLNKDDASYSLILEVQTAIDNVLIQSDVPIDLLDV
                                    2405                2425                2445

                                    440                 460                 480
6VBU_7   DKNSAVVSFSSCDSESNDNFLLATYRCQANTTRLELKIRSIEGQYGTLQAYVTPRIQPKT
hBBS7    DKNSAVVSFSSCDSESNDNFLLATYRCQADTTRLELKIRSIEGQYGTLQAYVTPRIQPKT
                                    2465                2485                2505

                                    500                 520                 540
6VBU_7   CQVRQYHIKPLSLHQRTHFIDHDRPMNTLTLTGQFSFSELHSWVVFCMPEVPEKPPAGEC
hBBS7    CQVRQYHIKPLSLHQRTHFIDHDRPMNTLTLTGQFSFAEVHSWVVFCLPEVPEKPPAGEC
                                    2525                2545                2565

                                    560                 580                 600
6VBU_7   VTFYFQNTFLDTQLESTYRKGEGVFKSDNISTISILKDVLSKEATKRKINLNISYEINEV
hBBS7    VTFYFQNTFLDTQLESTYRKGEGVFKSDNISTISILKDVLSKEATKRKINLNISYEINEV
                                    2585                2605                2625
```


Appendix Figure S5

```
                                     620                 640                 660
6VBU_7      SVKHTLKLIHPKLEYQLLLAKKVQLIDALKELQVHEGNTNFLIPEYRCILEEADHLQEEY
hBBS7       SVKHTLKLIHPKLEYQLLLAKKVQLIDALKELQIHEGNTNFLIPEYHCILEEADHLQEEY
                                     2645                2665                2685

                                     680                 700            715
6VBU_7      KKQPAHLERLYGMITDLFIDKFKFKGTNVKTKVPLLLEILDSYDQNALIAFFDAA
hBBS7       KKQPAHLERLYGMITDLFIDKFKFKGTNVKTKVPLLLEILDSYDQNALISFFDAA
                                     2705                2725           2740
```

F

```
                                 20                  40                  60
6VBU_BBS8   MEPLLLAWSYFRRRRFQLCADLCTQMLEKSPCDQAAWILKARALTEMVYVDEIDVDEEGI
hBBS8       MEPLLLAWSYFRRRKFQLCADLCTQMLEKSPYDQAAWILKARALTEMVYIDEIDVDQEGI
                                 2760                2780                2800

                                 80                  100                 120
6VBU_BBS8   AEMILDENAIAQVPRPGTSLKLPGTNQTGGPSPAVRPVTQAGRPITGFLRPSTQSGRPGT
hBBS8       AEMMLDENAIAQVPRPGTSLKLPGTNQTGGPSQAVRPITQAGRPITGFLRPSTQSGRPGT
                                 2820                2840                2860

                                 140                 160                 180
6VBU_BBS8   IEQAIKTPRTAYTARPIASSSGRFVRLGTASMLTSPDGPFINLSRLNLAKYAQKPKLAKA
hBBS8       MEQAIRTPRTAYTARPITSSSGRFVRLGTASMLTSPDGPFINLSRLNLTKYSQKPKLAKA
                                 2880                2900                2920

                                 200                 220                 240
6VBU_BBS8   LFEYIFHHENDVKTALDLAALSTEHSQYKDWWWKVQIGKCYYRLGLYREAEKQFKSALKQ
hBBS8       LFEYIFHHENDVKTALDLAALSTEHSQYKDWWWKVQIGKCYYRLGMYREAEKQFKSALKQ
                                 2940                2960                2980

                                 260                 280                 300
6VBU_BBS8   QEMVDTFLYLAKVYISLDQPLTALNLFKQGLDKFPGEVTLLCGIARIYEEMNNISSATEY
hBBS8       QEMVDTFLYLAKVYVSLDQPVTALNLFKQGLDKFPGEVTLLCGIARIYEEMNNMSSAAEY
                                 3000                3020                3040

                                 320                 340                 360
6VBU_BBS8   YKEVLKQDNTHVEAIACIGSNHFYTDQPEVALRFYRRLLQMGVYNCQLFNNLGLCCFYAQ
hBBS8       YKEVLKQDNTHVEAIACIGSNHFYSDQPEIALRFYRRLLQMGIYNGQLFNNLGLCCFYAQ
                                 3060                3080                3100

                                 380                 400                 420
6VBU_BBS8   QYDMTLTSFERALSLAENEEEVADVWYNLGHVAVGTGDTNLAHQCFRLALVSNNQHAEAY
hBBS8       QYDMTLTSFERALSLAENEEEAADVWYNLGHVAVGIGDTNLAHQCFRLALVNNNNHAEAY
                                 3120                3140                3160

                                 440                 460                 480
6VBU_BBS8   NNLAVLEMRRGHVEQAKALLQTASSLAPHMYEPHFNFATISDKIGDLQRSYAAAKKSEAA
hBBS8       NNLAVLEMRKGHVEQARALLQTASSLAPHMYEPHFNFATISDKIGDLQRSYVAAQKSEAA
                                 3180                3200                3220

                                 500
6VBU_BBS8   FPDHVDTQHLIKQLEQHFAML
hBBS8       FPDHVDTQHLIKQLRQHFA--
                                 3240
```

G

```
                                 20                  40                  60
6VBU_BBS9   MSLFKARDWWSTVLGDKEEFDQGCLCLADVDNTGNGQDKIIVGSFMGYLRIFNPHPVKTG
hBBS9       -SLFKARDWWSTILGDKEEFDQGCLCLANVDNSGNGQDKIIVGSFMGYLRIFSPHPAKTG
                                 3260                3280                3300

                                 80                  100                 120
6VBU_BBS9   DGAQAEDLLLEVHLRDPILQVEVGKFVSGTEMLHLAVLHSRKLCVYSVSGTLGNVEHGNQ
hBBS9       DGAQAEDLLLEVDLRDPVLQVEVGKFVSGTEMLHLAVLHSRKLCVYSVSGTLGNVEHGNQ
                                 3320                3340                3360

                                 140                 160                 180
6VBU_BBS9   YQIKLMYEHNLQRTACNMTYGSFGGVKGRDLICIQSVDGMLMVFEQESYAFGRFLPGSLL
hBBS9       CQMKLMYEHNLQRTACNMTYGSFGGVKGRDLICIQSMDGMLMVFEQESYAFGRFLPGFLL
                                 3380                3400                3420

                                 200                 220                 240
6VBU_BBS9   PGPLAYSSRTDSFITVSSCHQVESYKYQVLAFATDADKRQETEQQKHGSGKRLVVDWTLN
hBBS9       PGPLAYSSRTDSFLTVSSCQQVESYKYQVLAFATDADKRQETEQQKLGSGKRLVVDWTLN
                                 3440                3460                3480

                                 260                 280                 300
6VBU_BBS9   IGEQAIDICIVSFIQSASSVFVLGERNFFCLKDNGQIQFMKKLDYSPSCFLPYCSVSEGT
hBBS9       IGEQALDICIVSFNQSASSVFVLGERNFFCLKDNGQIRFMKKLDWSPSCFLPYCSVSEGT
                                 3500                3520                3540

                                 320                 340                 360
6VBU_BBS9   INTLIGNHNNMLHIYQDVTLKWATQLPHVPVAVRVGCLHDLKGVIVTLSDDGHLQCSYLG
hBBS9       INTLIGNHNNMLHIYQDVTLKWATQLPHIPVAVRVGCLHDLKGVIVTLSDDGHLQCSYLG
                                 3560                3580                3600
```


Appendix Figure S5

```
                                   380                         400                        420
6VBU_BBS9     TDPSLFQAPKVESRELNYDELDMELKELQKVIKNVNKSQDVWPLTEREDDLKVSAMVSPN
hBBS9         TDPSLFQAPNVQSRELNYDELDVEMKELQKIIKDVNKSQGVWPMTEREDDLNVSVVVSPN
                                  3620                        3640                       3660

                                   440                         460                        480
6VBU_BBS9     FDSVSQATDVEVGADLVPSVTVKVTLKNRVALQKIKLSIYVQPPLVLTGDQFTFEFMAPE
hBBS9         FDSVSQATDVEVGTDLVPSVTVKVTLQNRVILQKAKLSVYVQPPLELTCDQFTFEFMTPD
                                  3680                        3700                       3720

                                   500                         520                        540
6VBU_BBS9     MTRTVGFSVYLKGSYSPPELEGNAVVSYSRPTERNPDGIPRVSQCKFRLPLKLVCLPGQP
hBBS9         LTRTVSFSVYLKRSYTPSELEGNAVVSYSRPTDRNPDGIPRVIQCKFRLPLKLICLPGQP
                                  3740                        3760                       3780

                                   560                         580                        600
6VBU_BBS9     SKTASHKLTIDTNKSPVSLLSLFPGFAKQSEDDQVNVMGFRFLGGSQVTLLASKTSQRYR
hBBS9         SKTASHKITIDTNKSPVSLLSLFPGFASQSDDDQVNVMGFHFLGGARITVLASKTSQRYR
                                  3800                        3820                       3840

                                   620                         640                        660
6VBU_BBS9     IQSEQFEDLWLITNELIIRLQEYFEKQGIKDFTCSFSGSVPLEEYFELIDHHFELRINGE
hBBS9         IQSEQFEDLWLITNELILRLQEYFEKQGVKDFACSFSGSIPLQEYFELIDHHFELRINGE
                                  3860                        3880                       3900

                                   680                         700                        720
6VBU_BBS9     KLEELLSERAVQFRAIQRRLLTRFKDKTPAPLQHLDTLLDGTYKQVIALADAVEENQDNL
hBBS9         KLEELLSERAVQFRAIQRRLLARFKDKTPAPLQHLDTLLDGTYKQVIALADAVEENQGNL
                                  3920                        3940                       3960

                                   740                         760                        780
6VBU_BBS9     FQSFTRLKSATHLVILLIGLWQKLSADQIAILEAAFLPLQQDTQELGWEETVDAALSHLL
hBBS9         FQSFTRLKSATHLVILLIALWQKLSADQVAILEAAFLPLQEDTQELGWEETVDAAISHLL
                                  3980                        4000                       4020

                                   800                         820          828
6VBU_BBS9     KTCLSKSSKEQALNLNSQLGIPKDTSQLKKHITLFCDRLAKGGRLCLSTDAAAPQTMVMP
hBBS9         KTCLSKSSKEQALNLNSQLNIPKDTSQLKKHITLLCDRLSKGGRLCLS------------
                                  4040                        4060         4068

6VBU_BBS9     GGCATIPESDLEGRSIDQDSSELFTNHKHLMVETPVPEVSPLQGVTE
hBBS9         -----------------------------------------------
```

H

```
                                   20                          40                        58
6VBU_BBS18    MAETKSMFREVLPKQGQLYVEDITTMVLCKPKLLPLKSLTLEKLEKMQQAAQDTIHQQEM
hBBS18        ------MFREVLPKQGPLFVEDIMTMVLCKPKLLPLKSLTLEKLEKMHQAAQNTIRQQ--
                                 4084                        4104                       4122

6VBU_BBS18    TEKEQKITH
hBBS18        ---------
```

Appendix Figure S5: BBSome bovine and human sequence alignments, highlighting the different residue numbers between the experimental (PDB ID: 6vbu) and the human homology model used during CG-MD simulations. **A.** *b*BBS1/*h*BBS1; **B**. *b*BBS2/*h*BBS2; **C**. *b*BBS4/*h*BBS4; **D**. *b*BBS5/*h*BBS5; **E**. *b*BBS7/*h*BBS7; **F**. *b*BBS8/*h*BBS8; **G**. *b*BBS9/*h*BBS9; **H**. *b*BBS18/*h*BBS18.

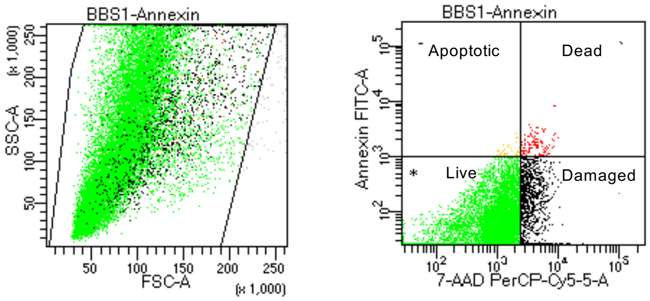

Tube: Annexin

Population	#Events	%Parent	%Total
All Events	20,000	####	100.0
' Cells	19,430	97.2	97.2
- Apoptotic	38	0.2	0.2
` Dead	109	0.6	0.5
* Live	18,102	93.2	90.5
; Damaged	1,181	6.1	5.9

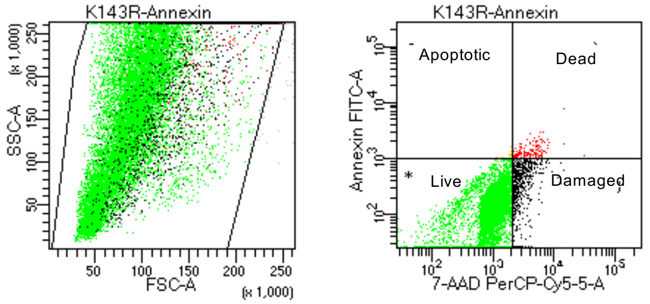

Tube: Annexin

Population	#Events	%Parent	%Total
All Events	20,000	####	100.0
' Cells	19,205	96.0	96.0
- Apoptoti	19	0.1	0.1
` Dead	256	1.3	1.3
* Live	17,714	92.2	88.6
; Damaged	1,216	6.3	6.1

Appendix Figure S6: ARPE-19 cells were transiently transfected with Myc-tagged BBS1 variants or Myc-tagged BBS1- K143R. After 24h from transfection, cells were harvested and analyzed using a fluorescein isothiocynate (FITC) Annexin V Apoptosis Detection Kit and 7-AAD staining solution, according to the manufacturer's protocol.
