# Supplementary material for: Ubiquitylation of BBSome is required for ciliary assembly and signaling
Source: EMBO Rep. 2023 Feb 6;24(4):e55571. doi: 10.15252/embr.202255571 (PMC10074118; doi:10.15252/embr.202255571)
Supplement: Supplementary file 2 — Movie EV1 [file EMBR-24-e55571-s004.zip › Legend MOVIE EV1.docx]

**Legend MOVIE EV1**

**MOVIE EV1:** Movie showing the first eigenvector (EV1) of the wt-*h*BBSome and the macroscopic movements of BBS1 (blue spheres) towards BBS7 (green spheres) rotating along the axis defined by the heterodimerization helix domain.
