# Supplementary material for: Ubiquitylation of BBSome is required for ciliary assembly and signaling
Source: EMBO Rep. 2023 Feb 6;24(4):e55571. doi: 10.15252/embr.202255571 (PMC10074118; doi:10.15252/embr.202255571)
Supplement: Supplementary file 3 — Movie EV2 [file EMBR-24-e55571-s003.zip › Legend MOVIE EV2.docx]

**Legend MOVIE EV2**

**MOVIE EV2 :** Movie showing the first eigenvector (EV1) of the Ub-*h*BBSome and the macroscopic anti-clockwise rotation of BBS1^βprop^ and BBS1^4α^ domains (blue spheres) with respect to BBS7^βprop^ (green spheres).
