# Supplementary material for: Ubiquitylation of BBSome is required for ciliary assembly and signaling
Source: EMBO Rep. 2023 Feb 6;24(4):e55571. doi: 10.15252/embr.202255571 (PMC10074118; doi:10.15252/embr.202255571)
Supplement: Supplementary file 4 — Expanded view Figures PDF [file EMBR-24-e55571-s005.pdf]

Expanded View Figures

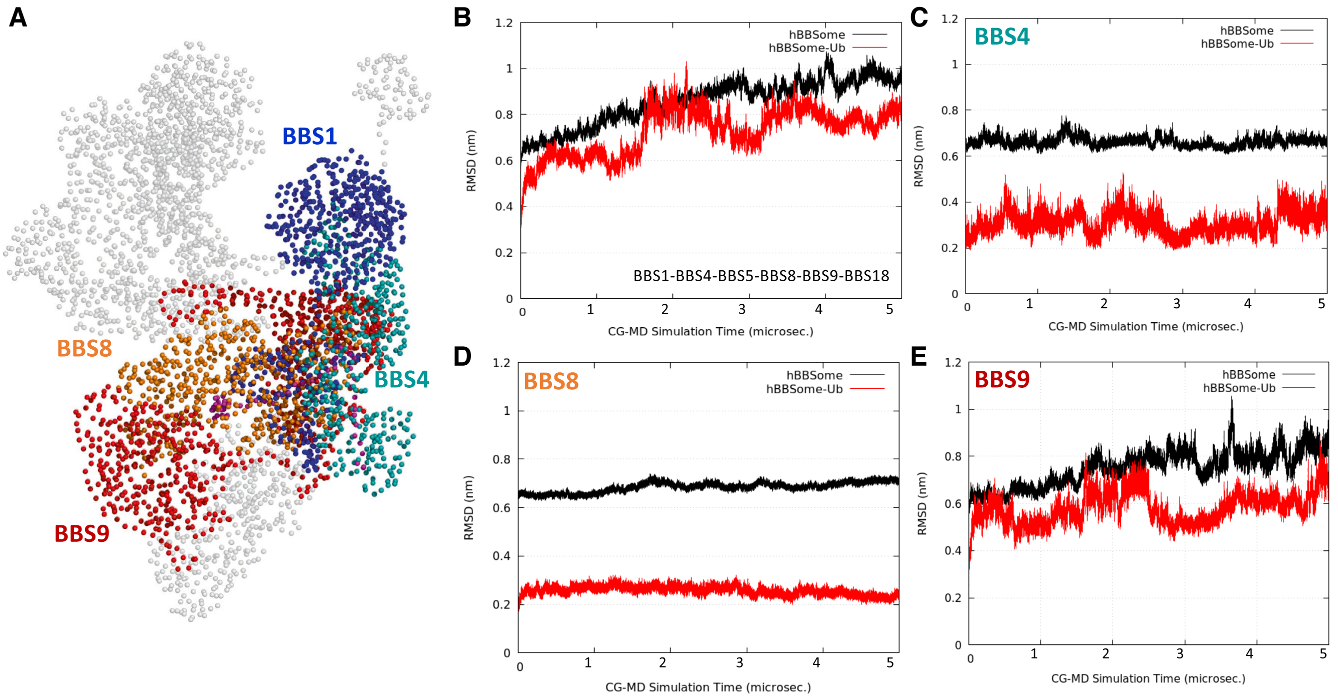

Figure EV1. Modeling of the Ub-*h*BBSome core complex.

A CG model highlighting the Ub-*h*BBSome core complex BBS1, BBS4, BBS8, and BBS9 (CG beads are represented as spheres).

B Plot of the RMSD trend of the *h*BBSome core complex (black lines) and the Ub-*h*BBSome core complex (red lines).

C–E Plot of the RMSD trend of the single *h*BBSome core complex subunits, BBS4, BBS5, BBS8, and BBS9, respectively. Each plot shows the higher stabilization of each subunit of the core complex in the monoubiquitinated CG model (red lines), with respect to the *h*BBSome wild-type (black lines). RMSD calculations were performed on the CG-MD production run considering only the BBSome beads (represented as spheres in panel A).

 © 2023 The Authors

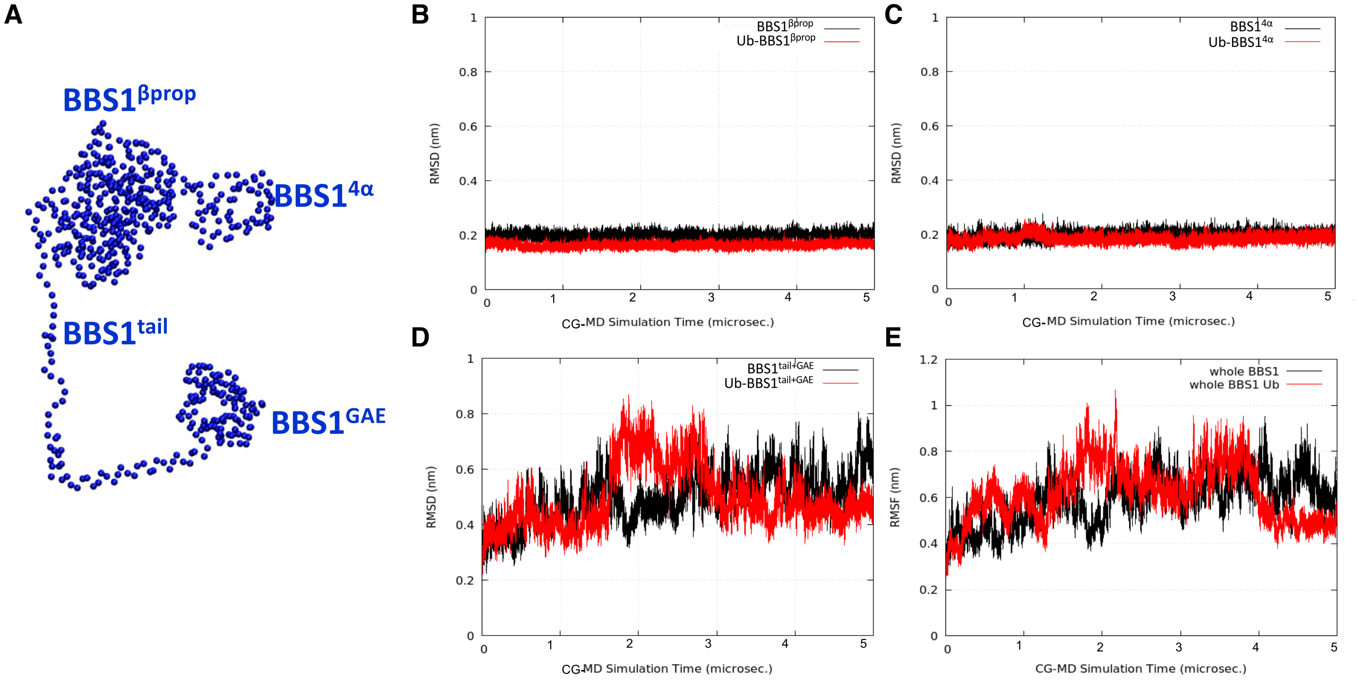

Figure EV2. Dynamic modeling of BBS1 domains.

A CG beads of the BBS1 domains β-propeller, 4α, tail, and GAE.

B–D Plot of the RMSD trend of the BBS1 β-propeller, 4α and tail+GAE domains, respectively. It can be observed that the main movements in the BBS1 subunit concern the domain formed by tail and GAE (d), while both β-propeller and 4α domains are very stable along 5 μs of CG-MD (b and c) in both the K143 ubiquitinated and wild-type systems.

E Plot of the RMSD trend of the entire BBS1. RMSD calculations were performed on the CG-MD production run considering only the BBS1 beads (represented as spheres in panel A).

© 2023 The Authors

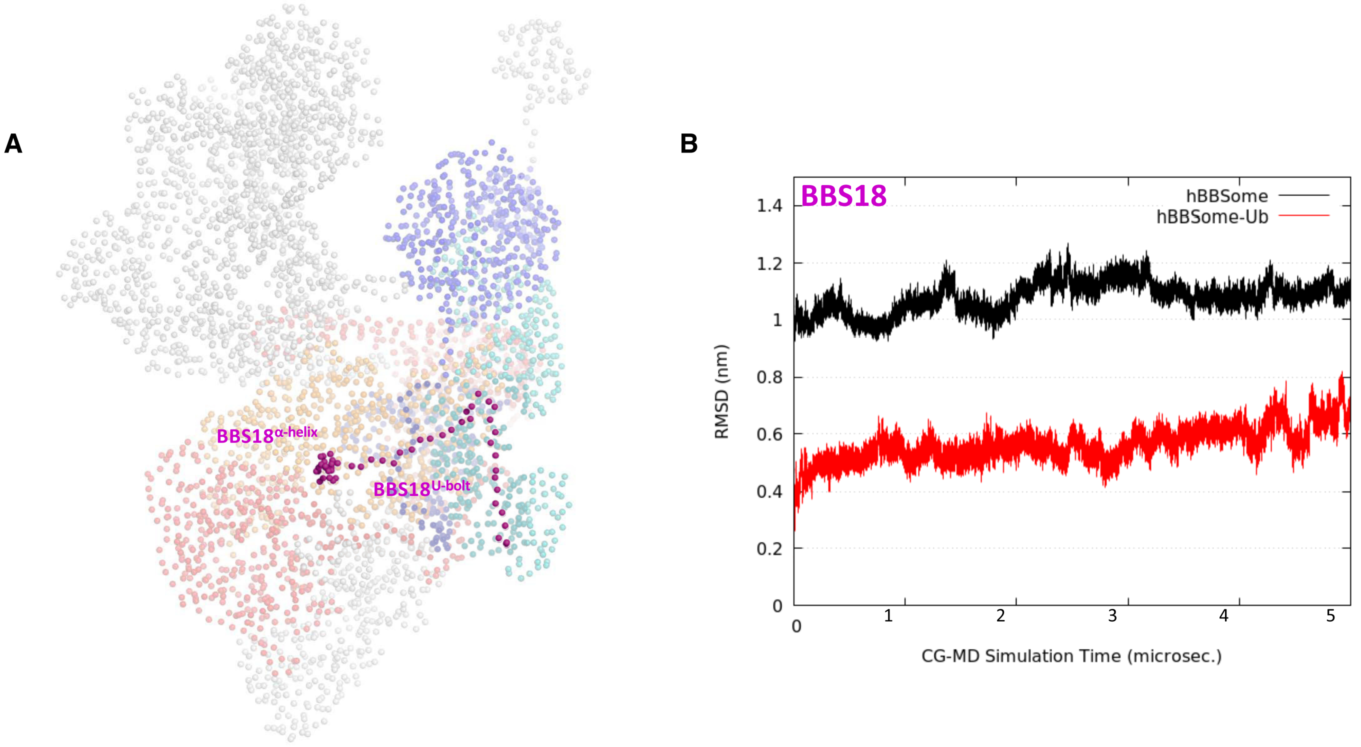

Figure EV3. Dynamic modeling of Ub-*h*BBSome complex.

A CG beads of the Ub-*h*BBSome model, highlighting the BBS18 subunit (magenta spheres).

B Root Mean Square Deviation (RMSD) of BBS18 subunit, highlighting the reduced mobility of *h*BBS18 residues in the K143 monoubiquitinated *h*BBSome (Ub-*h*BBSome; red lines). RMSD calculations were performed on the CG-MD production run considering only the BBSome beads (represented as spheres in panel A).

 © 2023 The Authors

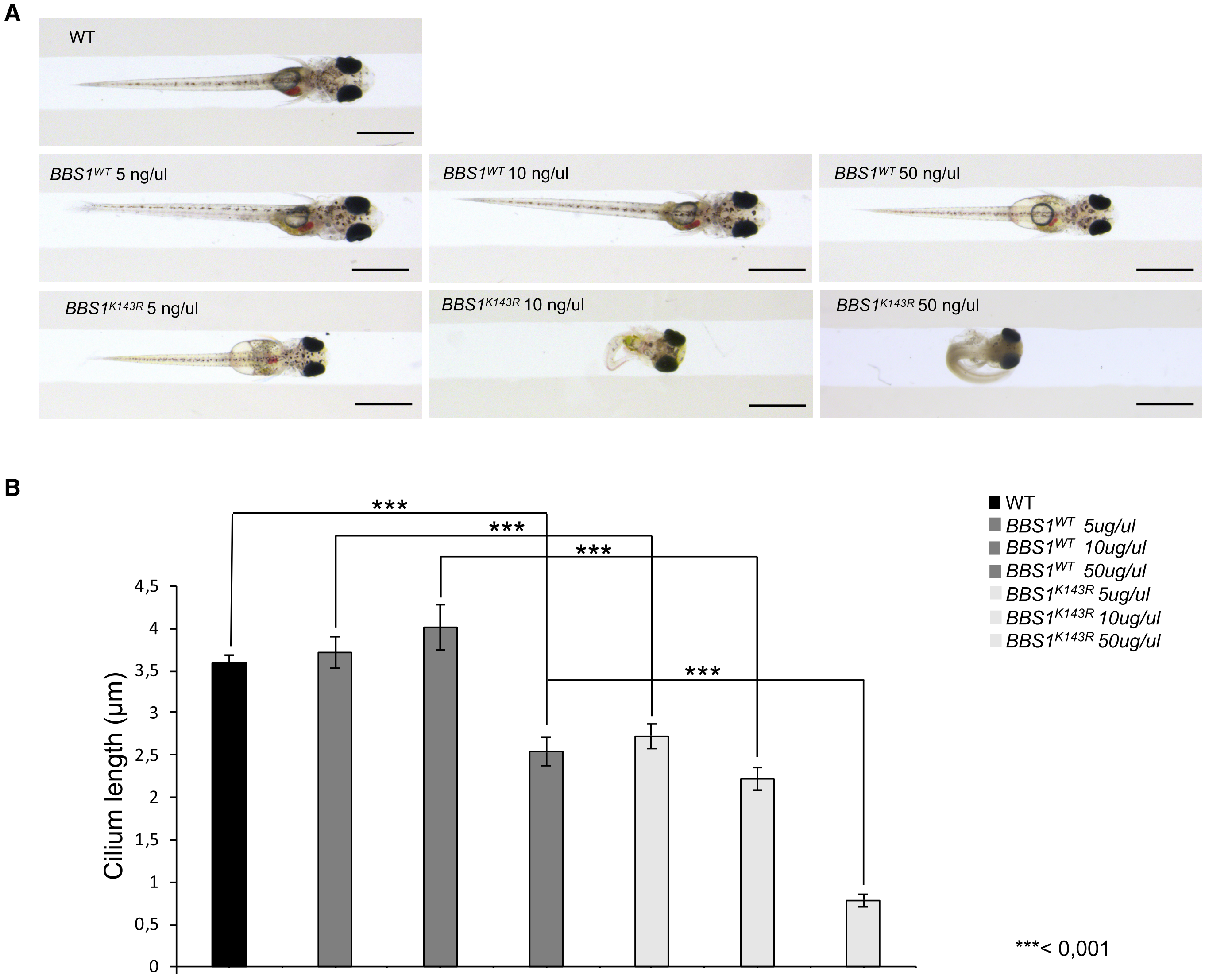

Figure EV4. BBS1 regulates medaka fish phenotype at different concentrations.

A Stereo-microscopic representative images of wild-type, wild-type *BBS1*, and BBS_{K143R} medaka larvae, at stage 40, injected with increasing concentrations of DNA vectors (5, 10, and 50 ng/µl). At least $n = 600$ embryos were injected for each condition. Scale bar, 1 mm.

B In the graph is reported the cilia length in wild-type, $BBS1^{WT}$ and $BBS1^{K143R}$ injected at 5, 10, and 50 ng/µl. The data are expressed as mean value ± SE of 10 independent experiments. Student's *t* test, ***$P \leq 0.001$.
