# Supplementary material for: Ubiquitylation of BBSome is required for ciliary assembly and signaling
Source: EMBO Rep. 2023 Feb 6;24(4):e55571. doi: 10.15252/embr.202255571 (PMC10074118; doi:10.15252/embr.202255571)
Supplement: Supplementary file 6 [file EMBR-24-e55571-s002.pdf]

EMBO reports

Ubiquitylation of BBSome is required for ciliary assembly and signaling

Francesco Chiuso, Rossella Delle Donne, Giuliana Giamundo, Laura Rinaldi, Domenica Borzacchiello, Federica Moraca, Daniela Intartaglia, Rosa Iannucci, Emanuela Senatore, Luca Lignitto, Corrado Garbi, Paolo Conflitti, Bruno Catalanotti, Ivan Conte, and Antonio Feliciello
DOI: 10.15252/embr.202255571

Corresponding author(s): Ivan Conte (conte@tigem.it) , Antonio Feliciello (feliciel@unina.it)

Review Timeline:

Submission Date:	10th Jun 22
Editorial Decision:	14th Jul 22
Revision Received:	13th Oct 22
Editorial Decision:	16th Dec 22
Revision Received:	27th Dec 22
Accepted:	17th Jan 23

Editor: Martina Rembold

Transaction Report:

(Note: With the exception of the correction of typographical or spelling errors that could be a source of ambiguity, letters and reports are not edited. Depending on transfer agreements, referee reports obtained elsewhere may or may not be included in this compilation. Referee reports are anonymous unless the Referee chooses to sign their reports.)

1st Editorial Decision **14th Jul 2022**

Dear Dr. Conte,

Thank you for the submission of your research manuscript to EMBO reports. I have now received the reports from the three referees that were asked to evaluate your study, which can be found at the end of this email.

As you will see, the referees think that these findings are of interest. However, all referees have several comments, concerns, and suggestions, indicating that a major revision of the manuscript is necessary to allow publication of the study in EMBO reports. As the reports are below, and all their points need to be addressed, I will not detail them here.

Given the constructive referee comments, we would like to invite you to revise your manuscript with the understanding that all referee concerns must be addressed in the revised manuscript and in a detailed point-by-point response. Acceptance of your manuscript will depend on a positive outcome of a second round of review. It is EMBO reports policy to allow a single round of revision only and acceptance of the manuscript will therefore depend on the completeness of your responses included in the next, final version of the manuscript.

Revised manuscripts should be submitted within three months of a request for revision. Please contact me to discuss the revision (also by video chat) if you have questions or comments regarding the revision, or should you need additional time.

When submitting your revised manuscript, please also carefully review the instructions that follow below.

PLEASE NOTE THAT upon resubmission revised manuscripts are subjected to an initial quality control prior to exposition to re-review. Upon failure in the initial quality control, the manuscripts are sent back to the authors, which may lead to delays. Frequent reasons for such a failure are the lack of the data availability section (please see below) and the presence of statistics based on n=2 (the authors are then asked to present scatter plots or provide more data points).

When submitting your revised manuscript, we will require:

1) a .docx formatted version of the final manuscript text (including legends for main figures, EV figures and tables), but without the figures included. Figure legends should be compiled at the end of the manuscript text.

2) individual production quality figure files as .eps, .tif, .jpg (one file per figure), of main figures and EV figures. Please upload these as separate, individual files upon re-submission.

The Expanded View format, which will be displayed in the main HTML of the paper in a collapsible format, has replaced the Supplementary information. You can submit up to 5 images as Expanded View. Please follow the nomenclature Figure EV1, Figure EV2 etc. The figure legend for these should be included in the main manuscript document file in a section called Expanded View Figure Legends after the main Figure Legends section. Additional Supplementary material should be supplied as a single pdf file labeled Appendix. The Appendix should have page numbers and needs to include a table of content on the first page (with page numbers) and legends for all content. Please follow the nomenclature Appendix Figure Sx, Appendix Table Sx etc. throughout the text, and also label the figures and tables according to this nomenclature.

For more details, please refer to our guide to authors:
http://www.embopress.org/page/journal/14693178/authorguide#manuscriptpreparation

Please consult our guide for figure preparation:
http://wol-prod-cdn.literatumonline.com/pb-assets/embo-site/EMBOPress_Figure_Guidelines_061115-1561436025777.pdf

See also the guidelines for figure legend preparation:
https://www.embopress.org/page/journal/14693178/authorguide#figureformat

3) a complete author checklist, which you can download from our author guidelines (https://www.embopress.org/page/journal/14693178/authorguide). Please insert page numbers in the checklist to indicate where the requested information can be found in the manuscript. The completed author checklist will also be part of the RPF.

Please also follow our guidelines for the use of living organisms, and the respective reporting guidelines:
http://www.embopress.org/page/journal/14693178/authorguide#livingorganisms

4) that primary datasets produced in this study (e.g. RNA-seq, ChIP-seq, structural and array data) are deposited in an appropriate public database. If no primary datasets have been deposited, please also state this in a dedicated section (e.g. 'No primary datasets have been generated and deposited'), see below.

See also: http://embor.embopress.org/authorguide#datadeposition

Please remember to provide a reviewer password if the datasets are not yet public.

The accession numbers and database should be listed in a formal "Data Availability" section (placed after Materials & Methods) that follows the model below. This is now mandatory (like the COI statement). Please note that the Data Availability Section is restricted to new primary data that are part of this study. This section is mandatory. As indicated above, if no primary datasets have been deposited, please state this in this section

Data availability

The datasets produced in this study are available in the following databases:

- RNA-Seq data: Gene Expression Omnibus GSE46843 (https://www.ncbi.nlm.nih.gov/geo/query/acc.cgi?acc=GSE46843)
- [data type]: [name of the resource] [accession number/identifier/doi] ([URL or identifiers.org/DATABASE:ACCESSION])

*** Note - All links should resolve to a page where the data can be accessed. ***

Moreover, I have these editorial requests:

6) We strongly encourage the publication of original source data with the aim of making primary data more accessible and transparent to the reader. The source data will be published in a separate source data file online along with the accepted manuscript and will be linked to the relevant figure. If you would like to use this opportunity, please submit the source data (for example scans of entire gels or blots, data points of graphs in an excel sheet, additional images, etc.) of your key experiments together with the revised manuscript. If you want to provide source data, please include size markers for scans of entire gels, label the scans with figure and panel number, and send one PDF file per figure.

7) Our journal encourages inclusion of *data citations in the reference list* to directly cite datasets that were re-used and obtained from public databases. Data citations in the article text are distinct from normal bibliographical citations and should directly link to the database records from which the data can be accessed. In the main text, data citations are formatted as follows: "Data ref: Smith et al, 2001" or "Data ref: NCBI Sequence Read Archive PRJNA342805, 2017". In the Reference list, data citations must be labeled with "[DATASET]". A data reference must provide the database name, accession number/identifiers and a resolvable link to the landing page from which the data can be accessed at the end of the reference. Further instructions are available at: http://www.embopress.org/page/journal/14693178/authorguide#referencesformat

8) Regarding data quantification and statistics, please make sure that the number "n" for how many independent experiments were performed, their nature (biological versus technical replicates), the bars and error bars (e.g. SEM, SD) and the test used to calculate p-values is indicated in the respective figure legends (also for potential EV figures and all those in the final Appendix). Please also check that all the p-values are explained in the legend, and that these fit to those shown in the figure. Please provide statistical testing where applicable. Please avoid the phrase 'independent experiment', but clearly state if these were biological or technical replicates. See also:
http://www.embopress.org/page/journal/14693178/authorguide#statisticalanalysis

9) Please also note our reference format:
http://www.embopress.org/page/journal/14693178/authorguide#referencesformat

10) Please add scale bars of similar style and thickness to all the microscopic images (main and EV figures), using clearly visible black or white bars (depending on the background). Please place these in the lower right corner of the images themselves. Please do not write on or near the bars in the image but define the size in the respective figure legend.

11) We updated our journal's competing interests policy in January 2022 and request authors to consider both actual and perceived competing interests. Please review the policy https://www.embopress.org/competing-interests and update your competing interests if necessary. Please name this section 'Disclosure and Competing Interests Statement' and put it after the Acknowledgements section.

12) Please have your revised manuscript carefully proofread by a native speaker.

I look forward to seeing a revised version of your manuscript when it is ready. Please let me know if you have questions or comments regarding the revision.

Please use this link to submit your revision: https://embor.msubmit.net/cgi-bin/main.plex

Yours sincerely,

Achim Breiling

Senior Editor
EMBO Reports

Referee #1:

In this study, Donne et al. identified the E3 ubiquitin ligase praja2, as a novel ciliary protein, that mainly resides at the basal body and the transition zone region. The authors found that praja2 can directly bind to the BBSome components BBS1, BBS2, BBS4, and BBS7 and its amino acid residues 550-570 are essential for interacting with BBS1 and BBS2. The authors next found that praja2 ubiquitylates both BBS1 and BBS2 through the cAMP-PKA-praja2 axis and identified K143 as the ubiquitylation site of BBS1. The authors further found that ubiquitylation of BBS1 K143 not only can stabilize the BBSome but also facilitates the BBSome binding to BBS3/ARL6, mediating BBSome targeting to the ciliary membrane. Ubiquitylation-defective BBS1 mutant impedes GPCR trafficking and sonic hedgehog-dependent gene transcription, which causes medaka fish defective in ciliogenesis, embryonic development, and morphogenesis of photoreceptors. This study provides researchers new information for understanding ciliary maintenance of the BBSome: paraja2 ubiquitylation of the BBSome component BBS1 controls BBSome entry into cilia, thus providing a molecular mechanism underlying Bardet-biedl syndrome (BBS). However, I do have two concerns that need to be addressed.

Major concerns:

1) I have no doubt about the essentiality of the BBSome in promoting the movement of the activated GPCRs out of primary cilia. For this case, either disrupted BBSome ciliary cycling (the BBSome can enter cilia in this case) or lack of the BBSome in cilia can cause the same ciliary accumulation phenotype of the activated GPCRs as the BBSome acts as an adaptor between GPCRs and IFT. BBS3 functions both inside and outside cilia. Inside cilia, BBS3-mediated BBSome targeting to the ciliary membrane for receptor loading was "proposed" according to the biochemical analysis performed on whole cell but not ciliary extracts (Jin et al., 2010, Cell; Liu et al., 2021 eLife). Even for a single regulating factor like BBS3, its function observed in the cell body/basal body cannot be simply applied to reflect its function in cilia. For the BBS3 case, mammalian BBS3 binds the BBSome as its effector in the cell body and this is critical for the BBSome to appear inside cilia (Jin et al., 2010, Cell). This observation has been extended in Chlamydomonas model by showing that IFT22/RABL5 binds BBS3/ARL6 to form a heterodimeric IFT22/BBS3 in the cell body and this binding does not rely on their nucleotide state (Xue et al., 2020, PNAS). When they both are in a GTP-bound configuration, IFT22/BBS3 binds the BBSome as a BBS3-specific effector and recruits the BBSome to the basal body (Xue et al., 2020, PNAS). According to the authors, depletion of paraja2 by siRNA resulted in loss of cilia as expected and ciliary accumulation of the activated GPR161 and SSTR3 (Fig. a-f). These outcomes were caused by the inability of the BBSome to enter cilia as BBS1 K143R, as a BBSome marker, disappeared in cilia (Fig. 5). The evidence clearly showed that paraja2 ubiquitylation of BBS1 eventually promotes ciliary entry of the BBSome. By such a way, the activated GPCRs accumulate in cilia simply because of lack of the BBSome (the cargo adaptor for IFT) in cilia. Based on this, I strongly suggest the authors, if possible, to check if the BBSome is defective in targeting to the basal body without paraja2. If yes, the authors can announce that paraja2 controls GPCR removal out of cilia by mediating the BBSome targeting to the basal body. By the way, I also strongly suggest to the authors to cite the references as accurate and complete as possible in the introduction section to help the readers to have a full and up-to-date view on the BBSome ciliary cycling and signaling.

2) As mentioned above, the authors showed data to support that paraja2 controls BBSome intracellular trafficking for ciliary entry. It probably controls BBSome targeting to the basal body and lack of paraja2 will cause BBSome unavailable at the basal body for entering cilia (this is my guess). However, a microseconds-long molecular dynamics (MD) simulation only provides inadequate evidence for the authors to draw a conclusion that ubiquitylation of BBS1 stabilizes the BBSome. To avoid possible mistakes, the authors are suggested to provide biochemical evidence to strengthen this conclusion. For examples, the authors at least can quantify the BBSome in whole cell extracts by performing immunoblotting to see if the BBSome is degraded without paraja2.

Some minors:

I do find many inaccurate scientific description, grammatical errors, and typos all over the manuscript. Listed below are only four of them:
1) Lines 33-34: "Genes mutated in BBS encode for components of the BBSome, a conserved.....". Genes mutated in BBS encode for not only components of the BBSome entity but also many regulators that mediates BBSome ciliary cycling.
2) Line 38: "....and regulator of BBSome". It should be "... and the regulator of the BBSome".
3) Lines 53-54: "Mutations of 14 different BBS genes have been ....". As I know, a total of at least 22 BBS genes have been identified so far. Please cite the right reference.
4) Line 58-61: "......that acts as adaptor protein between the membrane and the intraflagellar transport (IFT) machinery". We don't call BBS3 as an adaptor protein.

And numerous others.......... I strongly suggest the authors to find someone to do some edits on this manuscript.

Referee #2:

In this manuscript, Delle Donne et. al. expand on their previous work in Senatore et al 2021, in which the PKA-mediated E3 ubiquitin ligase praja2 is important in regulating ciliogenesis. In this manuscript, the authors identify that praja2 localizes to the transition zone, and identified praja2 interaction with BBS1, as well as other BBsome components. They further identify K143 as a critical ubiquitin-acceptor residue in BBS1, and the mutation of the stated residue results in failure of BBS1 to localize to the cilium, as well as ciliary exiting defects from signaling proteins. Their modeling of molecular dynamics of the octameric BBsome suggests that monoubiquitylation of BBS1 reduces the flexibility of complex, perhaps affecting the role in trafficking, and this data is quite nicely not overstated in the manuscript. Finally, overexpression of the K143 allele in medaka fish results in a global Bardet-Biedl Syndrome like phenotype, recapitulating the clinical phenotype and confirming a role of BBS1 ubiquitylation in proper ciliary trafficking.

This work presents a novel layer in ubiquitin-regulation of ciliary processes, as well as a new functional role for ubiquitin in contrast to the canonical targeting for degradation. The immunoprecipitation work is quite convincing, and this paper deserves consideration in EMBO Reports once the following issues are addressed.

Major points:

1. While the immunofluorescence experiments in the BBS1 entry, GPR161/SSTR3 exit are convincing, the immunofluorescence experiments in the photocreceptor imaging are less clear. The difference between rhodopsin localization and ultrastructure in WT vs. mutant appears marginal at best. The model put forth, in the panel to the right of Fig6f is also confusing. The photoreceptor color matches the rhodopsin staining, making it falsely associated with rhodopsin staining, and there is no quantification of the mislocalization of Arl13b and BBS1 provided in the text. Furthermore, the differences in the connecting cilium do not match how it was described in the text. If anything, the Arl13b staining is sparser, showing less connecting cilia. Lastly, it is difficult to gauge what is being quantified in Fig6 g,h. Rhodopsin localization is confined to the outer segment; it is unclear how inner segment length can be measured without a marker. There is nothing in the materials and methods that can shed light on how these measurements were performed. Staining for outer segment discs or electron microscopy sections would be a more convincing way to show this dysmorphia.

2. The manuscript needs to cite references more thoroughly. For example: line 62-63 "Arl6 binding to BBsome induces conformational changes", line 93-94 "recently praja2/PKA complex has been identified as a component of a scaffold platform assembled a centrosome/basal body by TBC1D31" or line 207-208 "BBsome-mediated removal of GPCRs requires ubiquitylation of receptors by -arrestin-mediated-mechanism", need references.

3. As mentioned in point 1, some methods are lacking. How is ciliary localization measured in Fig4d,e and 5b,d?

Minor points:

1. The downregulation of cilia-signaling mRNAs in ARPE-19 is robust. However, no information is given regarding the control transcripts associated with the cilia panel. What is identify of the upregulated genes located in the bottom right quadrant of Fig5f? Are all these transcripts being downregulated because markers for cell death are being downregulated? Furthermore, this transcriptomic data shows significant downregulation of BBS2 and BBS4 in response to overexpression of BBS1K143R. This data contrasts with the transcriptomic data presented in the Masek et. al. 2022 (reference 50) which shows no significant changes in eye specific BBsome component expression upon BBsome depletion in 5 and 10 dpf zebrafish larvae. Can the authors comment on this fundamental difference?

2. Spelling errors in the manuscript and methods make the text difficult to read. For example, Fig 6b "phenotipe", Fig 6e "ciulium". Additionally, there are randomly highlighted words all over the text.

Referee #3:

Cilia are eukaryotic appendage-like organelles whose malfunction results in a variety of human diseases also known ciliopathy. Bardet-Biedl syndrome (BBS) is a ciliopathy disease and mutations in at least 20 different genes are reported to cause BBS. Eight proteins encoded by BBS genes form a complex called BBSome. In this submitted work, the authors characterised the role of E3 ubiquitin ligase praja2 in BBSome related functions. The same group (Senatore et al 2021) previously reported the localization of praja2 to the centrosome and utilized praja2 as a bait to identify praja2 interactors, which revealed that praja2 forms a complex with TBC1D3, and the TBC1D31/praja2 complex comprises OFD1.

They expanded on the previous study by demonstrating that praja2 localizes to cilia. Using yeast two-hybrid screening and co-immunoprecipitation (endogenous and exogenous), they identified BBS2 protein, a component of the BBSome complex, as an interactor of praja2. Co-immunoprecipitation analysis showed that praja2 also interacts with BBS1, a component of the BBSome complex, suggesting a potential functional interaction between BBSome and praja2. The authors also narrowed down

the region in praja2 proteins for interaction with BBS1 and BBS2. Subsequently, praja2 was found to ubiquitylate both BBS1 and BBS2. Both GPCR-cAMP activation and PKA mediated phosphorylation of praja2 are essentially needed for praja2 mediated ubiquitylation of BBS1 and BBS2. The identified the amino acid position in BBS1 for praja2 mediated ubiquitylation. When the ubiquitylated site inBBS1 (K143R) was eliminated, the binding of BBS3 (ARL6) to BBS1 was reduced.

The paper is very well-written, with neat and straightforward figures. After some revisions, I believe the work, given its significance, is appropriate for publishing in EMBO Reports.

1- Proximal cilia localization of praja2 is clear, but author stated the preferable distribution of praja2 at basal body and transition zone, and it would be great to resolve the exact localisation of praja2 within cilia by co-staining of praja2 with basal body and transition zone markers.

2- Given that BBS1K143R fails to enter cilia, is the localization of wild type BBS1 impacted in praja2 depleted cells?

3- This reviewer noticed the overall weakness and inconsistency of the statistical analysis. They may deem p* <0.1, ***p <0.01 or ***p <0.05 to be statistically significant. For example, is measurement with a total of 25 cells counting adequate to reach a conclusion in Figure 4B, D, and F? Because the provided p value is based on a **p <0.01 and a considerable increase in the number of measurements would improve the p value.

4- Administration of wild type BBS1 at various doses (Figure 8SA and B) resulted in changes in cilium length. The cilium length of wild type animals injected with 50 ng/ul BBS1 appears comparable to that of 5 ng/ul BBS1K143R Overexpression analysis is often difficult to interpret; might a rescue experiment utilizing Medaka larvae missing Bbs1 be conducted with wild type Bbs1 and Bbs1K143R?

Minor points:

- p.3 line59 " BBS8 through the BB18" should be "BBS8 through the BBS18" - p.7 line186 " of BB1 to" should be "of BBS1 to" - There are some places where text was bolded, and they should be corrected.

Referee #1:

In this study, Donne et al. identified the E3 ubiquitin ligase praja2, as a novel ciliary protein, that mainly resides at the basal body and the transition zone region. The authors found that praja2 can directly bind to the BBSome components BBS1, BBS2, BBS4, and BBS7 and its amino acid residues 550-570 are essential for interacting with BBS1 and BBS2. The authors next found that praja2 ubiquitylates both BBS1 and BBS2 through the cAMP-PKA-praja2 axis and identified K143 as the ubiquitylation site of BBS1. The authors further found that ubiquitylation of BBS1 K143 not only can stabilize the BBSome but also facilitates the BBSome binding to BBS3/ARL6, mediating BBSome targeting to the ciliary membrane. Ubiquitylation-defective BBS1 mutant impedes GPCR trafficking and sonic hedgehog-dependent gene transcription, which causes medaka fish defective in ciliogenesis, embryonic development, and morphogenesis of photoreceptors. This study provides researchers new information for understanding ciliary maintenance of the BBSome: paraja2 ubiquitylation of the BBSome component BBS1 controls BBSome entry into cilia, thus providing a molecular mechanism underlying Bardet-Biedl syndrome (BBS).

R. We wish to thank the Reviewer for his/her positive comments on our manuscript and to consider that our study provides new information for understanding the mechanism regulating BBSome maintenance within the cilium.

However, I do have two concerns that need to be addressed.

Major concerns:

1) I have no doubt about the essentiality of the BBSome in promoting the movement of the activated GPCRs out of primary cilia. For this case, either disrupted BBSome ciliary cycling (the BBSome can enter cilia in this case) or lack of the BBSome in cilia can cause the same ciliary accumulation phenotype of the activated GPCRs as the BBSome acts as an adaptor between GPCRs and IFT. BBS3 functions both inside and outside cilia. Inside cilia, BBS3-mediated BBSome targeting to the ciliary membrane for receptor loading was "proposed" according to the biochemical analysis performed on whole cell but not ciliary extracts (Jin et al., 2010, Cell; Liu et al., 2021 eLife). Even for a single regulating factor like BBS3, its function observed in the cell body/basal body cannot be simply applied to reflect its function in cilia. For the BBS3 case, mammalian BBS3 binds the BBSome as its effector in the cell body and this is critical for the BBSome to appear inside cilia (Jin et al., 2010, Cell). This observation has been extended in Chlamydomonas model by showing that IFT22/RABL5 binds BBS3/ARL6 to form a heterodimeric IFT22/BBS3 in the cell body and this binding does not rely on their nucleotide state (Xue et al., 2020, PNAS). When they both are in a GTP-bound configuration, IFT22/BBS3 binds the BBSome as a BBS3-specific effector and recruits the BBSome to the basal body (Xue et al., 2020, PNAS). According to the authors, depletion of paraja2 by siRNA resulted in loss of cilia as expected and ciliary accumulation of the activated GPR161 and SSTR3 (Fig. a-f). These outcomes were caused by the inability of the BBSome to enter cilia as BBS1 K143R, as a BBSome marker, disappeared in cilia (Fig. 5). The evidence clearly showed that paraja2 ubiquitylation of BBS1 eventually promotes ciliary entry of the BBSome. By such a way, the activated GPCRs accumulate in cilia simply because of lack of the BBSome (the cargo adaptor for IFT) in cilia. Based on this, I strongly suggest the authors, if possible, to check if the BBSome is defective in targeting to the basal body without paraja2. If yes, the authors can announce that paraja2 controls GPCR removal out of cilia by mediating the BBSome targeting to the basal body. By the way, I also strongly suggest to the authors to cite the references as accurate and complete as possible in the introduction section to help the readers to have a full and up-to-date view on the BBSome ciliary cycling and signaling.

R. The point raised by the Reviewer is very important. To address it, we performed immunostaining experiments for BBSome subunits in RPE cells devoided of praja2. The results shown in the ***Figure EV4b-e*** *indicates that praja2 is, indeed, required for BBSome targeting to the basal body. In particular, we show that accumulation of BBS1 and BBS2 at the base of the primary cilium was significantly reduced in cells lacking praja2, compared to control cells. Moreover, to have a full and up-to-date view on the BBSome ciliary*

*trafficking and signaling, as pointed by the Reviewer, we have now included and discussed the appropriate references in the Introduction Section (**Refs. 5, 6**)*

2) As mentioned above, the authors showed data to support that paraja2 controls BBSome intracellular trafficking for ciliary entry. It probably controls BBSome targeting to the basal body and lack of paraja2 will cause BBSome unavailable at the basal body for entering cilia (this is my guess). However, a microseconds-long molecular dynamics (MD) simulation only provides inadequate evidence for the authors to draw a conclusion that ubiquitylation of BBS1 stabilizes the BBSome. To avoid possible mistakes, the authors are suggested to provide biochemical evidence to strengthen this conclusion. For examples, the authors at least can quantify the BBSome in whole cell extracts by performing immunoblotting to see if the BBSome is degraded without paraja2.

*R. As suggested, we have monitored expression levels of BBSome subunits in RPE cells following depletion of praja2, compared to control cells. **Figure EV4f** shows that downregulation of praja2 had no major effects on the levels of BBSome subunits.*

Some minors:

I do find many inaccurate scientific description, grammatical errors, and typos all over the manuscript. Listed below are only four of them:

1) Lines 33-34: "Genes mutated in BBS encode for components of the BBSome, a conserved.....". Genes mutated in BBS encode for not only components of the BBSome entity but also many regulators that mediates BBSome ciliary cycling.
2) Line 38: "....and regulator of BBSome". It should be "... and the regulator of the BBSome".
3) Lines 53-54: "Mutations of 14 different BBS genes have been ....". As I know, a total of at least 22 BBS genes have been identified so far. Please cite the right reference.
4) Line 58-61: "......that acts as adaptor protein between the membrane and the intraflagellar transport (IFT) machinery". We don't call BBS3 as an adaptor protein.
And numerous others.......... I strongly suggest the authors to find someone to do some edits on this manuscript.

R. Many thanks to the Reviewer for the suggestions. We have now made the appropriate corrections to the whole text.

Referee #2:

In this manuscript, Delle Donne et. al. expand on their previous work in Senatore et al 2021, in which the PKA-mediated E3 ubiquitin ligase praja2 is important in regulating ciliogenesis. In this manuscript, the authors identify that praja2 localizes to the transition zone, and identified praja2 interaction with BBS1, as well as other BBSome components. They further identify K143 as a critical ubiquitin-acceptor residue in BBS1, and the mutation of the stated residue results in failure of BBS1 to localize to the cilium, as well as ciliary exiting defects from signaling proteins. Their modeling of molecular dynamics of the octameric BBSome suggests that monoubiquitylation of BBS1 reduces the flexibility of complex, perhaps affecting the role in trafficking, and this data is quite nicely not overstated in the manuscript. Finally, overexpression of the K143 allele in medaka fish results in a global Bardet-Biedl Syndrome like phenotype, recapitulating the clinical phenotype and confirming a role of BBS1 ubiquitylation in proper ciliary trafficking.
This work presents a novel layer in ubiquitin-regulation of ciliary processes, as well as a new functional role for ubiquitin in contrast to the canonical targeting for degradation. The immunoprecipitation work is quite convincing, and this paper deserves consideration in EMBO Reports once the following issues are addressed.

R. Many thanks to the Reviewer and we are very happy for her/his endorsement to publish our manuscript in EMBO reports.

Major points:

1. While the immunofluorescence experiments in the BBS1 entry, GPR161/SSTR3 exit are convincing, the immunofluorescence experiments in the photoreceptor imaging are less clear. The difference between rhodopsin localization and ultrastructure in WT vs. mutant appears marginal at best. The model put forth, in the panel to the right of Fig6f is also confusing. The photoreceptor color matches the rhodopsin staining, making it falsely associated with rhodopsin staining, and there is no quantification of the mislocalization of Arl13b and BBS1 provided in the text.

R. We apologize because we have created some confusion by matching photoreceptor color with the rhodopsin staining. We modified the color of photoreceptor in the new Fig.7, accordingly. Moreover, we agree with the Reviewer that it is an important issue to better explain; for this reason, to clarify the mislocalizion of BBS1 associated to an incorrect rod's structure, we now provide the results of the Airyscan super-resolution images in the new Figure 7 (panels a). Moreover, we add new data concerning the quantification of the mislocalization of Arl13b and BBS1 (Figure 7 f-g).

Furthermore, the differences in the connecting cilium do not match how it was described in the text. If anything, the Arl13b staining is sparser, showing less connecting cilia. Lastly, it is difficult to gauge what is being quantified in Fig6 g,h. Rhodopsin localization is confined to the outer segment; it is unclear how inner segment length can be measured without a marker. There is nothing in the materials and methods that can shed light on how these measurements were performed. Staining for outer segment discs or electron microscopy sections would be a more convincing way to show this dysmorphia.

R. We apologize for the lack of the clarity. We agree with the Reviewer that it is an important concern that helped us to improve the quality of the data presented. Indeed, we now provide this information in the revised manuscript, the text now reads (Page 10 Line 306-308) "Notably, ectopic expression of BBS1K143R, but not BBS1WT, was sufficient to induce a reduction of rod inner segment (IS) length compared to native rods, suggesting a strong reduction of cilia length (Fig. 7d-e).". Furthermore, we largely expanded the Materials and Methods section to better explain how these measurements were performed.

2. The manuscript needs to cite references more thoroughly. For example: line 62-63 "Arl6 binding to BBsome induces conformational changes", line 93-94 "recently praja2/PKA complex has been identified as a component of a scaffold platform assembled a centrosome/basal body by TBC1D31" or line 207-208

"BBsome-mediated removal of GPCRs requires ubiquitylation of receptors by β-arrestin-mediated-mechanism", need references.

R. We apologize for the missing information. We have now added the appropriate references.

3. As mentioned in point 1, some methods are lacking. How is ciliary localization measured in Fig4d,e and 5b,d?

R. Apologies for the missing information. We have now added this information in the "Materials and Method" section (please, see line-659-661).

Minor points:

1. The downregulation of cilia-signaling mRNAs in ARPE-19 is robust. However, no information is given regarding the control transcripts associated with the cilia panel. What is identify of the upregulated genes located in the bottom right quadrant of Fig5f? Are all these transcripts being downregulated because markers for cell death are being downregulated?

*R. The **Figure 5f, 5g** was represented as fold decrease of ciliary genes mRNAs in BBS1 K143R-transfected cells, compared to BBS1 WT-expressing cells. To better represent the fold downregulation in the bar chart, all the genes in BBS1-expressing cells were set as 1. A new Table **(array K143R raw data)** is now included with the mean value of fold changes from 3 independent experiment in control cells (BBS1 WT) and test group (BBS1 K143R). In the lower right panel of **Figure 5f** are represented genes that in the new Table are indicated as positive values in the column Fold up-regulated or down-regulated genes. They were not included in the Bar charts because the p-values were not significant. Moreover, FACS analysis in the **new Appendix Fig. 6** shows that overexpression of BBS1 K143R mutant has no major effects on cell viability, compared to cells overexpressing BBS1 WT.*

1.Furthermore, this transcriptomic data shows significant downregulation of BBS2 and BBS4 in response to overexpression of BBS1K143R. This data contrasts with the transcriptomic data presented in the Masek et. al. 2022 (reference 50) which shows no significant changes in eye specific BBSome component expression upon BBSome depletion in 5 and 10 dpf zebrafish larvae. Can the authors comment on this fundamental difference?

R. The point raised by the Reviewer is important since there is a clear discrepancy between both studies. We believe that the main difference relies in the different experimental strategies used. In our study, we monitored changes of RNA transcripts in transiently transfected cells using a dominant negative mutant of BBS1 (BBS1 K143R). Under these conditions, the effects of a dominant variant of BBS1 are expected to be more severe on the ciliary transcriptional networks than in stable BBS1 KO line as reported in Masek et. al. 2022 (now ref. 57). Acute inactivation by a dominant negative mutant vs constitutive loss of a gene can make a difference. Regulatory adaptive mechanisms underlying the transcriptional program in stable knockout lines might explain the difference observed with the transient experimental model used in our work.

2. Spelling errors in the manuscript and methods make the text difficult to read. For example, Fig 6b "phenotipe", Fig 6e "ciulium". Additionally, there are randomly highlighted words all over the text.

R. Many thanks to the Reviewer for the suggestions. We have now made the appropriate corrections to the whole text.

Referee #3:

Cilia are eukaryotic appendage-like organelles whose malfunction results in a variety of human diseases also known ciliopathy. Bardet-Biedl syndrome (BBS) is a ciliopathy disease and mutations in at least 20 different genes are reported to cause BBS. Eight proteins encoded by BBS genes form a complex called BBSome. In this submitted work, the authors characterised the role of E3 ubiquitin ligase praja2 in BBSome related functions. The same group (Senatore et al 2021) previously reported the localization of praja2 to the centrosome and utilized praja2 as a bait to identify praja2 interactors, which revealed that praja2 forms a complex with TBC1D3, and the TBC1D31/praja2 complex comprises OFD1.

They expanded on the previous study by demonstrating that praja2 localizes to cilia. Using yeast two-hybrid screening and co-immunoprecipitation (endogenous and exogenous), they identified BBS2 protein, a component of the BBSome complex, as an interactor of praja2. Co-immunoprecipitation analysis showed that praja2 also interacts with BBS1, a component of the BBSome complex, suggesting a potential functional interaction between BBSome and praja2. The authors also narrowed down the region in praja2 proteins for interaction with BBS1 and BBS2. Subsequently, praja2 was found to ubiquitylate both BBS1 and BBS2. Both GPCR-cAMP activation and PKA mediated phosphorylation of praja2 are essentially needed for praja2 mediated ubiquitylation of BBS1 and BBS2. The identified the amino acid position in BBS1 for praja2 mediated ubiquitylation. When the ubiquitylated site inBBS1 (K143R) was eliminated, the binding of BBS3 (ARL6) to BBS1 was reduced.

The paper is very well-written, with neat and straightforward figures. After some revisions, I believe the work, given its significance, is appropriate for publishing in EMBO Reports.

R. Many thanks to the Reviewer for her/his endorsement to publish our manuscript in EMBO reports.

1- Proximal cilia localization of praja2 is clear, but author stated the preferable distribution of praja2 at basal body and transition zone, and it would be great to resolve the exact localisation of praja2 within cilia by co-staining of praja2 with basal body and transition zone markers.

*R. According to the Reviewer's suggestion, we repeated the double immunostaining analysis for praja2 and ciliary markers and confirmed that praja2 staining is mostly localized at the base of the primary cilium. However, few cells show some praja2 staining at the proximal segment of the cilium. However, we are not sure if this reflects a dynamic targeting of praja2 between basal body and the transition zone or an experimental artifact. To avoid any problem in interpreting the data, we decided to leave out this aspect from the manuscript (that would need further substantial work to support it). Accordingly, we rephrased the sentence in the manuscript stating that praja2 is mainly localized at the basal body (**please, see line 117-118**).*

2- Given that BBS1K143R fails to enter cilia, is the localization of wild type BBS1 impacted in praja2 depleted cells?

*R. This is an interesting point. To address it, we performed immunostaining experiments in RPE cells transiently transfected with siRNA targeting praja2 or control siRNAc and monitored the ciliary localization of endogenous BBS1 and BBS2. The findings reported in the **Figure EV4 b-e** show that downregulation of praja2 dramatically affected ciliary localization of both BBS subunits. Accordingly, we have included in the text a sentence that "the data support a role of praja2 in ciliary localization of the BBSome complex" (**please, see line 240-241**)*

3- This reviewer noticed the overall weakness and inconsistency of the statistical analysis. They may deem p* <0.1, ***p <0.01 or ***p <0.05 to be statistically significant. For example, is measurement with a total of 25 cells counting adequate to reach a conclusion in Figure 4B, D, and F? Because the provided p value is based

on a **p <0.01 and a considerable increase in the number of measurements would improve the p value.

R. We apologize for the lack of clarity. As indicated in the legend to the figure 4 (panels b, d, f), the p values were derived from a cumulative analysis of at least 4 independent experiments and for each experiment we scored 25 cells.

4- Administration of wild type BBS1 at various doses (Figure 8SA and B) resulted in changes in cilium length. The cilium length of wild type animals injected with 50 ng/ul BBS1 appears comparable to that of 5 ng/ul BBS1K143R Overexpression analysis is often difficult to interpret; might a rescue experiment utilizing Medaka larvae missing Bbs1 be conducted with wild type Bbs1 and Bbs1K143R?

R. We agree with the Reviewer that it is an important question that helped us to improve the quality of the data presented. As suggested by the Reviewer, we have carried out rescue experiments utilizing BBS1 morphant larvae depleted of BBS1 in which we have co-injected $BBS1^{WT}$ and $BBS1^{K143R}$*, respectively. The data are now shown in the modified* ***Figure 6*** *and described in the Results section.*

Minor points:

- p.3 line59 " BBS8 through the BB18" should be "BBS8 through the BBS18" - p.7 line186 " of BB1 to" should be "of BBS1 to" - There are some places where text was bolded, and they should be corrected.

R. We have made the suggested corrections to the text.

1st Revision - Editorial Decision 16th Dec 2022

Dear Prof. Conte

Thank you for the submission of your revised manuscript to EMBO reports. I apologize for the delay in handling your manuscript but we have now received the reports from former referee #1 and #3 (copied below). Referee #2 was unfortunately not available.

As you will see, referee 3 considers the revised version significantly improved but referee 1 raises some remaining concerns that I ask you to address in the manuscript and in a point-by-point response.

From the editorial side, there are also a number of things that need our attention:

- Please update the 'Conflict of interest' paragraph to our new 'Disclosure and competing interests statement'. For more information see
https://www.embopress.org/page/journal/14693178/authorguide#conflictsofinterest

- Please update the references to the alphabetical Harvard style. The abbreviation 'et al' should be used if more than 10 authors. You can download the respective EndNote file from our Guide to Authors
https://endnote.com/style_download/embo-reports/

- Please complete the author checklist by selecting the adequate responses from the pulldown menu in column 2.

- Please compare the funding information given in our online submission system and in the manuscript text and ensure that these two match.

- We recommend arranging the figure panels so that they can be called out in an alphabetical order. In this context we note that Figs 7F&G are called out before 7C. If possible, please rearrange. Moreover, callouts to Figure EV1, EV2, and EV3 are missing as well as callouts to the panels of Appendix Figs S3, S4 and S5. Please add this to the text where appropriate. Finally, there is a callout to "Supplementary Fig. S9A-H" which needs correcting/removing.

- Appendix: Please provide page numbers in the table of content and please use capital letters (A, B etc) for the figure panels.

- Movies: Please remove their legends from the Appendix and instead provide the legend as simple README.txt file. This text file is then zipped together with its movie and the zip file is uploaded. The nomenclature is Movie EV#.

- The Figure legends should follow after the Reference list.

- I attach to this email a related manuscript file with comments by our data editors. Please address all comments and upload a revised file with tracked changes with your final manuscript submission. I have also added some comments in the Abstract.

- Finally, EMBO reports papers are accompanied online by A) a short (1-2 sentences) summary of the findings and their significance, B) 2-3 bullet points highlighting key results and C) a synopsis image that is 550x300-600 pixels large (width x height) in PNG for JPG format. You can either show a model or key data in the synopsis image. Please note that the size is rather small and that text needs to be readable at the final size. Please send us this information along with the revised manuscript.

We look forward to seeing a final version of your manuscript as soon as possible.

With kind regards,

Martina Rembold, PhD
Senior Editor
EMBO reports

****

Referee #1:

The authors identified praja2 to preferentially localize to the basal body and the proximal ciliary section by immunostaining. They then found that praje2 binds to several single BBSome subunit including BBS1, BBS2, BBS4, and BBS7 by immunoprecipitation in vitro. They further defined the 550-570 residues of praja2 to be essential for BBS1 and BBS2 binding. The authors next by focusing on BBS1, confirmed K143 to be the BBS1 ubiquitin acceptor site and BBS1 (BBS2 as well) was ubiquitylated by praja2 in a forskolin-induced cAMP-dependent manner. They further found that BBS1 ubiquitylation is indispensable for BBS3 to bind to

BBS1 and is required for stabilizing the BBSome. The authors next found that BBS1 ubiquitylation controls BBSome translocation to the basal body. The lack of BBS1 ubiquitination thus impeded the BBSome to enter cilia because of its failure to target to the basal body, causing the ciliary accumulation of GPCRs like SSTR3 and GPR161 that exit cilia in a BBSome-dependent manner. Finally, they by overexpressing BBS1K14R in Madaka fish successfully observed BBS-like symptoms.

The BBSome biology is complicated as it has been implicated to function both outside and inside cilia for cargo pickup but via different pathways. In cytoplasm, BBS3 by interacting with IFT22 for forming an IFT22/BBS3 heterodimer (at least in Chlamydomonas reinhardtii) binds the BBSome via a direct interaction of BBS3 to the BBS1 component of the BBSome and this binding does not rely on the nucleotide state of BBS3 and IFT22. IFT22/BBS3 then recruits the BBSome to traffic from the cytoplasm to the basal body only when they both are GTP-bound (DOI: 10.1073/pnas.1901665117). Upon inside cilia, BBS3 promotes cargo-BBSome coupling at the ciliary tip and this process also needs the involvement of ARL13 (at least in C. reinhardtii), while how they cross talk for achieving this coupling remains uncertain (DOI: 10.1083/jcb.202201050; DOI: 10.7554/eLife.59119; DOI: 10.1016/j.devcel.2014.09.004). In my eyes, these data already given out a clear logic that praja2-mediated ubiquitylation of BBS1 promotes BBSome trafficking from the cell body to the basal body. The observation that GPCRs fail to exit cilia in the lack of the ubiquitylated BBS1 is caused as, in this situation, the BBSome is unavailable to enter cilia to serve as an IFT cargo adaptor. I believe these are the main findings of this study.

Based on the logic above, I sincerely require, again, the authors to carefully rephrase their whole manuscript in an accurate and concise way so that the readers can read much easier. I do appreciate the authors for providing novel knowledge to the ciliary field especially for the field of BBSome biology, while I don't think this manuscript at its current version is suitable for publication in EMBO Reports. As you can see, many confusions remain in the text though mostly due to unproper language usage.

Some other things to consider:

1. Lines 121-136, the authors claimed to investigate the mechanism how praja2 localizes to the cilium by determining its interaction with certain subunits of the BBSome both in vitro and in vivo, Praja2 binding to BBS1 and BBS2 indeed can prove its direct interaction with the BBSome, while this methodology does not provide evidence whether and how paraja2 regulates BBSome ciliary targeting. So, modify your description for avoiding possible overexpression.

2. As mentioned by the authors in the response to reviewers, BBSome abundance remained to be uninfluenced in the presence of BBS1 K143R, largely meaning that the BBSome is stable without BBS1 ubiquitylation. This is a conflict to your simulation suggesting that the BBSome become unstable without BBS1 ubiquitylation. The authors need to address this question for the section entitled Ubiquitylation of BBS1 is required for Arl6 binding and BBSome complex stability. If the data are not consistent, I prefer to believe the biochemical data rather than the simulation. Otherwise, it could be an overinterpretation or even a mistake.

3. Fig. EV4 provides critical data for showing praja2 recruitment of the BBSome to the basal body, the authors could consider moving them to the main Figure rather than in the supplementary.

4. Fig. 7f needs to modify for reflecting the major finding. At least the basal body recruitment of the BBSome in a praja2-dependent manner need to be added. Besides, BBS3 recruits the BBSome to the ciliary membrane for cargo (GPCR)-coupling. BBS3 in a GTP-bound state anchors to the ciliary membrane. BBS3 does not undergo IFT in cilia, so, it could not couple with the BBSome but transiently interact with the BBSome at the ciliary tip. More, according to the authors' data, cAMP activates BBS1 ubiquitylation through praja2 is supposed to happen in the cell body rather than at the basal body and inside cilia as suggested now.

5. The molecular mechanism of how praja2 controls BBSome basal body targeting through ubiquitylating BBS1 should be extensively discussed in the discussion section as this is the most important finding of this study.

Numerous grammatical errors need to be corrected. Listed below are only some of them:

Line 56: ".....of BBsome an octameric protein complex..." should be ".....of the BBSome, an octameric protein complex...."

Line 81: "ITF20-mediated" should be IFT20-mediated"

Line 120: ".....distributed at basal body." should be "....distributed at the basal body."

Line: 129: " ....at primary cilium" should be ".....at the primary cilium"

Line 192: BB18 should be BBS8.

Line 197: BBS18 should be BBS8.

Line 207: BBS18 should be BBS8.

Line 213: "requires intact the BBSome complex" should be "requires the intact BBSome complex".

Line 238 "...at basal body" should be " ....at the basal body"

And much more............

Referee #3:

They have done their best, and I am pleased with their efforts and responses. The manuscript is worthy of publication in EMBO Reports.

Referee #1:

The authors identified praja2 to preferentially localize to the basal body and the proximal ciliary section by immunostaining. They then found that praje2 binds to several single BBSome subunit including BBS1, BBS2, BBS4, and BBS7 by immunoprecipitation in vitro. They further defined the 550-570 residues of praja2 to be essential for BBS1 and BBS2 binding. The authors next by focusing on BBS1, confirmed K143 to be the BBS1 ubiquitin acceptor site and BBS1 (BBS2 as well) was ubiquitylated by praja2 in a forskolin-induced cAMP-dependent manner. They further found that BBS1 ubiquitylation is indispensable for BBS3 to bind to BBS1 and is required for stabilizing the BBSome. The authors next found that BBS1 ubiquitylation controls BBSome translocation to the basal body. The lack of BBS1 ubiquitination thus impeded the BBSome to enter cilia because of its failure to target to the basal body, causing the ciliary accumulation of GPCRs like SSTR3 and GPR161 that exit cilia in a BBSome-dependent manner. Finally, they by overexpressing BBS1K14R in Madaka fish successfully observed BBS-like symptoms.

The BBSome biology is complicated as it has been implicated to function both outside and inside cilia for cargo pickup but via different pathways. In cytoplasm, BBS3 by interacting with IFT22 for forming an IFT22/BBS3 heterodimer (at least in Chlamydomonas reinhardtii) binds the BBSome via a direct interaction of BBS3 to the BBS1 component of the BBSome and this binding does not rely on the nucleotide state of BBS3 and IFT22. IFT22/BBS3 then recruits the BBSome to traffic from the cytoplasm to the basal body only when they both are GTP-bound (DOI: 10.1073/pnas.1901665117). Upon inside cilia, BBS3 promotes cargo-BBSome coupling at the ciliary tip and this process also needs the involvement of ARL13 (at least in C. reinhardtii), while how they cross talk for achieving this coupling remains uncertain (DOI: 10.1083/jcb.202201050; DOI: 10.7554/eLife.59119; DOI: 10.1016/j.devcel.2014.09.004). In my eyes, these data already given out a clear logic that praja2-mediated ubiquitylation of BBS1 promotes BBSome trafficking from the cell body to the basal body. The observation that GPCRs fail to exit cilia in the lack of the ubiquitylated BBS1 is caused as, in this situation, the BBSome is unavailable to enter cilia to serve as an IFT cargo adaptor. I believe these are the main findings of this study.

Based on the logic above, I sincerely require, again, the authors to carefully rephrase their whole manuscript in an accurate and concise way so that the readers can read much easier. I do appreciate the authors for providing novel knowledge to the ciliary field especially for the field of BBSome biology, while I don't think this manuscript at its current version is suitable for publication in EMBO Reports. As you can see, many confusions remain in the text though mostly due to unproper language usage.

R. *We wish to thank the Reviewer for his/her positive comments on our manuscript and to consider that our study provides new information for understanding the mechanism regulating BBSome maintenance within the cilium. We also thanks for his/her suggestions. We have made all the suggested changes and revised the manuscript and figures accordingly.*

Some other things to consider:

1. Lines 121-136, the authors claimed to investigate the mechanism how praja2 localizes to the cilium by determining its interaction with certain subunits of the BBSome both in vitro and in vivo, Praja2 binding to BBS1 and BBS2 indeed can prove its direct interaction with the BBSome, while this methodology does not provide evidence whether and how praja2 regulates BBSome ciliary targeting. So, modify your description for avoiding possible overexpression.

R. *We thank the Reviewer for his/her comment. Accordingly, we modified the text for avoiding possible overexpression.*

2. As mentioned by the authors in the response to reviewers, BBSome abundance remained to be uninfluenced in the presence of BBS1 K143R, largely meaning that the BBSome is stable without BBS1 ubiquitylation. This is a conflict to your simulation suggesting that the BBSome become unstable without BBS1 ubiquitylation. The authors need to address this question for the section entitled Ubiquitylation of BBS1 is required for Arl6 binding and BBSome complex stability. If the data are not consistent, I prefer to believe the biochemical data rather than the simulation. Otherwise, it could be an overinterpretation or even a mistake.

R. *We apologize for the lack of clarity and now we have fixed this inaccuracy by better elucidating this aspect in the revised manuscript.*

3. Fig. EV4 provides critical data for showing praja2 recruitment of the BBSome to the basal body, the authors could consider moving them to the main Figure rather than in the supplementary.

R. *We thank the Reviewer for his/her comment that improves our manuscript. Accordingly, we moved the Fig. EV4 to the new main Figure 6.*

4. Fig. 7f needs to modify for reflecting the major finding. At least the basal body recruitment of the BBSome in a praja2-dependent manner need to be added. Besides, BBS3 recruits the BBSome to the ciliary membrane for cargo (GPCR)-coupling. BBS3 in a GTP-bound state anchors to the ciliary membrane. BBS3 does not undergo IFT in cilia, so, it could not couple with the BBSome but transiently interact with the BBSome at the ciliary tip. More, according to the authors' data, cAMP activates BBS1 ubiquitylation through praja2 is supposed to happen in the cell body rather than at the basal body and inside cilia as suggested now.

R. *We thank the Reviewer for his/her comment. Accordingly, we modified the Figure 7 f (now Figure 8) for avoiding possible confusions.*

5. The molecular mechanism of how praja2 controls BBSome basal body targeting through ubiquitylating BBS1 should be extensively discussed in the discussion section as this is the most important finding of this study.

R. *We have now added this information in the text. Accordingly, we modified the discussion to extensively discuss. how PJA2 controls BBSome basal body targeting through ubiquitylating BBS1.*

Numerous grammatical errors need to be corrected. Listed below are only some of them:
Line 56: ".....of BBsome an octameric protein complex..." should be ".....of the BBSome, an octameric protein complex...."
Line 81: "ITF20-mediated" should be IFT20-mediated"
Line 120: ".....distributed at basal body." should be "....distributed at the basal body."
Line: 129: " ....at primary cilium" should be ".....at the primary cilium"
Line 192: BB18 should be BBS8.
Line 197: BBS18 should be BBS8.
Line 207: BBS18 should be BBS8.
Line 213: "requires intact the BBSome complex" should be "requires the intact BBSome complex".
Line 238 "...at basal body" should be " ....at the basal body"
And much more............

***R.** We also apologize for the presence in the previous version of a number of inaccuracies. We corrected all the typos throughout the manuscript and revised grammatical mistakes. We hope the new version of our manuscript will satisfy the Reviewer's worries.*

Referee #3:

They have done their best, and I am pleased with their efforts and responses. The manuscript is worthy of publication in EMBO Reports.

***R.** We thank the Reviewer for the recognition of our study and greatly appreciate the efforts the Reviewer for the improvement of our manuscript.*

Prof. Ivan Conte
Telethon Institute of Genetics and Medicine, Pozzuoli
TIGEM
Via Campi Flegrei 34
Pozzuoli, Naples 80078
Italy

Dear Prof. Conte,

I am very pleased to accept your manuscript for publication in the next available issue of EMBO reports. Thank you for your contribution to our journal.

At the end of this email I include important information about how to proceed. Please ensure that you take the time to read the information and complete and return the necessary forms to allow us to publish your manuscript as quickly as possible.

As part of the EMBO publication's Transparent Editorial Process, EMBO reports publishes online a Review Process File to accompany accepted manuscripts. As you are aware, this File will be published in conjunction with your paper and will include the referee reports, your point-by-point response and all pertinent correspondence relating to the manuscript.

If you do NOT want this File to be published, please inform the editorial office within 2 days, if you have not done so already, otherwise the File will be published by default [contact: emboreports@embo.org]. If you do opt out, the Review Process File link will point to the following statement: "No Review Process File is available with this article, as the authors have chosen not to make the review process public in this case." Please note that the author checklist will still be published even if you opt out of the transparent process.

Thank you again for your contribution to EMBO reports and congratulations on a successful publication. Please consider us again in the future for your most exciting work.

Yours sincerely,

Martina Rembold, PhD
Senior Editor
EMBO reports

****

THINGS TO DO NOW:

Please note that you will be contacted by Wiley Author Services to complete licensing and payment information. The required 'Page Charges Authorization Form' is available here: https://www.embopress.org/pb-assets/embo-site/er_apc.pdf - please download and complete the form and return to embopressproduction@wiley.com

EMBO Press participates in many Publish and Read agreements that allow authors to publish Open Access with reduced/no publication charges. Check your eligibility: https://authorservices.wiley.com/author-resources/Journal-Authors/open-access/affiliation-policies-payments/index.html

You will receive proofs by e-mail approximately 2-3 weeks after all relevant files have been sent to our Production Office; you should return your corrections within 2 days of receiving the proofs.

Please inform us if there is likely to be any difficulty in reaching you at the above address at that time. Failure to meet our deadlines may result in a delay of publication, or publication without your corrections.

All further communications concerning your paper should quote reference number EMBOR-2022-55571V3 and be addressed to emboreports@wiley.com.

Should you be planning a Press Release on your article, please get in contact with emboreports@wiley.com as early as possible, in order to coordinate publication and release dates.

EMBO Press Author Checklist

Corresponding Author Name: Antonio Feliciello	
Journal Submitted to: Embo Reports	
Manuscript Number: EMBOR-2022-55571V2	

USEFUL LINKS FOR COMPLETING THIS FORM
The EMBO Journal - Author Guidelines
EMBO Reports - Author Guidelines
Molecular Systems Biology - Author Guidelines
EMBO Molecular Medicine - Author Guidelines

Reporting Checklist for Life Science Articles (updated January 2022)
This checklist is adapted from Materials Design Analysis Reporting (MDAR) Checklist for Authors. MDAR establishes a minimum set of requirements in transparent reporting in the life sciences (see Statement of Task: 10.31222/osf.io/9sm4x). Please follow the journal's guidelines in preparing your manuscript.
Please note that a copy of this checklist will be published alongside your article.

Abridged guidelines for figures
1. Data
The data shown in figures should satisfy the following conditions:
- the data were obtained and processed according to the field's best practice and are presented to reflect the results of the experiments in an accurate and unbiased manner.
- ideally, figure panels should include only measurements that are directly comparable to each other and obtained with the same assay.
- plots include clearly labeled error bars for independent experiments and sample sizes. Unless justified, error bars should not be shown for technical replicates.
- if n<5, the individual data points from each experiment should be plotted. Any statistical test employed should be justified.
- Source Data should be included to report the data underlying figures according to the guidelines set out in the authorship guidelines on Data Presentation.

2. Captions
Each figure caption should contain the following information, for each panel where they are relevant:
- a specification of the experimental system investigated (eg cell line, species name).
- the assay(s) and method(s) used to carry out the reported observations and measurements.
- an explicit mention of the biological and chemical entity(ies) that are being measured.
- an explicit mention of the biological and chemical entity(ies) that are altered/varied/perturbed in a controlled manner.
- the exact sample size (n) for each experimental group/condition, given as a number, not a range;
- a description of the sample collection allowing the reader to understand whether the samples represent technical or biological replicates (including how many animals, litters, cultures, etc.).
- a statement of how many times the experiment shown was independently replicated in the laboratory.
- definitions of statistical methods and measures:
  - common tests, such as t-test (please specify whether paired vs. unpaired), simple χ2 tests, Wilcoxon and Mann-Whitney tests, can be unambiguously identified by name only, but more complex techniques should be described in the methods section;
  - are tests one-sided or two-sided?
  - are there adjustments for multiple comparisons?
  - exact statistical test results, e.g., P values = x but not P values < x;
  - definition of 'center values' as median or average;
  - definition of error bars as s.d. or s.e.m.

Please complete ALL of the questions below.
Select "Not Applicable" only when the requested information is not relevant for your study.

Materials

Newly Created Materials	Information included in the manuscript?	In which section is the information available? (Reagents and Tools Table, Materials and Methods, Figures, Data Availability Section)
New materials and reagents need to be available; do any restrictions apply?	Not Applicable	Not applicable

Antibodies	Information included in the manuscript?	In which section is the information available? (Reagents and Tools Table, Materials and Methods, Figures, Data Availability Section)
For **antibodies** provide the following information:<br>- Commercial antibodies: RRID (if possible) or supplier name, catalogue number and or/clone number<br>- Non-commercial: RRID or citation	Yes	Catalogue numbers included in Materials and Methods

DNA and RNA sequences	Information included in the manuscript?	In which section is the information available? (Reagents and Tools Table, Materials and Methods, Figures, Data Availability Section)
**Short novel DNA or RNA including primers, probes**: provide the sequences.	Yes	Sequences are included in the text

Cell materials	Information included in the manuscript?	In which section is the information available? (Reagents and Tools Table, Materials and Methods, Figures, Data Availability Section)
**Cell lines:** Provide species information, strain. Provide accession number in repository **OR** supplier name, catalog number, clone number, and/**OR** RRID.	Yes	All cell lines information are included in the text
**Primary cultures:** Provide species, strain, sex of origin, genetic modification status.	Not Applicable	Not Applicable
Report if the cell lines were recently **authenticated** (e.g., by STR profiling) and tested for mycoplasma contamination.	Yes	All cell llines were negative for micoplasma contamination and regularly tested

Experimental animals	Information included in the manuscript?	In which section is the information available? (Reagents and Tools Table, Materials and Methods, Figures, Data Availability Section)
**Laboratory animals or Model organisms:** Provide species, strain, sex, age, genetic modification status. Provide accession number in repository **OR** supplier name, catalog number, clone number, **OR** RRID.	Yes	All information are included in the text
**Animal observed in or captured from the field:** Provide species, sex, and age where possible.	Not Applicable	Not Applicable
Please detail **housing and husbandry conditions**.	Yes	All information are included in the text

Plants and microbes	Information included in the manuscript?	In which section is the information available? (Reagents and Tools Table, Materials and Methods, Figures, Data Availability Section)
**Plants:** provide species and strain, ecotype and cultivar where relevant, unique accession number if available, and source (including location for collected wild specimens).	Not Applicable	Not Applicable
**Microbes:** provide species and strain, unique accession number if available, and source.	Not Applicable	Not Applicable

Human research participants	Information included in the manuscript?	In which section is the information available? (Reagents and Tools Table, Materials and Methods, Figures, Data Availability Section)
If collected and within the bounds of privacy constraints report on age, sex and gender or ethnicity for all study participants.	Not Applicable	Not Applicable

Core facilities	Information included in the manuscript?	In which section is the information available? (Reagents and Tools Table, Materials and Methods, Figures, Data Availability Section)
If your work benefited from core facilities, was their service mentioned in the acknowledgments section?	Yes	The Facility was mentioned in the text

Design

Study protocol	Information included in the manuscript?	In which section is the information available? (Reagents and Tools Table, Materials and Methods, Figures, Data Availability Section)
If study protocol has been **pre-registered, provide DOI in the manuscript**. For clinical trials, provide the trial registration number **OR** cite DOI.	Not Applicable	Not Applicable
Report the **clinical trial registration number** (at ClinicalTrials.gov or equivalent), where applicable.	Not Applicable	Not Applicable

Laboratory protocol	Information included in the manuscript?	In which section is the information available? (Reagents and Tools Table, Materials and Methods, Figures, Data Availability Section)
Provide DOI OR other citation details if **external detailed step-by-step protocols** are available.	Not Applicable	Not Applicable

Experimental study design and statistics	Information included in the manuscript?	In which section is the information available? (Reagents and Tools Table, Materials and Methods, Figures, Data Availability Section)
Include a statement about **sample size** estimate even if no statistical methods were used.	Yes	All information are included in the text
Were any steps taken to minimize the effects of subjective bias when allocating animals/samples to treatment (e.g. **randomization procedure**)? If yes, have they been described?	Not Applicable	Not Applicable
Include a statement about **blinding** even if no blinding was done.	Not Applicable	Not Applicable
Describe **inclusion/exclusion criteria** if samples or animals were excluded from the analysis. Were the criteria pre-established?<br><br>If sample or data points were omitted from analysis, report if this was due to attrition or intentional exclusion and provide justification.	Not Applicable	Not Applicable
For every figure, are **statistical tests** justified as appropriate? Do the data meet the assumptions of the tests (e.g., normal distribution)? Describe any methods used to assess it. Is there an estimate of variation within each group of data? Is the variance similar between the groups that are being statistically compared?	Yes	All information are included in the text

Sample definition and in-laboratory replication	Information included in the manuscript?	In which section is the information available? (Reagents and Tools Table, Materials and Methods, Figures, Data Availability Section)
In the figure legends: state number of times the experiment was **replicated** in laboratory.	Yes	All information are included in the Figure Legends
In the figure legends: define whether data describe **technical or biological replicates**.	Yes	All information are included in the Figure Legends

Ethics

Ethics	Information included in the manuscript?	In which section is the information available? (Reagents and Tools Table, Materials and Methods, Figures, Data Availability Section)
Studies involving **human participants**: State details of **authority granting ethics approval** (IRB or equivalent committee(s), provide reference number for approval.	Not Applicable	Not Applicable
Studies involving **human participants**: Include a statement confirming that **informed consent** was obtained from all subjects and that the experiments conformed to the principles set out in the WMA Declaration of Helsinki and the Department of Health and Human Services Belmont Report.	Not Applicable	Not Applicable
Studies involving **human participants:** For publication of **patient photos**, include a statement confirming that consent to publish was obtained.	Not Applicable	Not Applicable
Studies involving experimental **animals**: State details of **authority granting ethics approval** (IRB or equivalent committee(s), provide reference number for approval. Include a statement of compliance with ethical regulations.	Yes	All studies on fish were conducted in strict accordance with the Institutional Guidelines for animal research. Ethical approval is not requested for this study that involves analyses only up hatching of embryos because at this stage of development, they are not capable of independent feeding in accordance with the law on animal experimentation by the Italian Ministry of Health; Department of Public Health, Animal Health, Nutrition, and Food Safety (D.Lgs. 26/2014).
Studies involving **specimen and field samples:** State if relevant **permits** obtained, provide details of authority approving study; if none were required, explain why.	Not Applicable	Not Applicable

Dual Use Research of Concern (DURC)	Information included in the manuscript?	In which section is the information available? (Reagents and Tools Table, Materials and Methods, Figures, Data Availability Section)
Could your study fall under dual use research restrictions? Please check biosecurity documents and list of **select agents and toxins** (CDC): https://www.selectagents.gov/sat/list.htm	Not Applicable	Not Applicable
If you used a select agent, is the security level of the lab appropriate and reported in the manuscript?	Not Applicable	Not Applicable
If a study is subject to dual use research of concern regulations, is the name of the **authority granting approval and reference number** for the regulatory approval provided in the manuscript?	Not Applicable	Not Applicable

Reporting

The MDAR framework recommends adoption of discipline-specific guidelines, established and endorsed through community initiatives. Journals have their own policy about requiring specific guidelines and recommendations to complement MDAR.

Adherence to community standards	Information included in the manuscript?	In which section is the information available? (Reagents and Tools Table, Materials and Methods, Figures, Data Availability Section)
State if relevant guidelines or checklists (e.g., **ICMJE, MIBBI, ARRIVE, PRISMA**) have been followed or provided.	Not Applicable	Not Applicable
For **tumor marker prognostic studies,** we recommend that you follow the **REMARK** reporting guidelines (see link list at top right). See author guidelines, under 'Reporting Guidelines'. Please confirm you have followed these guidelines.	Not Applicable	Not Applicable
For **phase II and III randomized controlled trials**, please refer to the **CONSORT** flow diagram (see link list at top right) and submit the CONSORT checklist (see link list at top right) with your submission. See author guidelines, under 'Reporting Guidelines'. Please confirm you have submitted this list.	Not Applicable	Not Applicable

Data Availability

Data availability	Information included in the manuscript?	In which section is the information available? (Reagents and Tools Table, Materials and Methods, Figures, Data Availability Section)
Have **primary datasets** been deposited according to the journal's guidelines (see 'Data Deposition' section) and the respective accession numbers provided in the Data Availability Section?	Not Applicable	Not Applicable
Were **human clinical and genomic datasets** deposited in a public access-controlled repository in accordance to ethical obligations to the patients and to the applicable consent agreement?	Not Applicable	Not Applicable
Are **computational models** that are central and integral to a study available without restrictions in a machine-readable form? Were the relevant accession numbers or links provided?	Not Applicable	Not Applicable
If publicly available data were reused, provide the respective **data citations in the reference list**.	Not Applicable	Not Applicable
